# Evidence for the Extensive Conservation of Mechanisms of Ovule Integument Development Since the Most Recent Common Ancestor of Living Angiosperms

**DOI:** 10.3389/fpls.2018.01352

**Published:** 2018-09-19

**Authors:** Gontran Arnault, Aurélie C. M. Vialette, Amélie Andres-Robin, Bruno Fogliani, Gildas Gâteblé, Charles P. Scutt

**Affiliations:** ^1^Laboratoire Reproduction et Deìveloppement des Plantes, École Normale Supérieure de Lyon, Université Claude Bernard Lyon 1, CNRS, INRA, Université de Lyon, Lyon, France; ^2^Équipe ARBOREAL, “Agriculture Biodiversité et Valorisation”, Institut Agronomique Néo-Calédonien (IAC), Païta, New Caledonia

**Keywords:** integument, *Amborella trichopoda*, ovule, angiosperms, HD-ZIP III, YABBY, KANADI, AUXIN RESPONSE FACTOR

## Abstract

The ovules and seeds of most angiosperm groups are enclosed by two integuments, whose evolutionary origins are considerably separated in time, as the inner integument arose over 300 million years ago (MYA) in an ancestor of all living seed plants, while the outer integument arose, perhaps as recently as 164 MYA, in an ancestor of all living angiosperms. Studies of the model angiosperm *Arabidopsis thaliana* indicate that the mechanisms of development of the inner and outer integuments depend on largely different sets of molecular players. However, it was not known, in most cases, whether these differences were already present in early flowering plants, or arose later in the *Arabidopsis* lineage. Here, we analyze the expression patterns of integument regulators in *Amborella trichopoda*, the likely sister to all other living angiosperms. The data obtained indicate that regulators of the YABBY, KANADI, and homeodomain-leucine zipper class III transcription factor families have largely conserved their integument-specific expression profiles in the *Amborella* and *Arabidopsis* lineages since the most recent common ancestor (MRCA) of living angiosperms. We identified only one case, involving the paralogous genes *ETTIN* and *AUXIN RESPONSE FACTOR4*, in which integument-specific expression patterns had clearly diverged between *Amborella* and *Arabidopsis*. We use the data obtained to partially reconstruct molecular mechanisms of integument development in the MRCA of living angiosperms and discuss our findings in the context of alternative hypotheses for the origin of the angiosperm outer integument.

## Introduction

The ovules and seeds of most seed plants are covered by one or two integuments. These maternal tissues function to (1) protect the internal tissues of the ovule and later the seed, (2) define a route, via the micropyle, for pollen or pollen-tube entry, and (3) contribute in many cases to the regulation of seed hydration and dormancy (Linkies et al., 2010). Gymnosperms possess a single integument, whereas the majority of angiosperm groups, including the most basally diverging of these, possess two integuments, of which the inner integument is considered homologous to the single integument of gymnosperms. The origin of the inner integument therefore dates from before the separation of the living angiosperm and gymnosperm lineages, believed to have occurred over 300 million years ago (MYA), while the outer integument must have arisen somewhere along the angiosperm stem lineage, perhaps as recently as ∼164 MYA: a reasonable estimated date for the most recent common ancestor (MRCA) of living angiosperms (Moore et al., 2010). Within angiosperms, several groups have undergone a secondary reduction to a single integument (Endress, 2011), notably including the asterids, which contain over 80,000 species.

Much of what is known of the molecular mechanisms of integument development comes from the study of the model angiosperm *Arabidopsis thaliana* (hereafter referred to as *Arabidopsis*), which possesses anatropous, bitegmic ovules. Ovule initiation in *Arabidopsis* begins with the expression of the homeobox transcription factor *WUSCHEL* (*WUS*) (see Mathews and Kramer, 2012), which is known to promote cell proliferation in meristematic tissues. The ovule thus shares characteristics of meristems, such as the stem apical meristem and floral meristem, both of which produce lateral organs on their flanks. In the case of the bitegmic ovule, the lateral organs produced in this way are the inner and outer integuments, which arise from the chalazal tissue in the central region of the elongating ovule primordium. The inner integument arises first, followed closely by the outer integument (Endress, 2011).

Early expression of *WUS* at the apex of the *Arabidopsis* ovule primordium defines the presumptive nucellar tissue. The outgrowth of the integuments from the chalazal region, immediately below the nucellus, depends on the expression of three homeodomain-leucine zipper class III (HD-ZIP III) transcription factors, *PHABULOSA* (*PHB*), *PHAVOLUTA* (*PHV*), and *CORONA* (*CNA*) (Kelley et al., 2009), which act redundantly, and together with other factors, to limit the basal spread of *WUS* expression (Yamada et al., 2016). Expression of these HD-ZIP III factors defines the adaxial (towards the growth axis) zone of the presumptive inner integument. The inner and outer integument primordia are associated with the formation of auxin maxima, and it has been proposed that *ETTIN/AUXIN RESPONSE FACTOR3* (*ETT/ARF3*) and *ABERRANT TESTA SHAPE/KANADI4* (*ATS/KAN4*), both of which are expressed in the abaxial (away from the growth axis) domain of the inner integument, act to remove auxin from the zone between the two integument primordia (Kelley et al., 2012). Interestingly, Lora et al. (2015) found that in certain species of *Prunus* whose ovules contain only one integument, expression of the *ETT* ortholog was absent from the chalaza and inner integument. These authors accordingly proposed the loss of *ETT* expression to represent a potential evolutionary mechanism for the reduction from two integuments to one in these species.

HD-ZIP III and KANADI family factors are believed to control abaxial–adaxial tissue polarity in lateral organs through the regulation of a common set of direct target genes, many of which are related to auxin signaling or dynamics (Reinhart et al., 2013; Huang et al., 2014). HD-ZIP III factors regulate these targets positively to promote adaxial tissue identity, while KANADI factors regulate the same genes negatively to promote abaxial tissue identity. In *Arabidopsis*, there is a clear division of labor between the inner and outer integuments, as *ATS* is the principal KANADI family member expressed in the inner integument (McAbee et al., 2006), while this role is played redundantly by *KAN1* and *KAN2* in the outer integument (Eshed et al., 2004; McAbee et al., 2006). In the HD-ZIP III family, *PHB, PHV*, and *CNA* are expressed specifically in the inner integument, while *REV* is expressed in both integuments (Sieber et al., 2004b; Kelley et al., 2009).

Tissue outgrowth in angiosperm lateral organs, including for example leaves and carpels, is typically associated with the expression of YABBY transcription factors. These factors are expressed in the abaxial tissue domain, and can act either positively or negatively on the transcription of downstream targets, apparently through protein–protein interactions with distinct sets of co-factors (Bonaccorso et al., 2012; Simon et al., 2017). Despite their abaxial-specific expression profiles, YABBYs are believed not to define abaxial tissue identity *per se*, but rather to facilitate lateral organ outgrowth by promoting the expression of *WUS*-related transcription factors in marginal meristems (Nakata et al., 2012). The YABBY gene *INNER NO OUTER* (*INO*), is expressed specifically in the abaxial zone of the *Arabidopsis* outer integument, and an orthologue of this gene shows a largely similar expression pattern in the basally diverging angiosperm *Nymphaea* (Nymphaeales, Nymphaeaceae) (Yamada et al., 2003, 2011), suggesting *INO*’s tissue-specific role to have been conserved from early stages of angiosperm evolution.

With very few exceptions (such as that of *INO*, mentioned above), the mechanisms of integument development have been investigated to date uniquely in *Arabidopsis*, and it is therefore not known, in most cases, which of these were already present in early flowering plants and which evolved later in the *Arabidopsis* lineage. In the present work, we investigate the conservation of mechanisms of integument development from very early stages of angiosperm evolution by analyzing the expression patterns of the orthologs of *Arabidopsis* integument regulators in the basally diverging angiosperm *Amborella trichopoda* (hereafter referred to as *Amborella*). Most recent molecular phylogenetic studies place *Amborella*, the only known representative of Amborellales, as sister to all other living angiosperms (Stevens, 2001 onwards). According to these studies, Amborellales is the first of the three earliest diverging “ANA-grade” angiosperm orders, the remaining two being Nymphaeales and Austrobaileyales. *Amborella* is a dioecious scrambling shrub, endemic to the subtropical rainforests of the southern pacific island of New Caledonia. Its female flowers produce 5–6 unfused carpels, each containing a single, pendant, bitegmic ovule. These ovules are of more-or-less orthotropous symmetry, though show a distinct longitudinal curvature, which may be due to developmental constraints within the ovary, or may represent the residual effect of an ancestral anatropous symmetry (Endress and Igersheim, 2000).

Here, we focus on integument regulators of the YABBY, KANADI, HD-ZIP III, and ARF transcription factor families. We use gene expression patterns to identify molecular mechanisms of integument development that have probably been conserved since the MRCA of living flowering plants, and others that appear to have undergone changes in either the *Amborella* or *Arabidopsis* lineages. We discuss the data obtained in the context of alternative hypotheses for the origin of the outer integument in a distant common ancestor of living angiosperms.

## Materials and Methods

### Phylogenetic Reconstruction

Phylogenetic reconstructions in the KANADI and HD-ZIP III families were performed using a wide taxonomic sampling of angiosperms. Sequences for inclusion in phylogenetic analyses were selected by TBLASTN searching (Altschul et al., 1997) of the NCBI/NIH non-redundant nucleotide database^1^ and the *Amborella* genome database^2^ using *Arabidopsis* integument development proteins. Protein sequences were aligned using MUSCLE in the SeaView phylogeny package (Gouy et al., 2010). Sites were selected using G-BLOCKS, using all three options provided to minimize the stringency of selection. Phylogenies were generated from amino acid alignments in PhyML (Guindon et al., 2009) using the following parameters: model: LG; branch support: aLRT; amino acid equilibrium frequencies: model-given; invariable sites: optimized; across-site rate variation: optimized; tree searching operations: best of NNI and SPR; and starting tree: BioNJ.

### Gene Expression Studies

Full-length nucleotide sequences corresponding to *Amborella* orthologs or pro-orthologs of *Arabidopsis* integument regulators were PCR-amplified from an *Amborella* female flower cDNA library (Fourquin et al., 2005) using the primers shown in **Supplementary Table S1**. Digoxigenin-labeled riboprobes were generated from these and used in non-radioisotopic *in situ* hybridization to tissue sections of *Amborella* flower buds at approximately Stage 7 (Buzgo et al., 2004), and of female flowers at anthesis, using the protocol given by Vialette-Guiraud et al. (2011). Gene expression patterns were observed and photographed under bright field illumination using a Leica Axio Imager M2 inverted microscope fitted with a Leica AxioCam MRc digital camera.

### Anatomical Observations

*Amborella* ovule anatomy was revealed in sections of fixed female flowers, prepared and photographed as for *in situ* hybridization. These sections were stained with 0.05% (w/v) toluidine Blue-0 in 0.1 M sodium phosphate buffer (pH 6.8).

## Results

### Clear Orthologs of Several *Arabidopsis* Integument Regulators Can Be Identified in *Amborella*

Several previous studies have focused on the molecular phylogeny of gene families involved in the regulation of integument development. Accordingly, the *Amborella* ortholog of the *Arabidopsis* YABBY-family integument regulator *INO* has previously been reported (Finet et al., 2016), as have those of the paralogous and partially redundant auxin response family regulators *ETT* and *AUXIN RESPONSE FACTOR4* (*ARF4*) (Finet et al., 2010). However, published phylogenies of the KANADI and HD-ZIP III families have not to our knowledge previously included sequences from *Amborella*.

Phylogenetic reconstructions of the HD-ZIP III family in the present study (**Supplementary Figure S1**), generated from the protein alignment shown in **Supplementary Figure S2**, succeeded in identifying clear *Amborella* orthologs of the *Arabidopsis* genes *REV* and *CNA*, and a clear pro-ortholog of the *Arabidopsis* genes *PHB* and *PHV*, which accordingly appear to be derived from a duplication within the crown group of living angiosperms. In all these cases, the *Amborella* genes identified as orthologs or pro-orthologs of *Arabidopsis* integument regulators occupied basal positions within their respective clades, in agreement with the likely phylogenetic placement of *Amborella*.

Phylogenetic reconstruction of the KANADI family (**Supplementary Figure S3**), generated from the protein alignment shown in **Supplementary Figure S4**, succeeded in identifying a clear *Amborella* ortholog of the *Arabidopsis* inner integument regulator *ATS (KAN4)*, which grouped in a small basal clade of *ATS*-like sequences. Relationships of gene orthology between *Amborella* sequences and the *Arabidopsis* outer integument regulators *KAN1* and *KAN2* were less clear in phylogenetic reconstructions.

The results obtained in these reconstructions, combined with those of previously published work, permitted detailed analyses in the present study of the expression in *Amborella* female flower tissues of the genes: *Atr.INO* (XM_006840616), *Atr.ATS*, (XM_006856161.3), *AtrPHB/PHV* (XM_006845952.3), *Atr.REV* (XM_006846714.3), *Atr.ARF4* (XM_020671056), and *Atr.ETT* (XM_011627988.2). The longer alternative splicing variant of *Atr.ARF4* (Finet et al., 2010) was used for *in situ* hybridization studies.

### The *Amborella* Orthologue of the YABBY Gene *INO* Is Narrowly Expressed in the Abaxial Domain of the Outer Integument, As Is *INO* in *Arabidopsis*

**FIGURE 1:**
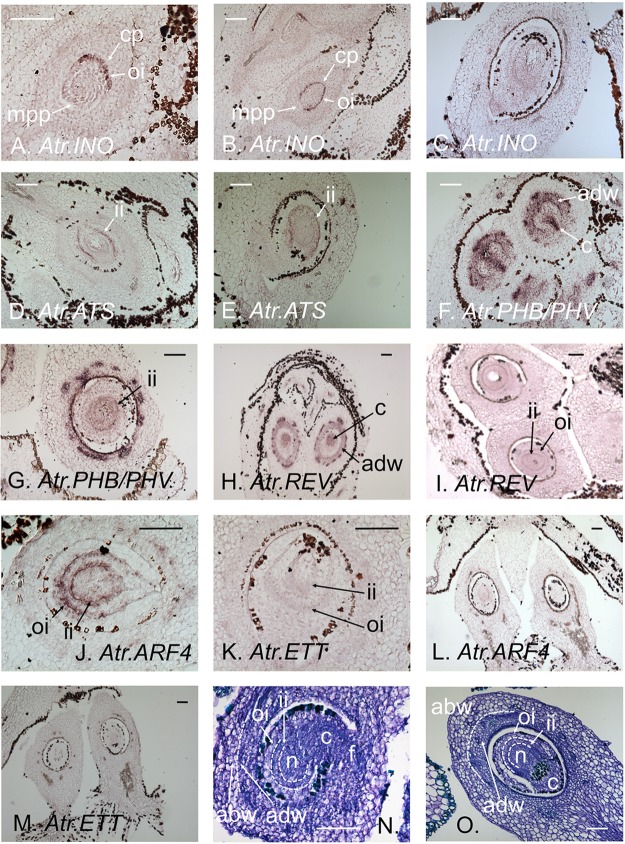
*In situ* hybridi**z**ations of integument regulators to female *Amborella* flower sections. **(A)** *Atr.INO* expression in the growing outer integument at early Stage 7. **(B)** *Atr.INO* expression in the near fully grown outer integument at late Stage 7. **(C)** *Atr.INO* expression is no longer apparent in the outer integument (LS) in mature flowers. **(D)** *Atr.ATS* expression in the inner integument at floral bud Stage 7. **(E)** *Atr.ATS* expression in the inner integument persists in the mature flower. **(F)** Narrow *Atr.PHB/PHV* expression in the chalaza and strong expression in the adaxial domain of the ovary wall at early Stage 7. **(G)** Weak *Atr.PHB/PHV* expression persists in the abaxial domain of the inner integument at late Stage 7. **(H)** *Atr.REV* is extensively expressed in the chalaza, and strongly expressed in the adaxial domain of the ovary wall at early Stage 7. **(I)** Weak *Atr.REV* expression persists in the adaxial domains of both integuments at late Stage 7. **(J)** *Atr.ARF4* expression in the abaxial domain of both integuments at early Stage 7. **(K)** Very weak *Atr.ETT* expression in the abaxial domain of both integuments at early Stage 7. **(L)** *Atr.ARF4* expression is no longer apparent in the integuments in the mature flower. **(M)** *Atr.ETT* expression is no longer apparent in the integuments in the mature flower. **(N**,**O)** Toluidine-blue stained sections of Stage 7 and mature ovules, respectively, showing anatomical details. Tissues zones in panels **(N,O)** have been delimited with dotted white lines for clarity. Developmental stages are as defined by Buzgo et al. (2004). abw, abaxial domain of the ovary wall**;** adw, adaxial domain of the ovary wall**;** c, chalaza**;** cp, chalazal pole**;** f, funiculus**;** ii, inner integument**;** mpp, micropyle pole**;** n, nucellus; and oi, outer integument. Scale bars = 100 μm.

**FIGURE 2:**
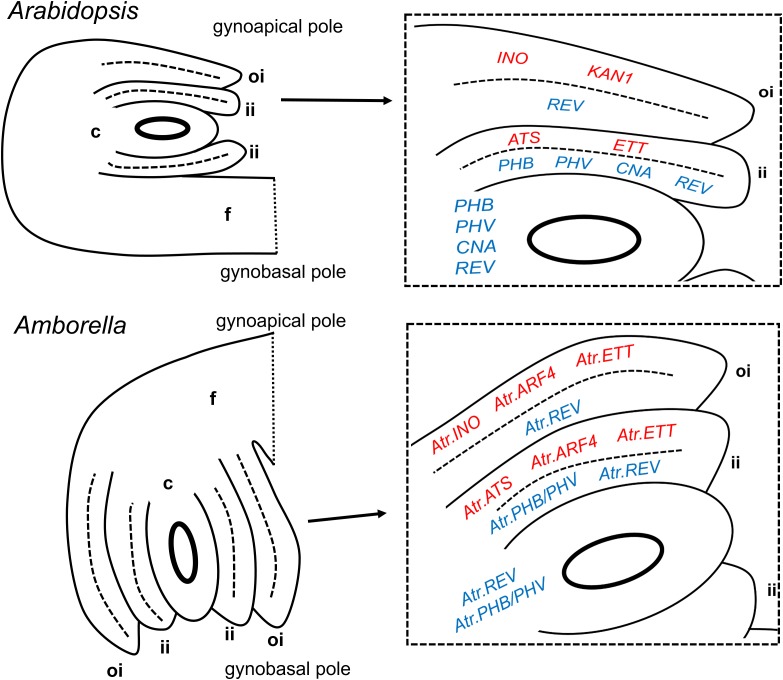
Summary of expression patterns of ovule integument regulators in *Arabidopsis thaliana* and *Amborella trichopoda*. Abaxial-promoting genes are shown in red in enlarged diagrams, adaxial-promoting genes are shown in blue. Mature ovules are shown, though the genes indicated may be maximally expressed in the tissues shown at earlier stages. Entire ovules (on the left) are shown in their natural orientations relative to the longitudinal axis of the gynoecium/carpel. c, chalaza; f, funiculus; ii, inner integument; and oi = outer integument.

*Atr.INO* proved to be specifically expressed in the most abaxial cell layer of the outer integument. Its expression was first noted in the outer integument, towards the chalazal-end of the ovary (**Figure 1A**), spreading towards the micropyle-end with the growth of the outer integument (**Figure 1B**). This expression pattern closely resembles that of *INO* in *Arabidopsis*. *Atr.INO* expression was stronger in early stages of *Amborella* ovule development, becoming absent in the mature ovule (**Figure 1C**). These data reinforce observations in *Nymphaea* (Yamada et al., 2003) that already suggested *Arabidopsis INO* to have conserved its function in outer integument development from early stages of angiosperm evolution. Expression patterns of *INO* orthologs in *Arabidopsis* and *Amborella* ovules, together with those of the other factors studied (below), are summarized in **Figure 2**.

### The *Amborella* Orthologue of the KANADI Family Gene *ATS (KAN4)* Is Expressed Abaxially in the Inner Integument, As Is *ATS* in *Arabidopsis*

*Atr.ATS* was found to be expressed specifically in the abaxial domain of the inner integument (**Figure 1D**), as is *ATS* in *Arabidopsis*. This expression persisted into the final stages of ovule development in almost mature flowers (**Figure 1E**). No expression of *Atr.ATS* was apparent in any female reproductive tissue other than the inner integument. These data strongly suggest *ATS* orthologs to have conserved their function in inner integument development in both the *Amborella* and *Arabidopsis* lineages since the MRCA of living flowering plants.

### *Amborella* HD-ZIP III Genes Show Integument-Specific Profiles Similar to Those of Their *Arabidopsis* Orthologs

We found *Atr.PHB/PHV*, the *Amborella* pro-ortholog of *Arabidopsis PHB* and *PHV*, to be strongly expressed at early stages of development in the chalazal region of the ovule, which gives rise to the integuments, and very strongly expressed in the adaxial tissues of the ovary wall (**Figure 1F**). At later developmental stages, *Atr.PHB/PHV* appeared weakly expressed in the adaxial zone of the inner integument (**Figure 1G**). At early stages of ovule development, *Atr.REV* showed a similar expression pattern to *Atr.PHB/PHV* in the chalaza and ovary wall, though arguably with a slightly wider expression in the chalaza (**Figure 1H**). At later stages of ovule development, reduced levels of *Atr.REV* expression were visible in the adaxial regions of both integuments (**Figure 1I**). The association of *Atr.PHB/PHV* expression with the inner integument, and that of *Atr.REV* with both integuments (**Figures 1G,I**), suggests the paralog-specific conservation of function between the *Amborella* and *Arabidopsis* lineages since their separation at the base of the living flowering plant clade. *Atr.PHB/PHV* and *Atr.REV* expression patterns are nonetheless very similar, and these genes are clearly expressed together in parts of the chalaza, the inner integument and the abaxial region of the ovary wall.

### The *Amborella* Orthologs of the Paralogous Auxin Response Factors *ETT* and *ARF4* Are Expressed Abaxially in Both Integuments, Showing Partial Conservation With Expression Patterns in *Arabidopsis*

*ETT* and *ARF4* act redundantly in the definition of abaxial tissue identity in the *Arabidopsis* leaf (Pekker et al., 2005), though *ETT* appears to play the major role in this process in both the carpel wall and inner integument. An earlier study of the orthologs of these factors in *Amborella* (Finet et al., 2010) suggested that *Atr.ARF4* may show a higher level of expression than *Atr.ETT* in female reproductive tissues and perhaps therefore play the major developmental role. In the present study, both *Atr.ARF4* and *Atr.ETT* were found to be expressed at early stages in the abaxial domain of both integuments (**Figures 1J,K**), with *Atr.ARF4* giving the stronger hybridi**z**ation signal. Expression levels of both genes diminished in mature integuments (**Figures 1L,M**). A switch may therefore have occurred in the major paralog active in the inner integument, with *ETT* playing the principal role in *Arabidopsis*, while *Atr.ARF4* is more highly expressed in *Amborella*. In addition, whereas the role of *ETT* is limited to the inner integument in *Arabidopsis, Atr.ARF4* and *Atr.ETT* are expressed in the abaxial domains of both integuments in *Amborella*.

It is interesting to note that *Arabidopsis* ETT has been found to interact physically with ATS, both of which are expressed specifically in the inner integument (Kelley et al., 2012). Atr.ETT and Atr.ARF4 may similarly interact with Atr.ATS in the *Amborella* inner integument, but this interaction could not occur in the outer integument as *Atr.ATS* is not apparently expressed in that tissue (**Figures 1D,E**). Of course, Atr.ETT and Atr.ARF4 might interact in the outer integument with other *Amborella* KANADI proteins (**Supplementary Figure S4**) whose expression patterns were not investigated in the present study.

Toluidine-blue stained sections of the carpel wall and ovule in a Stage 7 (**Figure 1N**) and a mature (**Figure 1O**) female flower are provided to help interpret anatomical details of *in situ* hybridization images shown in **Figures 1A–M**.

## Discussion

### Reconstructed Gene Expression Patterns Suggest That Largely Distinct Sets of Regulators Controlled Inner and Outer Integument Development in Early Flowering Plants, As In Present-Day *Arabidopsis*

The data presented here strongly suggest the extensive conservation of integument-specific developmental roles of genes of the YABBY, KANADI, and HD-ZIP III families in both the *Amborella* and *Arabidopsis* lineages since the MRCA of living flowering plants. It was previously known that inner and outer integument development in *Arabidopsis* depended on substantially distinct sets of molecular players. The current study provides evidence that most of these differences in molecular controls are ancient, and already existed in early flowering plants. The only clear exception noted in the present study, in which molecular mechanisms seem to have diverged in one or both of the *Amborella* and *Arabidopsis* lineages, concerns the paralogous ARF family members *ETT* and *ARF4*. *ETT* contributes to abaxial tissue identity uniquely in the inner integument of *Arabidopsis*, whereas orthologs of both *ETT* and *ARF4* are expressed in the abaxial domain of both integuments in *Amborella*. The inner integument expression of these factors may, as for that of *ETT* in *Arabidopsis*, act to prevent the coalescence of the two integument primordia. However, these factors may play a further role in the establishment and/or maintenance of abaxial/adaxial polarity in the *Amborella* outer integument, compared to their orthologs in *Arabidopsis*. Analysis of gene expression patterns in further ANA-grade taxa could be used to determine whether these ARF genes evolved more restricted inner integument-specific roles in the *Arabidopsis* lineage, or whether, conversely, their expression patterns became more generalized to both integuments, specifically in the *Amborella* lineage.

It is interesting to note that *ETT* expression is limited to the abaxial domain of the inner integument in bitegmic species of *Prunus* (rosid I clade) (Lora et al., 2015), as it is in *Arabidopsis*. If *ETT/ARF4* expression was lost from the outer integument in the *Arabidopsis* lineage, rather than gained in the *Amborella* lineage, it would therefore seem likely that this change happened before the divergence of the rosid I and II clades, which is estimated to have occurred some 105 MYA (Moore et al., 2010).

**FIGURE 3:**
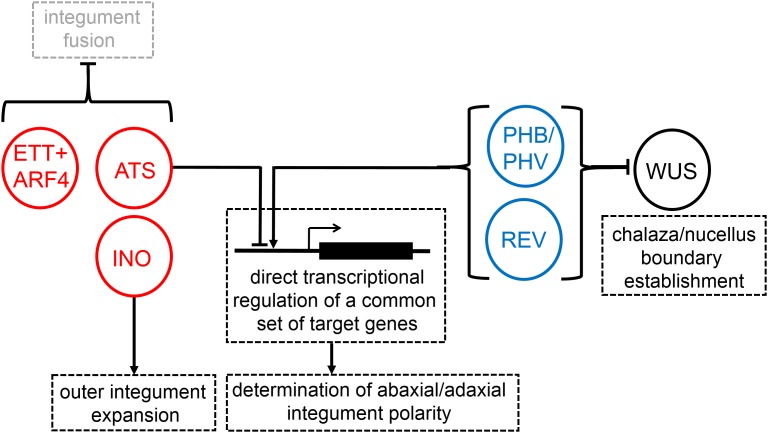
A partial reconstruction of the molecular mechanisms of integument development in the most recent common ancestor of living flowering plants. Pointed and barred arrows represent cases of positive and negative regulation, respectively. Abaxial-promoting factors are shown in red, adaxial-promoting factors are shown in blue.

We have used the data presented here, together with information from published studies, to construct a partial molecular model for the control of integument development in the MRCA of living angiosperms (**Figure 3**). Predicted intermolecular interactions shown in this model are based on functional data from *Arabidopsis* alone and the conservation of tissue-colocalization of the molecules concerned in *Amborella*.

### Implications of Reconstructed Integument Development Mechanisms for the Origin of the Outer Integument and Flowering Plants

Like the carpel, the outer integument is a pleisiomorphic feature of angiosperms. Two contrasting hypotheses have been proposed to account for the origin of this structure, prior to the radiation of living angiosperms. One of these hypotheses (reviewed by Doyle, 2008), based mainly on paleobotanical data, proposes that the angiosperms evolved from cupulate seed–ferns, possibly resembling the known fossil groups Caytoniales or Glossopteridales. In most known members of these taxa, several ovules occur within laminar cupules that are either borne on the margins of a female rachis (in Caytoniales), or emerge from the axil or midrib of a megasporophyll (in Glossopteridales). According to this “cupule hypothesis”, the bitegmic angiosperm ovule would have evolved from a unitegmic gymnosperm ovule by a reduction in the number of ovules-per-cupule to one, the cupule thereby becoming the outer integument. The Caytoniales-type cupule may be a particularly strong candidate as a precursor to the angiosperm outer integument as the orientation of the ovule within this type of cupule could have led directly to the anatropous ovule arrangement (Doyle, 2008), which may have been pleisiomorphic in angiosperms.

The main alternative hypothesis for the origin of the outer integument, as discussed by Mathews and Kramer (2012), is that this structure simply arose through a modular reiteration of the single integument already present in species along the angiosperm stem lineage. Indeed, the development of the outer and inner integuments proceeds in a remarkably similar manner in most angiosperms, and the overexpression of *WUS* leads, in transgenic *Arabidopsis*, to the production of supernumerary integuments (Gross-Hardt et al., 2002; Sieber et al., 2004a), demonstrating that extra integuments can be generated from pre-existing developmental mechanisms by a simple molecular change in an upstream regulator. Not all strong candidates for ancestors, or close stem-lineage relatives of the angiosperms, possessed cupules. Notably, Bennettitales, which possessed many angiosperm-like characteristics, including a perianth, a bisexual axis (in some taxa), non-saccate pollen, net-veined leaves, and oleananes (highly resistant terpenoid compounds, also present in angiosperms), possessed no structure resembling an ovule-enclosing cupule. A mechanism based on the modular reiteration of a pre-existing single integument might, therefore, explain the origin of the angiosperm outer integument in a potential bennettialian ancestor.

The data presented here strongly suggest that the precise mechanisms of integument development were already substantially distinct in the inner and outer integuments some 164 MYA in the MRCA of living flowering plants. This conclusion clearly favors the hypothesis of a distant or indirect homology between the inner and outer integuments, such as might be explained by the more direct origin of the outer integument from an ovule-containing seed–fern cupule. It does not provide support for the possible origin of the outer integument by a modular reiteration of the inner integument, as this mechanism would be expected to yield two integuments whose development initially depended, in early flowering plants, on near-identical sets of molecular regulators.

## Author Contributions

GA performed the experiments and phylogenetic analyses. AV supervised the experimental work and phylogenetic analyses. AA-R supervised the experimental work. BF and GG provided the plant material and contributed to writing the paper. CS planned and supervised the research and wrote the paper.

## Conflict of Interest Statement

The authors declare that the research was conducted in the absence of any commercial or financial relationships that could be construed as a potential conflict of interest.

## Footnotes

1 https://blast.ncbi.nlm.nih.gov/Blast.cgi

2 http://genomevolution.org/CoGe/

## Supplementary Material

FIGURE S1 Maximum likelihood phylogeny of angiosperm HD-ZIP III family proteins. aLRT branch-support values are shown at nodes. *Amborella* and *Arabidopsis* sequences mentioned in the text are highlighted. Key to species names: *Ara tha*: *Arabidopsis thaliana*; *AmTr*: *Amborella trichopoda*; *Bra dis*: *Brachypodium distachyon*; *Bra nap*: *Brassica napus*; *Bra rapa*: *Brassica rapa*; *Cam sat*: *Camelina sativa*; *Cit sin*: *Citrus sinensis*; *Fra ves*: *Fragaria vesca*; *Gly max*: *Glycine max*; *Med tru*: *Medicago truncatula*; *Mus acu*: *Musa acuminata*; *Nel nuc*: *Nelumbo nucifera*; *Ory bra*: *Oryza brachyantha*; *Ory sat*: *Oryza sativa*; *Pop tri*: *Populus trichocarpa*; *Pru per*: *Prunus persica*; *Raph sat*: *Raphanus sativus*; *Ric com*: *Ricinus communis*; *Sol lyc*: *Solanum lycopersicum*; *Sol tub*: *Solanum tuberosum*; *The cac*: *Theobroma cacao*; *Vig ang*: *Vigna angularis*; *Vig rad*: *Vigna radiate*; *Vit vin*: *Vitis vinifera*; and *Zea may*: *Zea mays*.

FIGURE S2 Alignment of angiosperm HD-ZIP III family proteins used to produce the phylogeny shown in **Supplementary Figure 1**. Sites used in phylogenetic analysis are underlined.

FIGURE S3 Maximum likelihood phylogeny of angiosperm KANADI family proteins. aLRT branch-support values are shown at nodes. *Amborella* and *Arabidopsis* sequences mentioned in the text are highlighted. Key to species names: *Ara tha*: *Arabidopsis thaliana*; *AmTr*: *Amborella trichopoda*; *Bra dis*: *Brachypodium distachyon*; *Bra nap*: *Brassica napus*; *Bra rapa*: *Brassica rapa*; *Cam sat*: *Camelina sativa*; *Cit sin*: *Citrus sinensis*; *Fra ves*: *Fragaria vesca*; *Gly max*: *Glycine max*; *Med tru*: *Medicago truncatula*; *Mus acu*: *Musa acuminate*; *Nel nuc*: *Nelumbo nucifera*; *Ory bra*: *Oryza brachyantha*; *Ory sat*: *Oryza sativa*; *Pop tri*: *Populus trichocarpa*; *Pru per*: *Prunus persica*; *Raph sat*: *Raphanus sativus*; *Ric com*: *Ricinus communis*; *Sol lyc*: *Solanum lycopersicum*; *Sol tub*: *Solanum tuberosum*; *The cac*: *Theobroma cacao*; *Vig ang*: *Vigna angularis*; *Vig rad*: *Vigna radiate*; *Vit vin*: *Vitis vinifera;* and *Zea may*: *Zea mays*.

FIGURE S4 Alignment of angiosperm KANADI family proteins used to produce the phylogeny shown in **Supplementary Figure 3**. Sites used in phylogenetic analysis are underlined.

TABLE S1 Oligonucleotide primers used to amplify *Amborella* integument regulators.
