# Supplementary figures and images for: Evidence for the Extensive Conservation of Mechanisms of Ovule Integument Development Since the Most Recent Common Ancestor of Living Angiosperms
Source: Front Plant Sci. 2018 Sep 19;9:1352. doi: 10.3389/fpls.2018.01352 (PMC6156155; doi:10.3389/fpls.2018.01352)

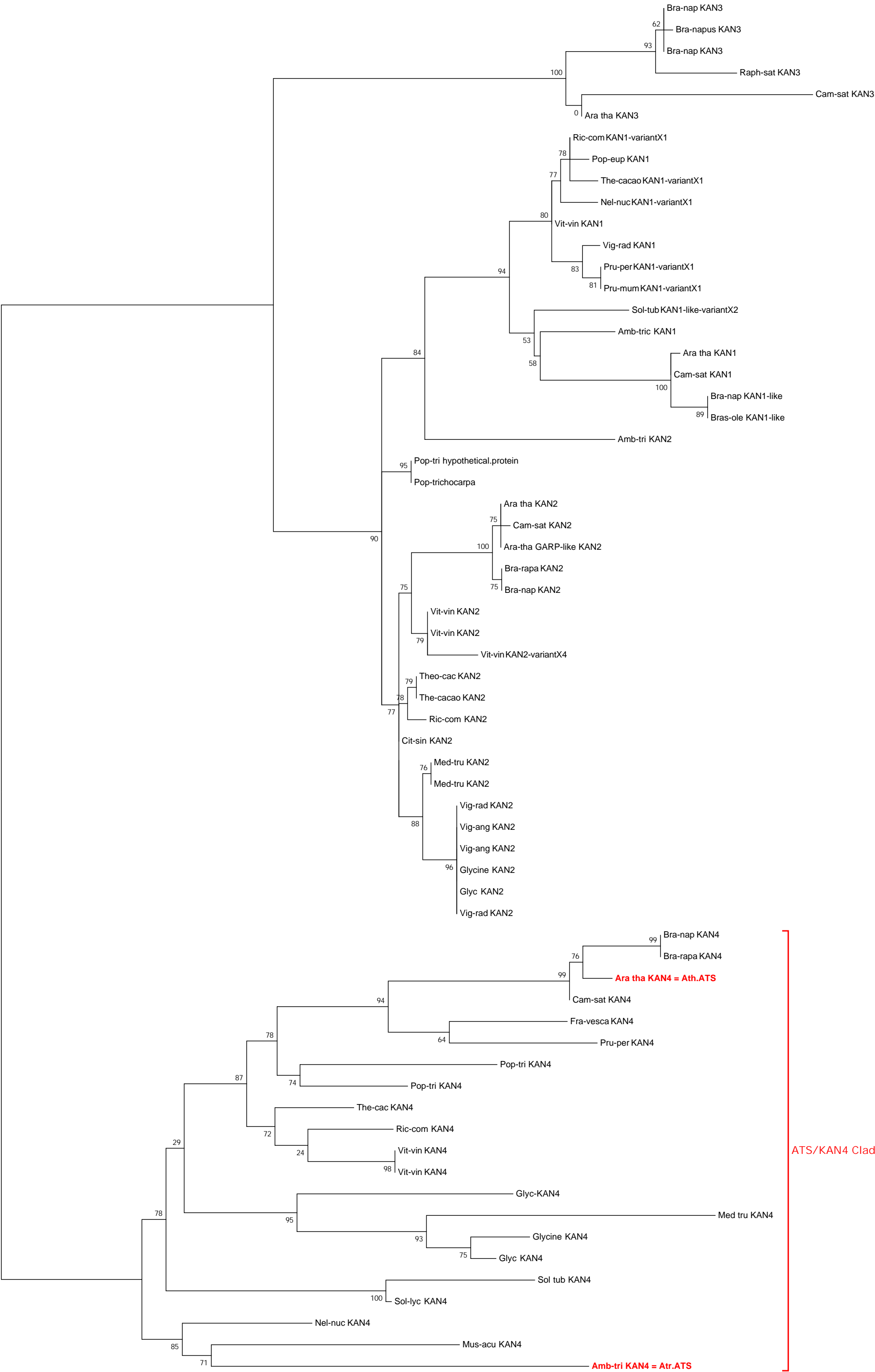

0.1

Supplement: FIGURE S1 — Maximum likelihood phylogeny of angiosperm HD-ZIP III family proteins. aLRT branch-support values are shown at nodes. Amborella and Arabidopsis sequences mentioned in the text are highlighted. Key to species names: Ara tha: Arabidopsis thaliana; AmTr: Amborella trichopoda; Bra dis: Brachypodium distachyon; Bra nap: Brassica napus; Bra rapa: Brassica rapa; Cam sat: Camelina sativa; Cit sin: Citrus sinensis; Fra ves: Fragaria vesca; Gly max: Glycine max; Med tru: Medicago truncatula; Mus acu: Musa acuminata; Nel nuc: Nelumbo nucifera; Ory bra: Oryza brachyantha; Ory sat: Oryza sativa; Pop tri: Populus trichocarpa; Pru per: Prunus persica; Raph sat: Raphanus sativus; Ric com: Ricinus communis; Sol lyc: Solanum lycopersicum; Sol tub: Solanum tuberosum; The cac: Theobroma cacao; Vig ang: Vigna angularis; Vig rad: Vigna radiate; Vit vin: Vitis vinifera; and Zea may: Zea mays. [file Image_1.pdf]

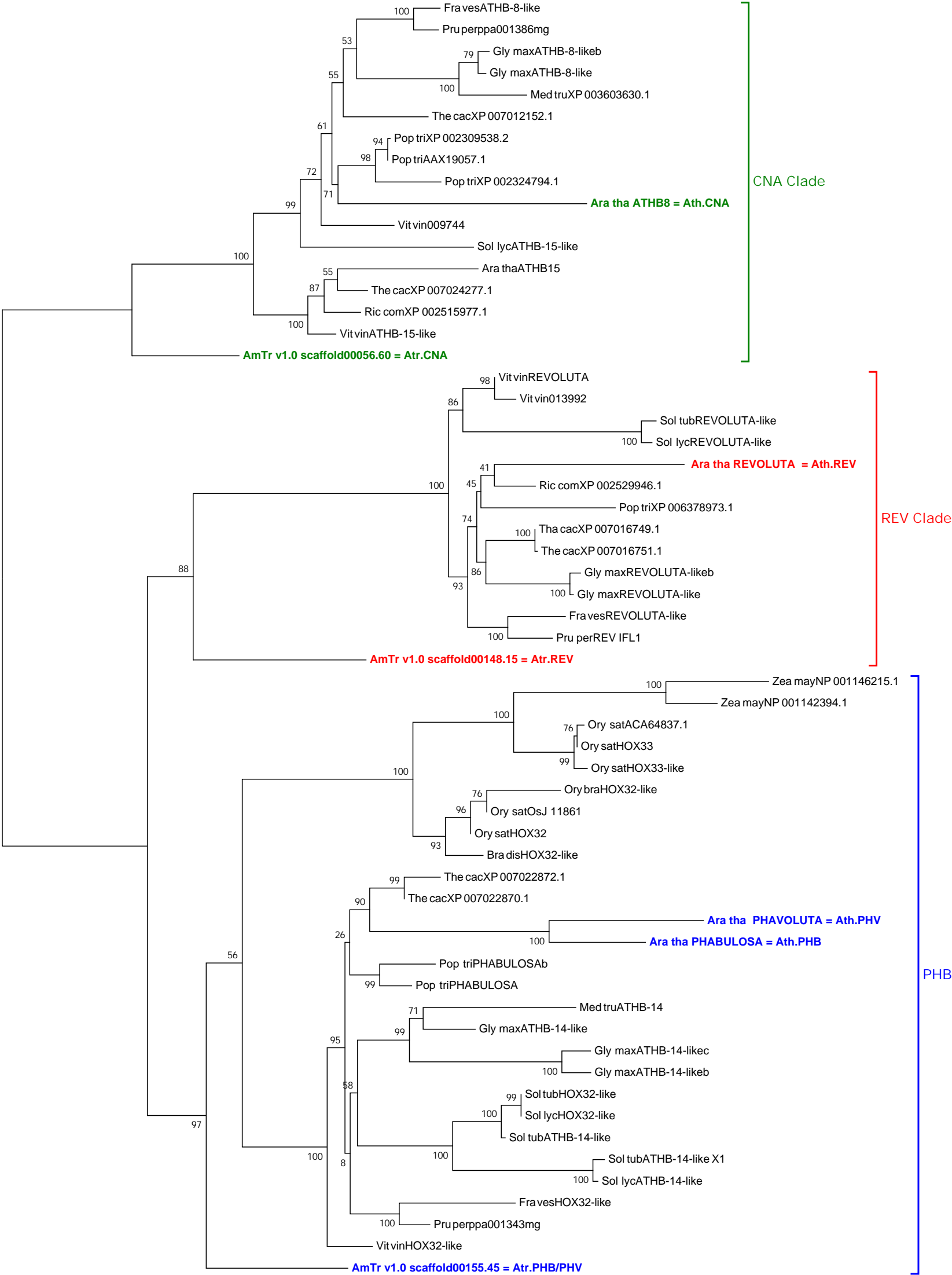

0.05

Supplement: FIGURE S3 — Maximum likelihood phylogeny of angiosperm KANADI family proteins. aLRT branch-support values are shown at nodes. Amborella and Arabidopsis sequences mentioned in the text are highlighted. Key to species names: Ara tha: Arabidopsis thaliana; AmTr: Amborella trichopoda; Bra dis: Brachypodium distachyon; Bra nap: Brassica napus; Bra rapa: Brassica rapa; Cam sat: Camelina sativa; Cit sin: Citrus sinensis; Fra ves: Fragaria vesca; Gly max: Glycine max; Med tru: Medicago truncatula; Mus acu: Musa acuminate; Nel nuc: Nelumbo nucifera; Ory bra: Oryza brachyantha; Ory sat: Oryza sativa; Pop tri: Populus trichocarpa; Pru per: Prunus persica; Raph sat: Raphanus sativus; Ric com: Ricinus communis; Sol lyc: Solanum lycopersicum; Sol tub: Solanum tuberosum; The cac: Theobroma cacao; Vig ang: Vigna angularis; Vig rad: Vigna radiate; Vit vin: Vitis vinifera; and Zea may: Zea mays. [file Image_3.pdf]
