# Supplementary material for: Evidence for the Extensive Conservation of Mechanisms of Ovule Integument Development Since the Most Recent Common Ancestor of Living Angiosperms
Source: Front Plant Sci. 2018 Sep 19;9:1352. doi: 10.3389/fpls.2018.01352 (PMC6156155; doi:10.3389/fpls.2018.01352)
Supplement: FIGURE S2 — Alignment of angiosperm HD-ZIP III family proteins used to produce the phylogeny shown in Supplementary Figure 1. Sites used in phylogenetic analysis are underlined. [file Image_2.pdf]

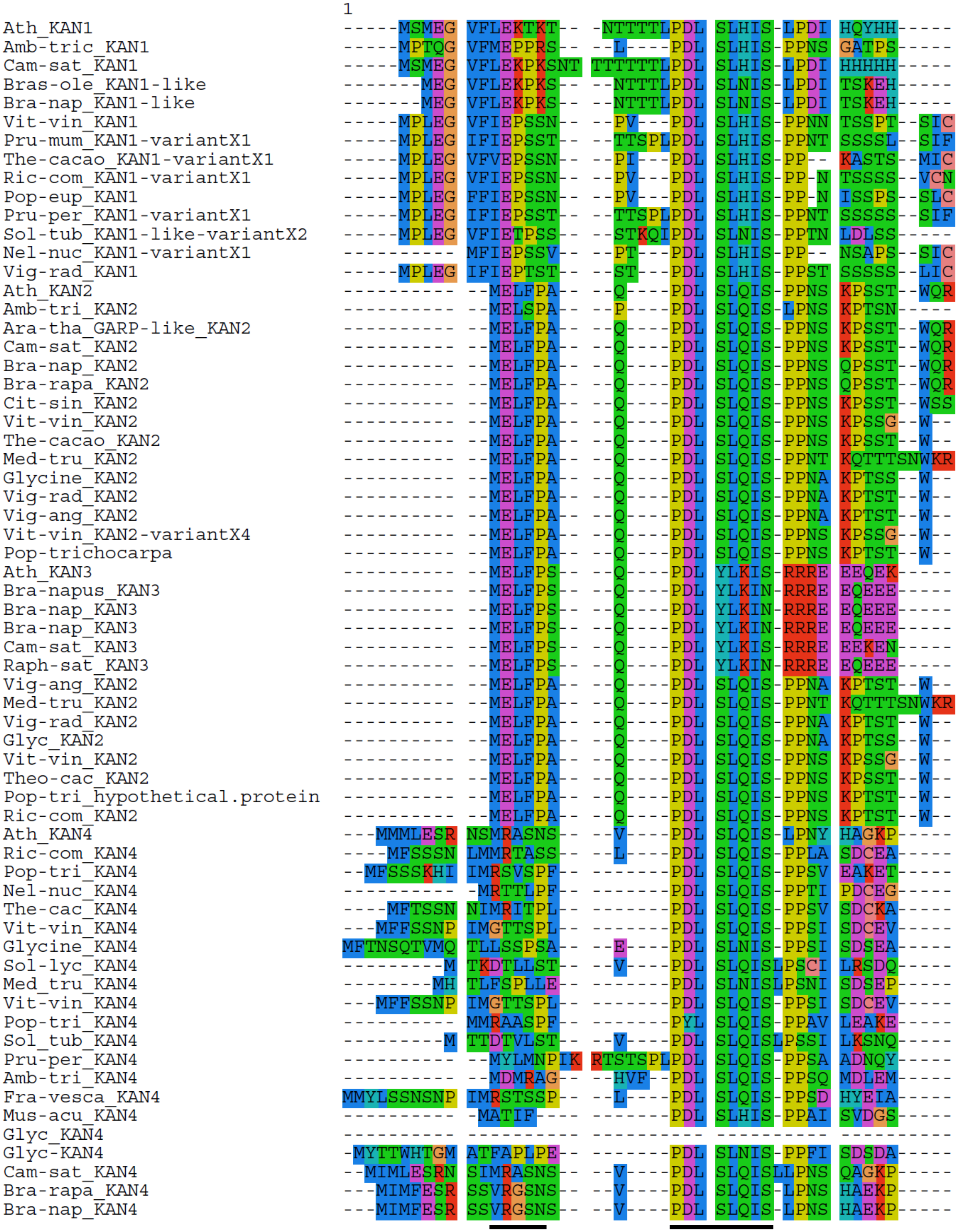
1
Ath_KAN1 -----MSMEG VFLEKTKT-- -NTTTTLPDL SLHIS-LPDI HQYHH-----
Amb-tric_KAN1 -----MPTQG VFMEPPRS-- --L----PDL SLHIS-PPNS GATPS-----
Cam-sat_KAN1 -----MSMEG VFLEKPKSNT TTTTTTLPDL SLHIS-LPDI HHHHH-----
Bras-ole_KAN1-like -------MEG VFLEKPKS-- --NTTTLPDL SLNIS-LPDI TSKEH-----
Bra-nap_KAN1-like -------MEG VFLEKPKS-- --NTTTLPDL SLNIS-LPDI TSKEH-----
Vit-vin_KAN1 -----MPLEG VFIEPSSN-- --PV---PDL SLHIS-PPNN TSSPT--SIC
Pru-mum_KAN1-variantX1 -----MPLEG IFIEPSST-- --TTSPLPDL SLHIS-PPNT SSSSL--SIF
The-cacao_KAN1-variantX1 -----MPLEG VFVEPSSN-- --PI---PDL SLHIS-PP-- KASTS--MIC
Ric-com_KAN1-variantX1 -----MPLEG VFIEPSSN-- --PV---PDL SLHIS-PP-N TSSSS--VCN
Pop-eup_KAN1 -----MPLEG FFIEPSSN-- --PV---PDL SLHIS-PP-N ISSPS--SLC
Pru-per_KAN1-variantX1 -----MPLEG IFIEPSST-- --TTSPLPDL SLHIS-PPNT SSSSS--SIF
Sol-tub_KAN1-like-variantX2 -----MPLEG VFIETPSS-- --STKQIPDL SLNIS-PPTN LDLSS-----
Nel-nuc_KAN1-variantX1 ---------- MFIEPSSV-- --PT---PDL SLHIS-PP-- NSAPS--SIC
Vig-rad_KAN1 -----MPLEG IFIEPTST-- --ST---PDL SLHIS-PPST SSSSS--LIC
Ath_KAN2 ---------- --MELFPA-- --Q----PDL SLQIS-PPNS KPSST--WQR
Amb-tri_KAN2 ---------- --MELSPA-- --P----PDL SLQIS-LPNS KPTSN-----
Ara-tha_GARP-like_KAN2 ---------- --MELFPA-- --Q----PDL SLQIS-PPNS KPSST--WQR
Cam-sat_KAN2 ---------- --MELFPA-- --Q----PDL SLQIS-PPNS KPSST--WQR
Bra-nap_KAN2 ---------- --MELFPA-- --Q----PDL SLQIS-PPNS QPSST--WQR
Bra-rapa_KAN2 ---------- --MELFPA-- --Q----PDL SLQIS-PPNS QPSST--WQR
Cit-sin_KAN2 ---------- --MELFPA-- --Q----PDL SLQIS-PPNS KPSST--WSS
Vit-vin_KAN2 ---------- --MELFPA-- --Q----PDL SLQIS-PPNS KPSSG--W--
The-cacao_KAN2 ---------- --MELFPA-- --Q----PDL SLQIS-PPNS KPSST--W--
Med-tru_KAN2 ---------- --MELFPA-- --Q----PDL SLQIS-PPNT KQTTTSNWKR
Glycine_KAN2 ---------- --MELFPA-- --Q----PDL SLQIS-PPNA KPTSS--W--
Vig-rad_KAN2 ---------- --MELFPA-- --Q----PDL SLQIS-PPNA KPTST--W--
Vig-ang_KAN2 ---------- --MELFPA-- --Q----PDL SLQIS-PPNA KPTST--W--
Vit-vin_KAN2-variantX4 ---------- --MELFPA-- --Q----PDL SLQIS-PPNS KPSSG--W--
Pop-trichocarpa ---------- --MELFPA-- --Q----PDL SLQIS-PPNS KPTST--W--
Ath_KAN3 ---------- --MELFPS-- --Q----PDL YLKIS-RRRE EEQEK-----
Bra-napus_KAN3 ---------- --MELFPS-- --Q----PDL YLKIN-RRRE EQEEE-----
Bra-nap_KAN3 ---------- --MELFPS-- --Q----PDL YLKIN-RRRE EQEEE-----
Bra-nap_KAN3 ---------- --MELFPS-- --Q----PDL YLKIN-RRRE EQEEE-----
Cam-sat_KAN3 ---------- --MELFPS-- --Q----PDL YLKIS-RRRE EEKEN-----
Raph-sat_KAN3 ---------- --MELFPS-- --Q----PDL YLKIN-RRRE EQEEE-----
Vig-ang_KAN2 ---------- --MELFPA-- --Q----PDL SLQIS-PPNA KPTST--W--
Med-tru_KAN2 ---------- --MELFPA-- --Q----PDL SLQIS-PPNT KQTTTSNWKR
Vig-rad_KAN2 ---------- --MELFPA-- --Q----PDL SLQIS-PPNA KPTST--W--
Glyc_KAN2 ---------- --MELFPA-- --Q----PDL SLQIS-PPNA KPTSS--W--
Vit-vin_KAN2 ---------- --MELFPA-- --Q----PDL SLQIS-PPNS KPSSG--W--
Theo-cac_KAN2 ---------- --MELFPA-- --Q----PDL SLQIS-PPNS KPSST--W--
Pop-tri_hypothetical.protein ---------- --MELFPA-- --Q----PDL SLQIS-PPNS KPTST--W--
Ric-com_KAN2 ---------- --MELFPA-- --Q----PDL SLQIS-PPNS KPTST--W--
Ath_KAN4 ---MMMLESR NSMRASNS-- --V----PDL SLQIS-LPNY HAGKP-----
Ric-com_KAN4 ----MFSSSN LMMRTASS-- --L----PDL SLQIS-PPLA SDCEA-----
Pop-tri_KAN4 --MFSSSKHI IMRSVSPF-- -------PDL SLQIS-PPSV EAKET-----
Nel-nuc_KAN4 ---------- -MRTTLPF-- -------PDL SLQIS-PPTI PDCEG-----
The-cac_KAN4 ----MFTSSN NIMRITPL-- -------PDL SLQIS-PPSV SDCKA-----
Vit-vin_KAN4 ---MFFSSNP IMGTTSPL-- -------PDL SLQIS-PPSI SDCEV-----
Glycine_KAN4 MFTNSQTVMQ TLLSSPSA-- --E----PDL SLNIS-PPSI SDSEA-----
Sol-lyc_KAN4 ---------M TKDTLLST-- --V----PDL SLQISLPSCI LRSDQ-----
Med_tru_KAN4 --------MH TLFSPLLE-- -------PDL SLNISLPSNI SDSEP-----
Vit-vin_KAN4 ---MFFSSNP IMGTTSPL-- -------PDL SLQIS-PPSI SDCEV-----
Pop-tri_KAN4 ---------- MMRAASPF-- -------PYL SLQIS-PPAV LEAKE-----
Sol_tub_KAN4 ---------M TTDTVLST-- --V----PDL SLQISLPSSI LKSNQ-----
Pru-per_KAN4 ---------- --MYLMNPIK RTSTSPLPDL SLQIS-PPSA ADNQY-----
Amb-tri_KAN4 ---------- --MDMRAG-- --HVF--PDL SLQIS-PPSQ MDLEM-----
Fra-vesca_KAN4 MMYLSSNSNP IMRSTSSP-- --L----PDL SLQIS-PPSD HYEIA-----
Mus-acu_KAN4 ---------- -MATIF---- -------PDL SLHIS-PPAI SVDGS-----
Glyc_KAN4 ---------- ---------- ---------- ---------- ----------
Glyc-KAN4 -MYTTWHTGM ATFAPLPE-- -------PDL SLNIS-PPFI SDSDA-----
Cam-sat_KAN4 --MIMLESRN SIMRASNS-- --V----PDL SLQISLLPNS QAGKP-----
Bra-rapa_KAN4 ---MIMFESR SSVRGSNS-- --V----PDL SLQIS-LPNS HAEKP-----
Bra-nap_KAN4 ---MIMFESR SSVRGSNS-- --V----PDL SLQIS-LPNS HAEKP-----

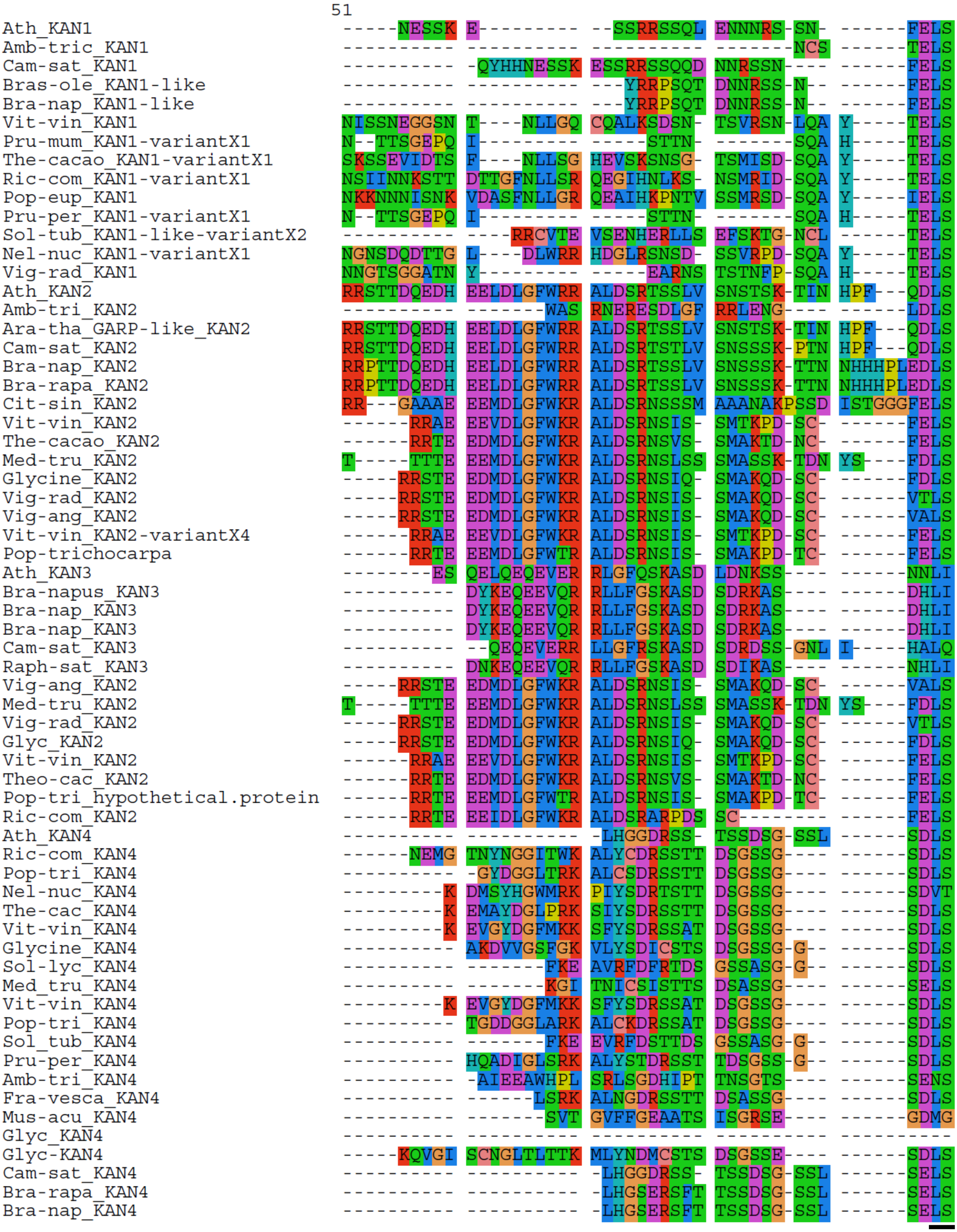

51
Ath_KAN1 -----NESSK E--------- --SSRRSSQL ENNNRS-SN- ------FELS
Amb-tric_KAN1 ---------- ---------- ---------- -------NCS ------TELS
Cam-sat_KAN1 ---------- -QYHHNESSK ESSRRSSQQD NNRSSN---- ------FELS
Bras-ole_KAN1-like ---------- ---------- ---YRRPSQT DNNRSS-N-- ------FELS
Bra-nap_KAN1-like ---------- ---------- ---YRRPSQT DNNRSS-N-- ------FELS
Vit-vin_KAN1 NISSNEGGSN T----NLLGQ CQALKSDSN- TSVRSN-LQA Y-----TELS
Pru-mum_KAN1-variantX1 N--TTSGEPQ I--------- -----STTN- -------SQA H-----TELS
The-cacao_KAN1-variantX1 SKSSEVIDTS F----NLLSG HEVSKSNSG- TSMISD-SQA Y-----TELS
Ric-com_KAN1-variantX1 NSIINNKSTT DTTGFNLLSR QEGIHNLKS- NSMRID-SQA Y-----TELS
Pop-eup_KAN1 NKKNNNISNK VDASFNLLGR QEAIHKPNTV SSMRSD-SQA Y-----IELS
Pru-per_KAN1-variantX1 N--TTSGEPQ I--------- -----STTN- -------SQA H-----TELS
Sol-tub_KAN1-like-variantX2 ---------- ----RRCVTE VSENHERLLS EFSKTG-NCL ------TELS
Nel-nuc_KAN1-variantX1 NGNSDQDTTG L----DLWRR HDGLRSNSD- SSVRPD-SQA Y-----TELS
Vig-rad_KAN1 NNGTSGGATN Y--------- -----EARNS TSTNFP-SQA H-----TELS
Ath_KAN2 RRSTTDQEDH EELDLGFWRR ALDSRTSSLV SNSTSK-TIN HPF---QDLS
Amb-tri_KAN2 ---------- -------WAS RNERESDLGF RRLENG---- ------LDLS
Ara-tha_GARP-like_KAN2 RRSTTDQEDH EELDLGFWRR ALDSRTSSLV SNSTSK-TIN HPF---QDLS
Cam-sat_KAN2 RRSTTDQEDH EELDLGFWRR ALDSRTSTLV SNSSSK-PTN HPF---QDLS
Bra-nap_KAN2 RRPTTDQEDH EELDLGFWRR ALDSRTSSLV SNSSSK-TTN NHHHPLEDLS
Bra-rapa_KAN2 RRPTTDQEDH EELDLGFWRR ALDSRTSSLV SNSSSK-TTN NHHHPLEDLS
Cit-sin_KAN2 RR---GAAAE EEMDLGFWKR ALDSRNSSSM AAANAKPSSD ISTGGGFELS
Vit-vin_KAN2 ------RRAE EEVDLGFWKR ALDSRNSIS- SMTKPD-SC- ------FELS
The-cacao_KAN2 ------RRTE EDMDLGFWKR ALDSRNSVS- SMAKTD-NC- ------FELS
Med-tru_KAN2 T-----TTTE EEMDLGFWKR ALDSRNSLSS SMASSK-TDN YS----FDLS
Glycine_KAN2 -----RRSTE EDMDLGFWKR ALDSRNSIQ- SMAKQD-SC- ------FDLS
Vig-rad_KAN2 -----RRSTE EDMDLGFWKR ALDSRNSIS- SMAKQD-SC- ------VTLS
Vig-ang_KAN2 -----RRSTE EDMDLGFWKR ALDSRNSIS- SMAKQD-SC- ------VALS
Vit-vin_KAN2-variantX4 ------RRAE EEVDLGFWKR ALDSRNSIS- SMTKPD-SC- ------FELS
Pop-trichocarpa ------RRTE EEMDLGFWTR ALDSRNSIS- SMAKPD-TC- ------FELS
Ath_KAN3 --------ES QELQEQEVER RLGFQSKASD LDNKSS---- ------NNLI
Bra-napus_KAN3 ---------- DYKEQEEVQR RLLFGSKASD SDRKAS---- ------DHLI
Bra-nap_KAN3 ---------- DYKEQEEVQR RLLFGSKASD SDRKAS---- ------DHLI
Bra-nap_KAN3 ---------- DYKEQEEVQR RLLFGSKASD SDRKAS---- ------DHLI
Cam-sat_KAN3 ---------- --QEQEVERR LLGFRSKASD SDRDSS-GNL I-----HALQ
Raph-sat_KAN3 ---------- DNKEQEEVQR RLLFGSKASD SDIKAS---- ------NHLI
Vig-ang_KAN2 -----RRSTE EDMDLGFWKR ALDSRNSIS- SMAKQD-SC- ------VALS
Med-tru_KAN2 T-----TTTE EEMDLGFWKR ALDSRNSLSS SMASSK-TDN YS----FDLS
Vig-rad_KAN2 -----RRSTE EDMDLGFWKR ALDSRNSIS- SMAKQD-SC- ------VTLS
Glyc_KAN2 -----RRSTE EDMDLGFWKR ALDSRNSIQ- SMAKQD-SC- ------FDLS
Vit-vin_KAN2 ------RRAE EEVDLGFWKR ALDSRNSIS- SMTKPD-SC- ------FELS
Theo-cac_KAN2 ------RRTE EDMDLGFWKR ALDSRNSVS- SMAKTD-NC- ------FELS
Pop-tri_hypothetical.protein ------RRTE EEMDLGFWTR ALDSRNSIS- SMAKPD-TC- ------FELS
Ric-com_KAN2 ------RRTE EEIDLGFWKR ALDSRARPDS SC-------- ------FELS
Ath_KAN4 ---------- ---------- -LHGGDRSS- TSSDSG-SSL ------SDLS
Ric-com_KAN4 ------NEMG TNYNGGITWK ALYCDRSSTT DSGSSG---- ------SDLS
Pop-tri_KAN4 ---------- -GYDGGLTRK ALCSDRSSTT DSGSSG---- ------SDLS
Nel-nuc_KAN4 ---------K DMSYHGWMRK PIYSDRTSTT DSGSSG---- ------SDVT
The-cac_KAN4 ---------K EMAYDGLPRK SIYSDRSSTT DSGSSG---- ------SDLS
Vit-vin_KAN4 ---------K EVGYDGFMKK SFYSDRSSAT DSGSSG---- ------SDLS
Glycine_KAN4 ---------- AKDVVGSFGK VLYSDICSTS DSGSSG-G-- ------SDLS
Sol-lyc_KAN4 ---------- -------FKE AVRFDFRTDS GSSASG-G-- ------SDLS
Med_tru_KAN4 ---------- -------KGI TNICSISTTS DSASSG---- ------SELS
Vit-vin_KAN4 ---------K EVGYDGFMKK SFYSDRSSAT DSGSSG---- ------SDLS
Pop-tri_KAN4 ---------- TGDDGGLARK ALCKDRSSAT DSGSSG---- ------SDLS
Sol_tub_KAN4 ---------- -------FKE EVRFDSTTDS GSSASG-G-- ------SDLS
Pru-per_KAN4 ---------- HQADIGLSRK ALYSTDRSST TDSGSS-G-- ------SDLS
Amb-tri_KAN4 ---------- -AIEEAWHPL SRLSGDHIPT TNSGTS---- ------SENS
Fra-vesca_KAN4 ---------- ------LSRK ALNGDRSSTT DSASSG---- ------SDLS
Mus-acu_KAN4 ---------- -------SVT GVFFGEAATS ISGRSE---- ------GDMG
Glyc_KAN4 ---------- ---------- ---------- ---------- ----------
Glyc-KAN4 -----KQVGI SCNGLTLTTK MLYNDMCSTS DSGSSE---- ------SDLS
Cam-sat_KAN4 ---------- ---------- -LHGGDRSS- TSSDSG-SSL ------SELS
Bra-rapa_KAN4 ---------- ---------- -LHGSERSFT TSSDSG-SSL ------SELS
Bra-nap_KAN4 ---------- ---------- -LHGSERSFT TSSDSG-SSL ------SELS

```
                                101
Ath_KAN1                        LSH------- ---------- ----HNHPTA ---------- -----RIFHC
Amb-tric_KAN1                   LANPSAPQEN Q--------- ---------- ---------- -----EPHFL
Cam-sat_KAN1                    LSHHNHPTA- ---------- ---------- ---------- -----RIFHC
Bras-ole_KAN1-like              LSHHHNPSSN PTT------- ---------- ---------- -----RIIHC
Bra-nap_KAN1-like               LSHHHNPSSN PTT------- ---------- ---------- -----RIIHS
Vit-vin_KAN1                    LAH--AANVM DEDGRCSRNF ---TVGADQE -----QLH-- -----NPYH-
Pru-mum_KAN1-variantX1          LG-------- -------RRF TGGAH---EE -----PPQ-- -----KPYH-
The-cacao_KAN1-variantX1        LARPHPANVM EQERQCRRN- ---STGGAEQ -----PSH-- -----TPLH-
Ric-com_KAN1-variantX1          LAHPATTNVV DDETRIRRNF TASSTATAQE -----PPH-- -----NPYQ-
Pop-eup_KAN1                    LAHP-TTGLD EESRRSRRNL --SSSRAVEL -----PQL-- -----NPYH-
Pru-per_KAN1-variantX1          LG-------- -------RRF TGGAH---EE -----PPQ-- -----NPYH-
Sol-tub_KAN1-like-variantX2     LAHPTNITTT TTTVINDDMT TRRGFLDHLP R--------- -----NPYYI
Nel-nuc_KAN1-variantX1          LAN--PRNGF EAESQWRRSF TRGGAEEDEL -----RHE-- -----QPTD-
Vig-rad_KAN1                    LG-------- -------RNF SGGGRGGIEE -----PPPHN SNTSINPYY-
Ath_KAN2                        LSN------- ---------- ---------- ---------- -----ISHHQ
Amb-tri_KAN2                    LER------- ---------- ---------- ---------- ----------
Ara-tha_GARP-like_KAN2          LSN------- ---------- ---------- ---------- -----ISHHQ
Cam-sat_KAN2                    LSN------- ---------- ---------- ---------- -----NSHHH
Bra-nap_KAN2                    LS-------- ---------- ---------- ---------- -----NNSHR
Bra-rapa_KAN2                   LSN------- ---------- ---------- ---------- -----NSHR-
Cit-sin_KAN2                    LSNPARVSSS SVLSESLSSN NNLNHHHQFH LLQNNTTTTT NCNGNNIFH-
Vit-vin_KAN2                    LSNPRATSES NNSNHFHLLQ NGNT------ ---------- -----NLLH-
The-cacao_KAN2                  LSNPRVSESN SNHLHLLQNG GANCNG---- ---------- -----NLFH-
Med-tru_KAN2                    LSNPTKPLDS NNNTSNNLIH HHHFQNSGNN NAN------- -----NPYQS
Glycine_KAN2                    LSNPKASDNN NNHHSNTTTS NLIHHHHFQN ---GATPTNA TTTTTNPFQL
Vig-rad_KAN2                    LSNPKASSDN SNSSNLIHHF QNGANNATT- ---------- -----NPFQ-
Vig-ang_KAN2                    LSNPKASSDN SNSSNLIHRF QHGANNATT- ---------- -----NPFQ-
Vit-vin_KAN2-variantX4          LSNPRATSES NNSNHFHLLQ NGNT------ ---------- -----NLLH-
Pop-trichocarpa                 LSNPKVSEPN SNHFHNILQN SNNCNGN--- ---------- -----NLFH-
Ath_KAN3                        HT-------- ---------- ---------- ---------- -----LQFTS
Bra-napus_KAN3                  HT-------- ---------- ---------- ---------- -----LQFTS
Bra-nap_KAN3                    HT-------- ---------- ---------- ---------- -----LQFTS
Bra-nap_KAN3                    HT-------- ---------- ---------- ---------- -----LQFTS
Cam-sat_KAN3                    FTSSKS---- ---------- ---------- ---------- -----EPIKT
Raph-sat_KAN3                   QA-------- ---------- ---------- ---------- -----LQFTS
Vig-ang_KAN2                    LSNPKASSDN SNSSNLIHRF QHGANNATT- ---------- -----NPFQ-
Med-tru_KAN2                    LSNPTKPLDS NNNTSNNLIH HHHFQNSGNN NAN------- -----NPYQS
Vig-rad_KAN2                    LSNPKASSDN SNSSNLIHHF QNGANNATT- ---------- -----NPFQ-
Glyc_KAN2                       LSNPKASDNN NNHHSNTTTS NLIHHHHFQN ---GATPTNA TTTTTNPFQL
Vit-vin_KAN2                    LSNPRATSES NNSNHFHLLQ NGNT------ ---------- -----NLLH-
Theo-cac_KAN2                   LSNPRVSESN SNHLHLLQNG GANCNG---- ---------- -----NLFH-
Pop-tri_hypothetical.protein    LSNPKVSEPN SNHFHNILQN SNNCNGN--- ---------- -----NLFH-
Ric-com_KAN2                    LSNPRISESS KHFHLLQNSN NYNGT----- ---------- -----NLFN-
Ath_KAN4                        HEN------- ---------- ---------- ---------- -----NFFN-
Ric-com_KAN4                    HEN------- ---------- ---------- ---------- -----GTLN-
Pop-tri_KAN4                    HEN------- ---------- ---------- ---------- -----GFLNQ
Nel-nuc_KAN4                    HEQ------- ---------- ---------- ---------- -----GFLHL
The-cac_KAN4                    HEN------- ---------- ---------- ---------- -----GY---
Vit-vin_KAN4                    HEN------- ---------- ---------- ---------- -----GFFSP
Glycine_KAN4                    HE-------- ---------- ---------- ---------- -------FH-
Sol-lyc_KAN4                    HENT------ ---------- ---------- ---------- -----VFLHP
Med_tru_KAN4                    HE-------- ---------- ---------- ---------- -----NPFIY
Vit-vin_KAN4                    HEN------- ---------- ---------- ---------- -----GFFSP
Pop-tri_KAN4                    HEN------- ---------- ---------- ---------- -----GLFN-
Sol_tub_KAN4                    HENT------ ---------- ---------- ---------- -----GFFHP
Pru-per_KAN4                    HEN------- ---------- ---------- ---------- -----GFYHL
Amb-tri_KAN4                    QE-------- ---------- ---------- ---------- -----SPFLL
Fra-vesca_KAN4                  HEN------- ---------- ---------- ---------- -----VLYHL
Mus-acu_KAN4                    HDQ------- ---------- ---------- ---------- -----AFLHH
Glyc_KAN4                       ---------- ---------- ---------- ---------- ----------
Glyc-KAN4                       HEN------- ---------- ---------- ---------- -----GFFLM
Cam-sat_KAN4                    HEN------- ---------- ---------- ---------- -----NFFNK
Bra-rapa_KAN4                   HEN------- ---------- ---------- ---------- -----SFLKK
Bra-nap_KAN4                    HEN------- ---------- ---------- ---------- -----SFLKK
```

```
                              151
Ath_KAN1                      PDR---RTLN LPHQQH---- ---------- ----YNNPII ---------N
Amb-tric_KAN1                 SLP----HFQ NHHQNH---- ---------- ---------- ---------P
Cam-sat_KAN1                  PDR---RNLN LPHHQQH--- ---------- ----YNNPII NGG------S
Bras-ole_KAN1-like            PDP---RNLN LPHHQH---- ---------- ----YNNPIL NG-------G
Bra-nap_KAN1-like             PDP---RNLN LPHHQH---- ---------- ----YNNPIL NG-------G
Vit-vin_KAN1                  QNR----HHH LSHHPN---- ---------- ------NNLN H--------I
Pru-mum_KAN1-variantX1        HHQ----QQS LPLYR----- ---------- ----SSNHLS H--------I
The-cacao_KAN1-variantX1      LT-----HRH LHP------- ---------- ----TNTHLN H--------I
Ric-com_KAN1-variantX1        QQS----HNH LHQHHH---- ---------- ----NTHHLN H--------L
Pop-eup_KAN1                  QHS----HNH QQQQQHLHHS NS-------- ----TTHHLN H--------I
Pru-per_KAN1-variantX1        HHQ----QQS LPLYR----- ---------- ----SSNHLS H--------I
Sol-tub_KAN1-like-variantX2   NHH----HQH HHQSQN---- ---------- ----YSHMVQ PM-------N
Nel-nuc_KAN1-variantX1        HYS----QHH HLRHP----- ---------- ----NSNRLN H--------L
Vig-rad_KAN1                  QTQ----QPH FHHLHHHSNT GNASKPATAT TTTSSSNHLS H--------I
Ath_KAN2                      QQQ----QHH HPQLLPNCN- ---------- ----SSNILT SFQFPTQQQQ
Amb-tri_KAN2                  ---------- ---------- ---------- ---------- ---------E
Ara-tha_GARP-like_KAN2        QQQ----QHH HPQLLPNCN- ---------- ----SSNILT SFQFPTQQQQ
Cam-sat_KAN2                  QQH----QHH QPHLVPNCN- ---------- ----GSNILT SFQFPTQQQQ
Bra-nap_KAN2                  HHQQIFTSFQ FPTQQ----- ---------- ---------- ---------Q
Bra-rapa_KAN2                 HHQQIFTSFQ FPTQQ----- ---------- ---------- ---------Q
Cit-sin_KAN2                  SFHHQQNHSQ FPTHHH---- ---------- ------LHQN QHHHPVLYHQ
Vit-vin_KAN2                  SFQ----QSQ YPHHHH---- ---------- ----HQHQLH HQ-------L
The-cacao_KAN2                TYH----QNQ FPHRHNQ--- ---------- ----LQHPVL YQ-------H
Med-tru_KAN2                  FHQNNYFQHP HPQPLFQHHQ ---------- ---------- ---------P
Glycine_KAN2                  PFQ----KNH YFHQQQQPL- ---------- ---------- ---------F
Vig-rad_KAN2                  PFQ----QNH YFHQPL---- ---------- ---------- ---------F
Vig-ang_KAN2                  PFQ----QNH YFHQPL---- ---------- ---------- ---------F
Vit-vin_KAN2-variantX4        SFQ----QSQ YPHHHH---- ---------- ----HQHQLH HQ-------L
Pop-trichocarpa               SFQ----QNQ YTHHHH---- ---------- ----QQNHLH NHHPVLF--Q
Ath_KAN3                      NNE----ATK INS------- ---------- ---------- ---------N
Bra-napus_KAN3                NNE----PTK IDH------- ---------- ---------- ---------N
Bra-nap_KAN3                  NNE----PTK IDH------- ---------- ---------- ---------N
Bra-nap_KAN3                  NNE----PTK IDH------- ---------- ---------- ---------N
Cam-sat_KAN3                  GNN------- ---------- ---------- ---------- ----------
Raph-sat_KAN3                 DNK----PSK IDH------- ---------- ---------- ---------N
Vig-ang_KAN2                  PFQ----QNH YFHQPL---- ---------- ---------- ---------F
Med-tru_KAN2                  FHQNNYFQHP HPQPLFQHHQ ---------- ---------- ---------P
Vig-rad_KAN2                  PFQ----QNH YFHQPL---- ---------- ---------- ---------F
Glyc_KAN2                     PFQ----KNH YFHQQQQPL- ---------- ---------- ---------F
Vit-vin_KAN2                  SFQ----QSQ YPHHHH---- ---------- ----HQHQLH HQ-------L
Theo-cac_KAN2                 TYH----QNQ FPHRH----- ---------- ---------N QLQHPVLYQH
Pop-tri_hypothetical.protein  SFQ----QNQ YTHHHH---- ---------- ----QQNHLH NHHPVLF--Q
Ric-com_KAN2                  SFQ----QNQ YPHHQQPKS- ---------- ----HLLHQH SPHFQQQQHH
Ath_KAN4                      ---------- ---------- ---------- ---------- ----------
Ric-com_KAN4                  -------QER SYN------- ---------- ---------- ---------N
Pop-tri_KAN4                  ERS------- ---------- ---------- ---------- ---------Y
Nel-nuc_KAN4                  ERA------- ---------- ---------- ---------- ---------Y
The-cac_KAN4                  ---------- ---------- ---------- ---------- ---------S
Vit-vin_KAN4                  ESG------- ---------- ---------- ---------- ---------F
Glycine_KAN4                  -------NLG HHHHRE---- ---------- ---------- ---------P
Sol-lyc_KAN4                  EIKKAFNLPL IPTFE----- ---------- ---------- ----------
Med_tru_KAN4                  PHQ----RDP TLRLG----- ---------- ---------- ---------F
Vit-vin_KAN4                  ESG------- ---------- ---------- ---------- ---------F
Pop-tri_KAN4                  -------QDR IAYNL----- ---------- ---------- ----------
Sol_tub_KAN4                  EIKKVFNLPF IPSFE----- ---------- ---------- ----------
Pru-per_KAN4                  ERS-----TG Y--------- ---------- ---------- ---------N
Amb-tri_KAN4                  HHR------- ---------- ---------- ---------- --------LM
Fra-vesca_KAN4                DQR----TSY NPAAA----- ---------- ---------- ----------
Mus-acu_KAN4                  DQG------- ---------- ---------- ---------- ---------R
Glyc_KAN4                     ---------- ---------- ---------- ---------- ----------
Glyc-KAN4                     DTA------- ---------- ---------- ---------- ---------Y
Cam-sat_KAN4                  PLL----SLG FDHH------ ---------- ---------- ----------
Bra-rapa_KAN4                 HLL----SLG FDHHH----- ---------- ---------- ----------
Bra-nap_KAN4                  HLL----SLG FDHHH----- ---------- ---------- ----------
```

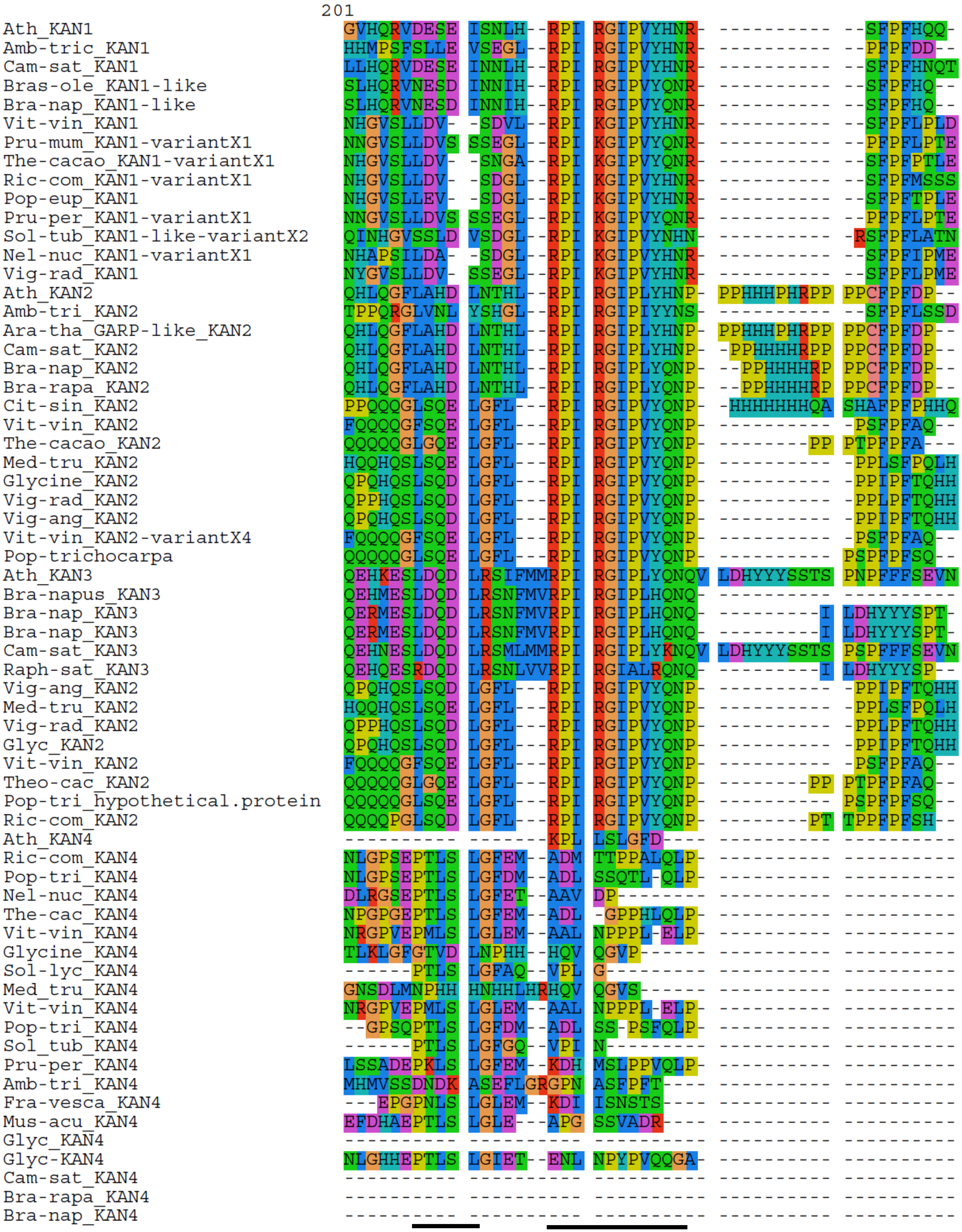
201
Ath_KAN1 GVHQRVDESE ISNLH--RPI RGIPVYHNR- ---------- --SFPFHQQ-
Amb-tric_KAN1 HHMPSFSLLE VSEGL--RPI RGIPVYHNR- ---------- --PFPFDD--
Cam-sat_KAN1 LLHQRVDESE INNLH--RPI RGIPVYHNR- ---------- --SFPFHNQT
Bras-ole_KAN1-like SLHQRVNESD INNIH--RPI RGIPVYQNR- ---------- --SFPFHQ--
Bra-nap_KAN1-like SLHQRVNESD INNIH--RPI RGIPVYQNR- ---------- --SFPFHQ--
Vit-vin_KAN1 NHGVSLLDV- -SDVL--RPI KGIPVYHNR- ---------- --SFPFLPLD
Pru-mum_KAN1-variantX1 NNGVSLLDVS SSEGL--RPI KGIPVYQNR- ---------- --PFPFLPTE
The-cacao_KAN1-variantX1 NHGVSLLDV- -SNGA--RPI KGIPVYQNR- ---------- --SFPFPTLE
Ric-com_KAN1-variantX1 NHGVSLLDV- -SDGL--RPI KGIPVYHNR- ---------- --SFPFMSSS
Pop-eup_KAN1 NHGVSLLEV- -SDGL--RPI KGIPVYHNR- ---------- --SFPFTPLE
Pru-per_KAN1-variantX1 NNGVSLLDVS SSEGL--RPI KGIPVYQNR- ---------- --PFPFLPTE
Sol-tub_KAN1-like-variantX2 QINHGVSSLD VSDGL--RPI KGIPVYNHN- ---------- -RSFPFLATN
Nel-nuc_KAN1-variantX1 NHAPSILDA- -SDGL--RPI KGIPVYHNR- ---------- --SFPFIPME
Vig-rad_KAN1 NYGVSLLDV- SSEGL--RPI KGIPVYHNR- ---------- --SFPFLPME
Ath_KAN2 QHLQGFLAHD LNTHL--RPI RGIPLYHNP- PPHHHPHRPP PPCFPFDP--
Amb-tri_KAN2 TPPQRGLVNL YSHGL--RPI RGIPIYYNS- ---------- --SFPFLSSD
Ara-tha_GARP-like_KAN2 QHLQGFLAHD LNTHL--RPI RGIPLYHNP- PPHHHPHRPP PPCFPFDP--
Cam-sat_KAN2 QHLQGFLAHD LNTHL--RPI RGIPLYHNP- -PPHHHHRPP PPCFPFDP--
Bra-nap_KAN2 QHLQGFLAHD LNTHL--RPI RGIPLYQNP- --PPHHHHRP PPCFPFDP--
Bra-rapa_KAN2 QHLQGFLAHD LNTHL--RPI RGIPLYQNP- --PPHHHHRP PPCFPFDP--
Cit-sin_KAN2 PPQQQGLSQE LGFL---RPI RGIPVYQNP- -HHHHHHHQA SHAFPFPHHQ
Vit-vin_KAN2 FQQQQGFSQE LGFL---RPI RGIPVYQNP- ---------- -PSFPFAQ--
The-cacao_KAN2 QQQQQGLGQE LGFL---RPI RGIPVYQNP- --------PP PTPFPFA---
Med-tru_KAN2 HQQHQSLSQE LGFL---RPI RGIPVYQNP- ---------- -PPLSFPQLH
Glycine_KAN2 QPQHQSLSQD LGFL---RPI RGIPVYQNP- ---------- -PPIPFTQHH
Vig-rad_KAN2 QPPHQSLSQD LGFL---RPI RGIPVYQNP- ---------- -PPLPFTQHH
Vig-ang_KAN2 QPQHQSLSQD LGFL---RPI RGIPVYQNP- ---------- -PPIPFTQHH
Vit-vin_KAN2-variantX4 FQQQQGFSQE LGFL---RPI RGIPVYQNP- ---------- -PSFPFAQ--
Pop-trichocarpa QQQQQGLSQE LGFL---RPI RGIPVYQNP- ---------- PSPFPFSQ--
Ath_KAN3 QEHKESLDQD LRSIFMMRPI RGIPLYQNQV LDHYYYSSTS PNPFFFSEVN
Bra-napus_KAN3 QEHMESLDQD LRSNFMVRPI RGIPLHQNQ- ---------- ----------
Bra-nap_KAN3 QERMESLDQD LRSNFMVRPI RGIPLHQNQ- ---------I LDHYYYSPT-
Bra-nap_KAN3 QERMESLDQD LRSNFMVRPI RGIPLHQNQ- ---------I LDHYYYSPT-
Cam-sat_KAN3 QEHNESLDQD LRSMLMMRPI RGIPLYKNQV LDHYYYSSTS PSPFFFSEVN
Raph-sat_KAN3 QEHQESRDQD LRSNLVVRPI RGIALRQNQ- ---------I LDHYYYSP--
Vig-ang_KAN2 QPQHQSLSQD LGFL---RPI RGIPVYQNP- ---------- -PPIPFTQHH
Med-tru_KAN2 HQQHQSLSQE LGFL---RPI RGIPVYQNP- ---------- -PPLSFPQLH
Vig-rad_KAN2 QPPHQSLSQD LGFL---RPI RGIPVYQNP- ---------- -PPLPFTQHH
Glyc_KAN2 QPQHQSLSQD LGFL---RPI RGIPVYQNP- ---------- -PPIPFTQHH
Vit-vin_KAN2 FQQQQGFSQE LGFL---RPI RGIPVYQNP- ---------- -PSFPFAQ--
Theo-cac_KAN2 QQQQQGLGQE LGFL---RPI RGIPVYQNP- --------PP PTPFPFAQ--
Pop-tri_hypothetical.protein QQQQQGLSQE LGFL---RPI RGIPVYQNP- ---------- PSPFPFSQ--
Ric-com_KAN2 QQQQPGLSQD LGFL---RPI RGIPVYQNP- --------PT TPPFPFSH--
Ath_KAN4 ---------- -------KPL LSLGFD---- ---------- ----------
Ric-com_KAN4 NLGPSEPTLS LGFEM--ADM TTPPALQLP- ---------- ----------
Pop-tri_KAN4 NLGPSEPTLS LGFDM--ADL SSQTL-QLP- ---------- ----------
Nel-nuc_KAN4 DLRGSEPTLS LGFET--AAV DP-------- ---------- ----------
The-cac_KAN4 NPGPGEPTLS LGFEM--ADL -GPPHLQLP- ---------- ----------
Vit-vin_KAN4 NRGPVEPMLS LGLEM--AAL NPPPL-ELP- ---------- ----------
Glycine_KAN4 TLKLGFGTVD LNPHH--HQV QGVP------ ---------- ----------
Sol-lyc_KAN4 ------PTLS LGFAQ--VPL G--------- ---------- ----------
Med_tru_KAN4 GNSDLMNPHH HNHHLHRHQV QGVS------ ---------- ----------
Vit-vin_KAN4 NRGPVEPMLS LGLEM--AAL NPPPL-ELP- ---------- ----------
Pop-tri_KAN4 --GPSQPTLS LGFDM--ADL SS-PSFQLP- ---------- ----------
Sol_tub_KAN4 ------PTLS LGFGQ--VPI N--------- ---------- ----------
Pru-per_KAN4 LSSADEPKLS LGFEM--KDH MSLPPVQLP- ---------- ----------
Amb-tri_KAN4 MHMVSSDNDK ASEFLGRGPN ASFPFT---- ---------- ----------
Fra-vesca_KAN4 ---EPGPNLS LGLEM--KDI ISNSTS---- ---------- ----------
Mus-acu_KAN4 EFDHAEPTLS LGLE---APG SSVADR---- ---------- ----------
Glyc_KAN4 ---------- ---------- ---------- ---------- ----------
Glyc-KAN4 NLGHHEPTLS LGIET--ENL NPYPVQQGA- ---------- ----------
Cam-sat_KAN4 ---------- ---------- ---------- ---------- ----------
Bra-rapa_KAN4 ---------- ---------- ---------- ---------- ----------
Bra-nap_KAN4 ---------- ---------- ---------- ---------- ----------

```
                                    251
Ath_KAN1                            ---------- ---------- -------NSS LPS----LGG GDMDQISILN
Amb-tric_KAN1                       ---------- ---------- PKMGSRLYHL SYPSTSSSSL SNSCSSPNPC
Cam-sat_KAN1                        ---------- ---------- ------SPSS SLPSLGGGGG GDLDQISILN
Bras-ole_KAN1-like                  ---------- ---------- -------QTS LPS---LGGG GDLDQISILN
Bra-nap_KAN1-like                   ---------- ---------- -------QTS LPS---LGGG GDLDQISILN
Vit-vin_KAN1                        -------HSR E-------KD SRMN--LYQL SYPSWSSSLA PSSSCSSLCS
Pru-mum_KAN1-variantX1              -----NMIPR DHP-----KD SKVC--FYQR PPPSYN---- LPSLPSASCS
The-cacao_KAN1-variantX1            ------NLSR E-------RD PKMC--FYQI PYP------- ----SSSLYS
Ric-com_KAN1-variantX1              EQQQQLQSSR ENSNNNISRD PKMC--FYQM PYH------- PSSSSSSLCS
Pop-eup_KAN1                        ------HSSR EN------KD PKMC--FYQM PYP------- SSSFFSPVNP
Pru-per_KAN1-variantX1              -----NMIPR DHP-----KD SKMC--FYQR PPPSYN---- LPSLPSASCS
Sol-tub_KAN1-like-variantX2         ---------- ---------- IDKEKDPKMC FYPSSSPSSS SPSSPSPYFN
Nel-nuc_KAN1-variantX1              -------RSR E-------KD GKMC--FYQM SYPSWSSSLC SSSSPSSVCS
Vig-rad_KAN1                        -----HSGDK DH-------H AKMC--LYHH MPTYPS---- LSSSCSPLCS
Ath_KAN2                            ---------- ---------- -------SSL IPS------- SSTSSPALTG
Amb-tri_KAN2                        ---------- ---------- ------HFGD LHS--SSSLS SSTSCPSLSS
Ara-tha_GARP-like_KAN2              ---------- ---------- -------SSL IPS------- SSTSSPALTG
Cam-sat_KAN2                        ---------- ---------- -------SSL IPS------- SSPSPPSLTG
Bra-nap_KAN2                        ---------- ---------- -------SSL IPS------- SSPTPTGINN
Bra-rapa_KAN2                       ---------- ---------- -------SSL IPS------- SSPTPTGINN
Cit-sin_KAN2                        ---------- ---------- -------QQP LDS------- -PPPPSLVAN
Vit-vin_KAN2                        ---------- ---------- --------QP LDS-----SI ASSTTTNTAA
The-cacao_KAN2                      ---------- ---------- -------QQP LDS------S LASSPSSLVT
Med-tru_KAN2                        ---------- ---------- -----NHNHN LNHLHILDGT TTTTPSSISN
Glycine_KAN2                        ---------- ---------- ----HHHHLP LEA------- STTTPSIISN
Vig-rad_KAN2                        ---------- ---------- ----HHHHLP LEP------- STTTPSSIIS
Vig-ang_KAN2                        ---------- ---------- ---HHHHHLP LEP------- STTAPSSIIS
Vit-vin_KAN2-variantX4              ---------- ---------- --------QP LDS-----SI ASSTTTNTAA
Pop-trichocarpa                     ---------- ---------- --------QT FDN------- TCSQASSLAA
Ath_KAN3                            ---------- ---------- ---------- ---------- GQHASRRLIT
Bra-napus_KAN3                      ---------- ---------- ---ILDHYYY SPT------- ----------
Bra-nap_KAN3                        ---------- ---------- ---------- ---------- ----------
Bra-nap_KAN3                        ---------- ---------- ---------- ---------- ----------
Cam-sat_KAN3                        ---------- ---------- ---------- ---------- GQHASRRIIT
Raph-sat_KAN3                       ---------- ---------- ---------- ---------- ----------
Vig-ang_KAN2                        ---------- ---------- ---HHHHHLP LEP------- STTAPSSIIS
Med-tru_KAN2                        ---------- ---------- -----NHNHN LNHLHILDGT TTTTPSSISN
Vig-rad_KAN2                        ---------- ---------- ----HHHHLP LEP------- STTTPSSIIS
Glyc_KAN2                           ---------- ---------- ----HHHHLP LEA------- STTTPSIISN
Vit-vin_KAN2                        ---------- ---------- --------QP LDS-----SI ASSTTTNTAA
Theo-cac_KAN2                       ---------- ---------- --------QP LDS------S LASSPSSLVT
Pop-tri_hypothetical.protein        ---------- ---------- --------QT FDN------- TCSQASSLAA
Ric-com_KAN2                        ---------- ---------- -------PQT LDN------- SCSQTSSLAT
Ath_KAN4                            ---------- ---------- ---------- ---------- ----------
Ric-com_KAN4                        ---------- ---------- ---------- ---------- ----------
Pop-tri_KAN4                        ---------- ---------- ---------- ---------- ----------
Nel-nuc_KAN4                        ---------- ---------- ---------- ---------- ----------
The-cac_KAN4                        ---------- ---------- ---------- ---------- ----------
Vit-vin_KAN4                        ---------- ---------- ---------- ---------- ----------
Glycine_KAN4                        ---------- ---------- ---------- ---------- ----------
Sol-lyc_KAN4                        ---------- ---------- ---------- ---------- ----------
Med_tru_KAN4                        ---------- ---------- ---------- ---------- ----------
Vit-vin_KAN4                        ---------- ---------- ---------- ---------- ----------
Pop-tri_KAN4                        ---------- ---------- ---------- ---------- ----------
Sol_tub_KAN4                        ---------- ---------- ---------- ---------- ----------
Pru-per_KAN4                        ---------- ---------- ---------- ---------- ----------
Amb-tri_KAN4                        ---------- ---------- ---------- ---------- ----------
Fra-vesca_KAN4                      ---------- ---------- -------STP LPP------- ----------
Mus-acu_KAN4                        ---------- ---------- ---------- ---------- ----------
Glyc_KAN4                           ---------- ---------- ---------- ---------- ----------
Glyc-KAN4                           ---------- ---------- ---------- ---------- ----------
Cam-sat_KAN4                        ---------- ---------- ---------- ---------- ----------
Bra-rapa_KAN4                       ---------- ---------- ---------- ---------- ----------
Bra-nap_KAN4                        ---------- ---------- ---------- ---------- ----------
```

```
                                  301
Ath_KAN1                          SSSGY----- ---------- ---------- ---------- ----------
Amb-tric_KAN1                     SSSSALGLDP MATILQSSSA SQAQPFHSL- ---------- ----------
Cam-sat_KAN1                      SSSGY----- ---------- ---------- ---------- ----------
Bras-ole_KAN1-like                SSSGY----- ---------- ---------- ---------- ----------
Bra-nap_KAN1-like                 SSSGY----- ---------- ---------- ---------- ----------
Vit-vin_KAN1                      SSVSH---PP SPYF---GGG LDPMSILNSG ---PNTSSSA Y-----RIAA
Pru-mum_KAN1-variantX1            SNTPS----- TPYF---GGG LDPMS----- ---------- ----------
The-cacao_KAN1-variantX1          SSPSL---SA SPYF---EGG LDPISILSSG --------ST QASPAYRSPA
Ric-com_KAN1-variantX1            PSVSH---TS SPYY-IG--- -DPMSMLNSS SGVPNNHHQS LP-SYNRLAA
Pop-eup_KAN1                      YSSCS---TS SPYS-IGGGG LDPMSMLNSS G--PN---QA LPGPYNRLAP
Pru-per_KAN1-variantX1            SNTPS----- TPYF---GGG LDPMS----- ---------- ----------
Sol-tub_KAN1-like-variantX2       NRFNGISS-- ---------- ---------- ---------- ----------
Nel-nuc_KAN1-variantX1            SSCPSSASPS SPFM---AGG FDPMAILHSG P------NSP SPSSAYRIAA
Vig-rad_KAN1                      SSSSSFTSSP APYFAAAAGG PDPMSYLSSG G------TTV AAAAAYRAAT
Ath_KAN2                          NNNSFNTSSV SN-------- ---------- ---------- ----------
Amb-tri_KAN2                      SSPPS----- ---------- ---------- ---------- ----------
Ara-tha_GARP-like_KAN2            NNNSFNTSSV SN-------- ---------- ---------- ----------
Cam-sat_KAN2                      NNHSFNTSSV SNSGLITN-- ---------- ---------- ----------
Bra-nap_KAN2                      NSFGT----- ---------- ---------- ---------- ----------
Bra-rapa_KAN2                     NSFGT----- ---------- ---------- ---------- ----------
Cit-sin_KAN2                      NNTST----- ---------- ---------- ---------- ----------
Vit-vin_KAN2                      TTTGS----- ---------- ---------- ---------- ----------
The-cacao_KAN2                    NNTSN---TS L--------- ---------- ---------- ----------
Med-tru_KAN2                      TNNTSS---- ---------- ---------- ---------- ----------
Glycine_KAN2                      TNNGS----- ---------- ---------- ---------- ----------
Vig-rad_KAN2                      STNTSS---- ---------- ---------- ---------- ----------
Vig-ang_KAN2                      STNTAS---- ---------- ---------- ---------- ----------
Vit-vin_KAN2-variantX4            TTTGS----- ---------- ---------- ---------- ----------
Pop-trichocarpa                   NNSSATSI-- ---------- ---------- ---------- ----------
Ath_KAN3                          NPNCS----- ---------- ---------- ---------- ----------
Bra-napus_KAN3                    ---------- ---------- ---------- ---------- ----------
Bra-nap_KAN3                      ---------- ---------- ---------- ---------- ----------
Bra-nap_KAN3                      ---------- ---------- ---------- ---------- ----------
Cam-sat_KAN3                      NPNCS----- ---------- ---------- ---------- ----------
Raph-sat_KAN3                     ---------- ---------- ---------- ---------- ----------
Vig-ang_KAN2                      STNTAS---- ---------- ---------- ---------- ----------
Med-tru_KAN2                      TNNTSS---- ---------- ---------- ---------- ----------
Vig-rad_KAN2                      STNTSS---- ---------- ---------- ---------- ----------
Glyc_KAN2                         TNNGS----- ---------- ---------- ---------- ----------
Vit-vin_KAN2                      TTTGS----- ---------- ---------- ---------- ----------
Theo-cac_KAN2                     NNTSNTSL-- ---------- ---------- ---------- ----------
Pop-tri_hypothetical.protein      NNSSATSI-- ---------- ---------- ---------- ----------
Ric-com_KAN2                      NNSTT----- ---------- ---------- ---------- ----------
Ath_KAN4                          ---------- ---------- ---------- ---------- ----------
Ric-com_KAN4                      ---------- ---------- ---------- ---------- ----------
Pop-tri_KAN4                      ---------- ---------- ---------- ---------- ----------
Nel-nuc_KAN4                      ---------- ---------- ---------- ---------- ----------
The-cac_KAN4                      ---------- ---------- ---------- ---------- ----------
Vit-vin_KAN4                      ---------- ---------- ---------- ---------- ----------
Glycine_KAN4                      ---------- ---------- ---------- ---------- ----------
Sol-lyc_KAN4                      ---------- ---------- ---------- ---------- ----------
Med_tru_KAN4                      ---------- ---------- ---------- ---------- ----------
Vit-vin_KAN4                      ---------- ---------- ---------- ---------- ----------
Pop-tri_KAN4                      ---------- ---------- ---------- ---------- ----------
Sol_tub_KAN4                      ---------- ---------- ---------- ---------- ----------
Pru-per_KAN4                      ---------- ---------- ---------- ---------- ----------
Amb-tri_KAN4                      ---------- ---------- ---------- ---------- ----------
Fra-vesca_KAN4                    ---------- ---------- ---------- ---------- ----------
Mus-acu_KAN4                      ---------- ---------- ---------- ---------- ----------
Glyc_KAN4                         ---------- ---------- ---------- ---------- ----------
Glyc-KAN4                         ---------- ---------- ---------- ---------- ----------
Cam-sat_KAN4                      ---------- ---------- ---------- ---------- ----------
Bra-rapa_KAN4                     ---------- ---------- ---------- ---------- ----------
Bra-nap_KAN4                      ---------- ---------- ---------- ---------- ----------
```

```
                                   351
Ath_KAN1                            -NNAYRSLQS SPRLKGVPLH HHHHHNQY-- ----GVVGSS DSSSPHHHNH
Amb-tric_KAN1                       ---RFGGLSS -ETLKSHHHN HHHFGGVS-- ---------- ---------D
Cam-sat_KAN1                        -NNAYRSLQS SPRLKGVPLH HHHHHNHY-- ---GIVGSSD SSSPHHHNHH
Bras-ole_KAN1-like                  -NNAYRSLQS SPRLRGVPLH HHHHHNHY-- ---------G VVGSTDSSSP
Bra-nap_KAN1-like                   -NNAYRSLQS SPRLRGVPLH HHHHHNHY-- ---------G VVGSTDSSSP
Vit-vin_KAN1                        AA-RLNGLSS -DAFKSHQLH HHHVPSHH-- ----GV---- GPSE-----A
Pru-mum_KAN1-variantX1              ---RFNGFSS MDPFKSSNQL HHHHHLHHLH HNQYGIGGVG PISE-----A
The-cacao_KAN1-variantX1            TI-RFNGISK -DAFKSHQLH QQPQHSHY-- ----GI---- GSSD-----T
Ric-com_KAN1-variantX1              TA-RFNGLSM -DAFKS---H HQLHHHHH-- --QYGV---- GSGE-----A
Pop-eup_KAN1                        TT-RFNGLSM -DAFKSHQLH HHHHHHHN-- --QYGV---- GSTE-----G
Pru-per_KAN1-variantX1              ---RFNGFSS MDPFKSNNHL HHHHHLHHLH HNQYGIGGVG PISE-----A
Sol-tub_KAN1-like-variantX2         ---------- -SSYHHVPHH HPLQYHHH-- LSQYGHLGGL GHNSHHENIA
Nel-nuc_KAN1-variantX1              AASRFNGLSS -DTLKPHQLH SLHHPHQY-- ----GI---- GPSD-----A
Vig-rad_KAN1                        AT-RFNGISG -EAFKSHHPL HHHHSSSH-- ---YGV-GLG GSHE-----A
Ath_KAN2                            ---------- -PNYHNHHH- ---------- ---------- ----------
Amb-tri_KAN2                        ---------- -SSYNFGS-- ---------- ---------- ----------
Ara-tha_GARP-like_KAN2              ---------- -PNYHNHHH- ---------- ---------- ----------
Cam-sat_KAN2                        ---------- -PNYHNHHHH H--------- ---------- ----------
Bra-nap_KAN2                        ---------- -SRVNPNHHN HH-------- ---------- ----------
Bra-rapa_KAN2                       ---------- -SRVNPNHHN HH-------- ---------- ----------
Cit-sin_KAN2                        ---------- -SSLNSFQSQ ---------- ---------- ----------
Vit-vin_KAN2                        ---------- -SPFQSHH-- ---------- ---------- ----------
The-cacao_KAN2                      ---------- -SPFQS---- ---------- ---------- ----------
Med-tru_KAN2                        ---------- -SPFQS---- ---------- ---------- ----------
Glycine_KAN2                        ---------- -TPFHS---- ---------- ---------- ----------
Vig-rad_KAN2                        ---------- -TPFHS---- ---------- ---------- ----------
Vig-ang_KAN2                        ---------- -TPFHS---- ---------- ---------- ----------
Vit-vin_KAN2-variantX4              ---------- -SPFQSHH-- ---------- ---------- ----------
Pop-trichocarpa                     ---------- -SPYQS---- ---------- ---------- ----------
Ath_KAN3                            ---------- -FNLHNRHRR QAQPQP---- ---------- ----------
Bra-napus_KAN3                      ---------- -PPFFFSEVN GQHTNPSY-- ---------- ----------
Bra-nap_KAN3                        ---------- -PPFFFSEVN GQHANPSY-- ---------- ----------
Bra-nap_KAN3                        ---------- -PPFFFSEVN GQHANPSY-- ---------- ----------
Cam-sat_KAN3                        ---------- -YNLHHRNRR QAQPQP---- ---------- ----------
Raph-sat_KAN3                       ---------- -TPFFFSEVN GQHANPNY-- ---------- ----------
Vig-ang_KAN2                        ---------- -TPFHS---- ---------- ---------- ----------
Med-tru_KAN2                        ---------- -SPFQS---- ---------- ---------- ----------
Vig-rad_KAN2                        ---------- -TPFHS---- ---------- ---------- ----------
Glyc_KAN2                           ---------- -TPFHS---- ---------- ---------- ----------
Vit-vin_KAN2                        ---------- -SPFQSHH-- ---------- ---------- ----------
Theo-cac_KAN2                       ---------- -SPFQS---- ---------- ---------- ----------
Pop-tri_hypothetical.protein        ---------- -SPYQS---- ---------- ---------- ----------
Ric-com_KAN2                        ---------- -TNIGHFQS- ---------- ---------- ----------
Ath_KAN4                            ---------- ----HHHQRR SNMFQPQI-- ---------- ----------
Ric-com_KAN4                        ---------- -RNLNHHHHH QPQI------ ---------- ----------
Pop-tri_KAN4                        ---------- -RNLNHHHHQ PQI------- ---------- ----------
Nel-nuc_KAN4                        ---------- -PPMQLPRNL HYLHQPQI-- ---------- ----------
The-cac_KAN4                        ---------- -RNLNHHQHQ YQPQI----- ---------- ----------
Vit-vin_KAN4                        ---------- -RNLHHNHHN HQPQI----- ---------- ----------
Glycine_KAN4                        ---------- -RSFNHHHHL LQPHI----- ---------- ----------
Sol-lyc_KAN4                        ---------- -THFQYHHEH YQPQI----- ---------- ----------
Med_tru_KAN4                        ---------- -RNFNQHFQP HI-------- ---------- ----------
Vit-vin_KAN4                        ---------- -RNLHHNHHN HQPQI----- ---------- ----------
Pop-tri_KAN4                        ---------- -RNLNHLHHH NPQI------ ---------- ----------
Sol_tub_KAN4                        ---------- -KNFQY---H HQHYQPQI-- ---------- ----------
Pru-per_KAN4                        ---------- -RNFSNHHFH HHPQI----- ---------- ----------
Amb-tri_KAN4                        ---------- -DSFNGDAVI LGLGSHHH-- ---------- ---------L
Fra-vesca_KAN4                      ---------- -RNFNHHHHH HPQI------ ---------- ----------
Mus-acu_KAN4                        ---------- -HDLHHRDHH HHHHHDQL-- ---------- ---------R
Glyc_KAN4                           ---------- ---------- ---------- ---------- ----------
Glyc-KAN4                           ---------- -LSRNNFSHH LHNYQPHT-- ---------- ----------
Cam-sat_KAN4                        ---------- -HHQHHHQRH SNMFQPQI-- ---------- ----------
Bra-rapa_KAN4                       ---------- -PYHHHQRHS SNMFQPQI-- ---------- ----------
Bra-nap_KAN4                        ---------- -PYHHHQRHS SNMFQPQI-- ---------- ----------
```

```
                               401
Ath_KAN1                       HHHGMIRSRF LPKM-P---- --TKRSMRAP RMRWTSSLHA RFVHAVELLG
Amb-tric_KAN1                  ASSHAMRSRI VSKL-P---- --TKRSMRAP RMRWTSTLHA RFVHAVELLG
Cam-sat_KAN1                   HHHGMIRSRF LPKM-P---- --TKRSMRAP RMRWTSSLHA RFVHAVELLG
Bras-ole_KAN1-like             HHHGMIRSRF LPKM-P---- --TKRSMRAP RMRWTSSLHA RFVHAVELLG
Bra-nap_KAN1-like              HHHGMIRSRF LPKM-P---- --TKRSMRAP RMRWTSSLHA RFVHAVELLG
Vit-vin_KAN1                   S-HGMMRSRF IPKL-P---- --TKRSMRAP RMRWTSTLHA RFVHAVELLG
Pru-mum_KAN1-variantX1         SHHGIMRSRF LPKL-P---- --TKRSMRAP RMRWTSTLHA RFVHAVELLG
The-cacao_KAN1-variantX1       S-NGLIRSRF LPKL-P---- --TKRSMRAP RMRWTSTLHA RFVHAVELLG
Ric-com_KAN1-variantX1         S-HGLIRSRF LPKL-P---- --TKRSMRAP RMRWTSTLHA RFVHAVELLG
Pop-eup_KAN1                   SPHGLIRSRF FPKL-P---- --TKRSMRAP RMRWTSTLHA RFVHAVELLG
Pru-per_KAN1-variantX1         SHHGIMRSRF LPKL-P---- --TKRSMRAP RMRWTSTLHA RFVHAVELLG
Sol-tub_KAN1-like-variantX2    NSHSLMRSRF LPKL-P---- --AKRSMRAP RMRWTSTLHA RFVHAVELLG
Nel-nuc_KAN1-variantX1         S-HAMIRSRF MPKL-P---- --TKRNMRAP RMRWTSTLHA RFVHAVELLG
Vig-rad_KAN1                   S-SGLMRSRF LPKL-P---- --TKRSMRAP RMRWTSTLHA RFVHAVELLG
Ath_KAN2                       --QTLNRARF MPRF-P---- --AKRSMRAP RMRWTTTLHA RFVHAVELLG
Amb-tri_KAN2                   --NGGMRSRF MTRFTA---- --ARRSMRAP RMRWTSSLHA RFVHAVELLG
Ara-tha_GARP-like_KAN2         --QTLNRARF MPRF-P---- --AKRSMRAP RMRWTTTLHA RFVHAVELLG
Cam-sat_KAN2                   --QTLNRARF MPRF-P---- --AKRSMRAP RMRWTTTLHA RFVHAVELLG
Bra-nap_KAN2                   --QTLNRARF MPRF-P---- --AKRSMRAP RMRWTTTLHA RFVHAVELLG
Bra-rapa_KAN2                  --QTLNRARF MPRF-P---- --AKRSMRAP RMRWTTTLHA RFVHAVELLG
Cit-sin_KAN2                   --QGLMRSRF MSRF-P---- --AKRSMRAP RMRWTTTLHA RFVHAVELLG
Vit-vin_KAN2                   --QGLIRSRF LSRF-P---- --AKRSMRAP RMRWTTTLHA RFVHAVELLG
The-cacao_KAN2                 --QGLMRSRF MSRF-P---- --AKRSMRAP RMRWTTTLHA RFVHAVELLG
Med-tru_KAN2                   --QALMRSRF LSRF-P---- --AKRSMRAP RMRWTTTLHA RFVHAVELLG
Glycine_KAN2                   --QALMRSRF LSRF-P---- --AKRSMRAP RMRWTSTLHA RFVHAVELLG
Vig-rad_KAN2                   --QALMRSRF LSRF-P---- --AKRSMRAP RMRWTSTLHA RFVHAVELLG
Vig-ang_KAN2                   --QALMRSRF LSRF-P---- --AKRSMRAP RMRWTSTLHA RFVHAVELLG
Vit-vin_KAN2-variantX4         --QGLIRSRF LSRF-P---- --AKRSMRAP RMRWTTTLHA RFVHAVELLG
Pop-trichocarpa                --QGSMRSRF MSRF-P---- --AKRSMRAP RMRWTTTLHA RFVHAVELLG
Ath_KAN3                       ---------- -PRF-T---- --AKRGVRAP RMRWTTTLHA HFVHAVQLLG
Bra-napus_KAN3                 --SYNLHHRH HRQAQPQAQR LTAKRGVRAP RMRWTTTLHA HFVHAVQLLG
Bra-nap_KAN3                   --SYNLHHRH HRQVQPQAQR LTAKRGVRAP RMRWTTTLHA HFVHAVQLLG
Bra-nap_KAN3                   --SYNLHHRH HRQVQPQAQR LTAKRGVRAP RMRWTTTLHA HFVHAVQLLG
Cam-sat_KAN3                   ---------- -PRF-T---- --AKRGVRAP RMRWTATLHA HFVHAVQLLG
Raph-sat_KAN3                  --SYNLHHRP HRQAQP--QR LTAKRGVRAP RMRWTTTLHA HFVHAVQLLG
Vig-ang_KAN2                   --QALMRSRF LSRF-P---- --AKRSMRAP RMRWTSTLHA RFVHAVELLG
Med-tru_KAN2                   --QALMRSRF LSRF-P---- --AKRSMRAP RMRWTTTLHA RFVHAVELLG
Vig-rad_KAN2                   --QALMRSRF LSRF-P---- --AKRSMRAP RMRWTSTLHA RFVHAVELLG
Glyc_KAN2                      --QALMRSRF LSRF-P---- --AKRSMRAP RMRWTSTLHA RFVHAVELLG
Vit-vin_KAN2                   --QGLIRSRF LSRF-P---- --AKRSMRAP RMRWTTTLHA RFVHAVELLG
Theo-cac_KAN2                  --QGLMRSRF MSRF-P---- --AKRSMRAP RMRWTTTLHA RFVHAVELLG
Pop-tri_hypothetical.protein   --QGSMRSRF MSRF-P---- --AKRSMRAP RMRWTTTLHA RFVHAVELLG
Ric-com_KAN2                   --QGLMRSRL MSRF-P---- --AKRSMRAP RMRWTTTLHA RFVHAVELLG
Ath_KAN4                       --YGRDFKRS SSSMVG---- --LKRSIRAP RMRWTSTLHA HFVHAVQLLG
Ric-com_KAN4                   --YGREFKRN GRMISG---- --VKRSIRAP RMRWTTTLHA HFVHAVQLLG
Pop-tri_KAN4                   --YGRDFKRS ARMING---- --VKRSVRAP RMRWTTTLHA HFVHAVRLLG
Nel-nuc_KAN4                   --YGREFKRN SRMING---- --GKRSIRAP RMRWTTTLHA HFVHAVELLG
The-cac_KAN4                   --YSRDFKRN ARMING---- --VKRSIRAP RMRWTTTLHA HFVHAVQLLG
Vit-vin_KAN4                   --YGREFKRN SRMMNG---- --SKRSIRAP RMRWTTTLHA HFVHAVQLLG
Glycine_KAN4                   --YGRDFKRS ARVVNG---- --VKRSVRAP RMRWTTTLHA HFVHAVQLLG
Sol-lyc_KAN4                   --YGREFKRS SRLISG---- --VKRSVRAP RMRWTSTLHA HFVHAVQLLG
Med_tru_KAN4                   --YGRDFKRN TRVVNG---- --VKRSVRAP RMRWTTTLHS HFVHAVQLLG
Vit-vin_KAN4                   --YGREFKRN SRMMNG---- --SKRSIRAP RMRWTTTLHA HFVHAVQLLG
Pop-tri_KAN4                   --YGGDFKRS ARMISG---- --VRRSKRAP RMRWTTTLHA HFVHAVQLLG
Sol_tub_KAN4                   --YGREFKRS SRLISG---- --VKRSVRAP RMRWTSTLHA HFVHAVQLLG
Pru-per_KAN4                   --YGRQFKRN ARTVGN---- --VKRSIRAP RMRWTTTLHA HFVHAVQLLG
Amb-tri_KAN4                   PALGRDFKRN SRIMSCG--- --SKRSMRAP RMRWTTTLHA HFVHAVELLG
Fra-vesca_KAN4                 --YGREFKRS ARTVGN---- --VKRSVRAP RMRWTTTLHA HFVHAVQLLG
Mus-acu_KAN4                   HPQTYGFKRN SRSAQG---- --GKRSVRAP RMRWTTTLHA HFVHAVELLG
Glyc_KAN4                      ---------- ---------- ---------- MMRWTTTLHA HFVHAVQLLG
Glyc-KAN4                      --NTLDFKRN ARVIHG---- --VKRNIRAP RMRWTTTLHA HFVHAVQLLG
Cam-sat_KAN4                   --YGRDFKRS SSSMVG---- --LKRSIRAP RMRWTSTLHA HFVHAVQLLG
Bra-rapa_KAN4                  --YGRDFKRT SSSVVS---- --LKRSIRAP RMRWTSTLHA HFVHAVQLLG
Bra-nap_KAN4                   --YGRDFKRT SSSVVS---- --LKRSIRAP RMRWTSTLHA HFVHAVQLLG
```

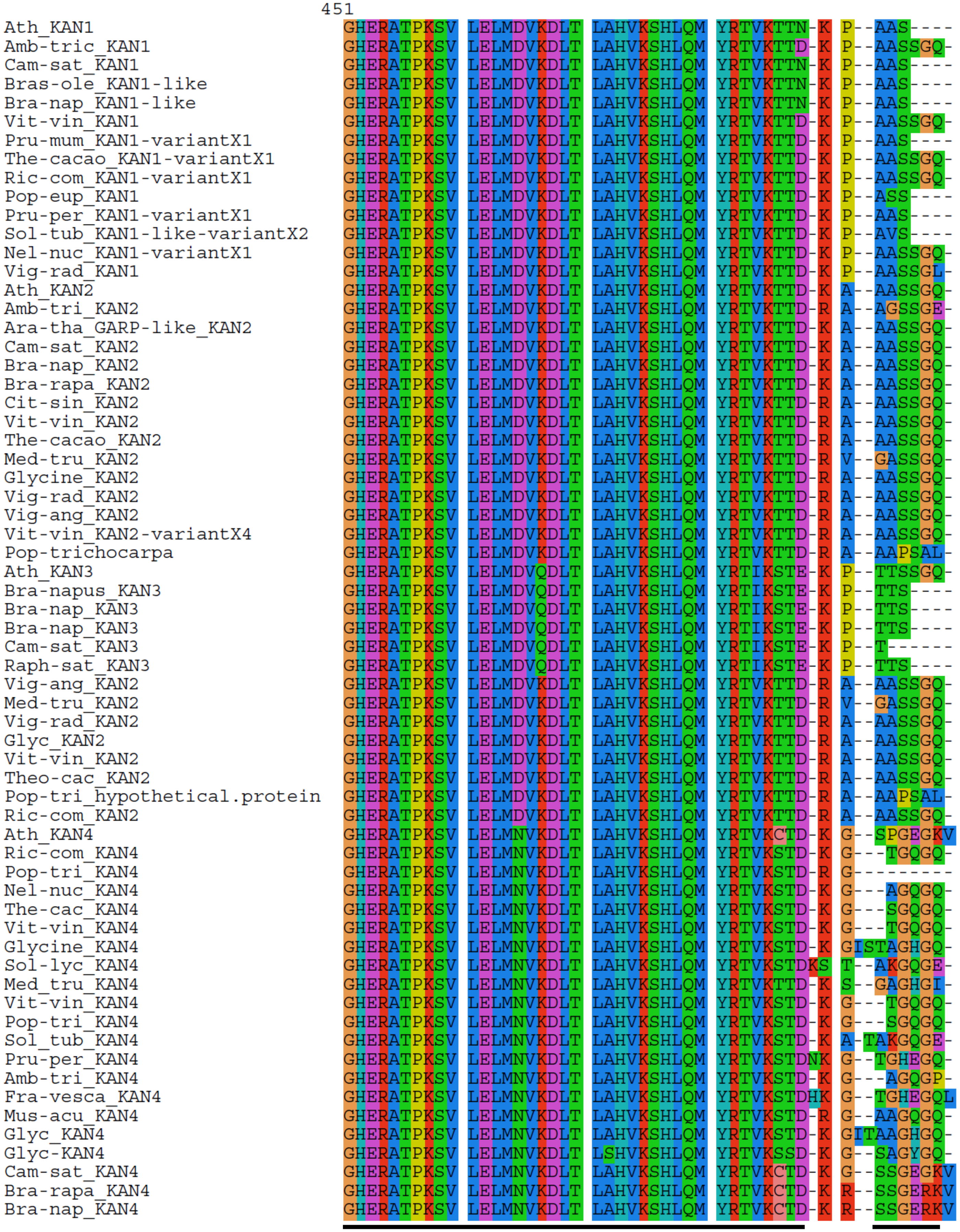
451
Ath_KAN1 GHERATPKSV LELMDVKDLT LAHVKSHLQM YRTVKTTN-K P--AAS----
Amb-tric_KAN1 GHERATPKSV LELMDVKDLT LAHVKSHLQM YRTVKTTD-K P--AASSGQ-
Cam-sat_KAN1 GHERATPKSV LELMDVKDLT LAHVKSHLQM YRTVKTTN-K P--AAS----
Bras-ole_KAN1-like GHERATPKSV LELMDVKDLT LAHVKSHLQM YRTVKTTN-K P--AAS----
Bra-nap_KAN1-like GHERATPKSV LELMDVKDLT LAHVKSHLQM YRTVKTTN-K P--AAS----
Vit-vin_KAN1 GHERATPKSV LELMDVKDLT LAHVKSHLQM YRTVKTTD-K P--AASSGQ-
Pru-mum_KAN1-variantX1 GHERATPKSV LELMDVKDLT LAHVKSHLQM YRTVKTTD-K P--AAS----
The-cacao_KAN1-variantX1 GHERATPKSV LELMDVKDLT LAHVKSHLQM YRTVKTTD-K P--AASSGQ-
Ric-com_KAN1-variantX1 GHERATPKSV LELMDVKDLT LAHVKSHLQM YRTVKTTD-K P--AASSGQ-
Pop-eup_KAN1 GHERATPKSV LELMDVKDLT LAHVKSHLQM YRTVKTTD-K P--ASS----
Pru-per_KAN1-variantX1 GHERATPKSV LELMDVKDLT LAHVKSHLQM YRTVKTTD-K P--AAS----
Sol-tub_KAN1-like-variantX2 GHERATPKSV LELMDVKDLT LAHVKSHLQM YRTVKTTD-K P--AVS----
Nel-nuc_KAN1-variantX1 GHERATPKSV LELMDVKDLT LAHVKSHLQM YRTVKTTD-K P--AASSGQ-
Vig-rad_KAN1 GHERATPKSV LELMDVKDLT LAHVKSHLQM YRTVKTTD-K P--AASSGL-
Ath_KAN2 GHERATPKSV LELMDVKDLT LAHVKSHLQM YRTVKTTD-K A--AASSGQ-
Amb-tri_KAN2 GHERATPKSV LELMDVKDLT LAHVKSHLQM YRTVKTTD-R A--AGSSGE-
Ara-tha_GARP-like_KAN2 GHERATPKSV LELMDVKDLT LAHVKSHLQM YRTVKTTD-K A--AASSGQ-
Cam-sat_KAN2 GHERATPKSV LELMDVKDLT LAHVKSHLQM YRTVKTTD-K A--AASSGQ-
Bra-nap_KAN2 GHERATPKSV LELMDVKDLT LAHVKSHLQM YRTVKTTD-K A--AASSGQ-
Bra-rapa_KAN2 GHERATPKSV LELMDVKDLT LAHVKSHLQM YRTVKTTD-K A--AASSGQ-
Cit-sin_KAN2 GHERATPKSV LELMDVKDLT LAHVKSHLQM YRTVKTTD-R A--AASSGQ-
Vit-vin_KAN2 GHERATPKSV LELMDVKDLT LAHVKSHLQM YRTVKTTD-R A--AASSGQ-
The-cacao_KAN2 GHERATPKSV LELMDVKDLT LAHVKSHLQM YRTVKTTD-R A--AASSGQ-
Med-tru_KAN2 GHERATPKSV LELMDVKDLT LAHVKSHLQM YRTVKTTD-R V--GASSGQ-
Glycine_KAN2 GHERATPKSV LELMDVKDLT LAHVKSHLQM YRTVKTTD-R A--AASSGQ-
Vig-rad_KAN2 GHERATPKSV LELMDVKDLT LAHVKSHLQM YRTVKTTD-R A--AASSGQ-
Vig-ang_KAN2 GHERATPKSV LELMDVKDLT LAHVKSHLQM YRTVKTTD-R A--AASSGQ-
Vit-vin_KAN2-variantX4 GHERATPKSV LELMDVKDLT LAHVKSHLQM YRTVKTTD-R A--AASSGQ-
Pop-trichocarpa GHERATPKSV LELMDVKDLT LAHVKSHLQM YRTVKTTD-R A--AAPSAL-
Ath_KAN3 GHERATPKSV LELMDVQDLT LAHVKSHLQM YRTIKSTE-K P--TTSSGQ-
Bra-napus_KAN3 GHERATPKSV LELMDVQDLT LAHVKSHLQM YRTIKSTE-K P--TTS----
Bra-nap_KAN3 GHERATPKSV LELMDVQDLT LAHVKSHLQM YRTIKSTE-K P--TTS----
Bra-nap_KAN3 GHERATPKSV LELMDVQDLT LAHVKSHLQM YRTIKSTE-K P--TTS----
Cam-sat_KAN3 GHERATPKSV LELMDVQDLT LAHVKSHLQM YRTIKSTE-K P--T------
Raph-sat_KAN3 GHERATPKSV LELMDVQDLT LAHVKSHLQM YRTIKSTE-K P--TTS----
Vig-ang_KAN2 GHERATPKSV LELMDVKDLT LAHVKSHLQM YRTVKTTD-R A--AASSGQ-
Med-tru_KAN2 GHERATPKSV LELMDVKDLT LAHVKSHLQM YRTVKTTD-R V--GASSGQ-
Vig-rad_KAN2 GHERATPKSV LELMDVKDLT LAHVKSHLQM YRTVKTTD-R A--AASSGQ-
Glyc_KAN2 GHERATPKSV LELMDVKDLT LAHVKSHLQM YRTVKTTD-R A--AASSGQ-
Vit-vin_KAN2 GHERATPKSV LELMDVKDLT LAHVKSHLQM YRTVKTTD-R A--AASSGQ-
Theo-cac_KAN2 GHERATPKSV LELMDVKDLT LAHVKSHLQM YRTVKTTD-R A--AASSGQ-
Pop-tri_hypothetical.protein GHERATPKSV LELMDVKDLT LAHVKSHLQM YRTVKTTD-R A--AAPSAL-
Ric-com_KAN2 GHERATPKSV LELMDVKDLT LAHVKSHLQM YRTVKTTD-R A--AASSGQ-
Ath_KAN4 GHERATPKSV LELMNVKDLT LAHVKSHLQM YRTVKCTD-K G--SPGEGKV
Ric-com_KAN4 GHERATPKSV LELMNVKDLT LAHVKSHLQM YRTVKSTD-K G---TGQGQ-
Pop-tri_KAN4 GHERATPKSV LELMNVKDLT LAHVKSHLQM YRTVKSTD-R G---------
Nel-nuc_KAN4 GHERATPKSV LELMNVKDLT LAHVKSHLQM YRTVKSTD-K G---AGQGQ-
The-cac_KAN4 GHERATPKSV LELMNVKDLT LAHVKSHLQM YRTVKSTD-K G---SGQGQ-
Vit-vin_KAN4 GHERATPKSV LELMNVKDLT LAHVKSHLQM YRTVKSTD-K G---TGQGQ-
Glycine_KAN4 GHERATPKSV LELMNVKDLT LAHVKSHLQM YRTVKSTD-K GISTAGHGQ-
Sol-lyc_KAN4 GHERATPKSV LELMNVKDLT LAHVKSHLQM YRTVKSTDKS T--AKGQGE-
Med_tru_KAN4 GHERATPKSV LELMNVKDLT LAHVKSHLQM YRTVKTTD-K S--GAGHGI-
Vit-vin_KAN4 GHERATPKSV LELMNVKDLT LAHVKSHLQM YRTVKSTD-K G---TGQGQ-
Pop-tri_KAN4 GHERATPKSV LELMNVKDLT LAHVKSHLQM YRTVKSTD-K G---SGQGQ-
Sol_tub_KAN4 GHERATPKSV LELMNVKDLT LAHVKSHLQM YRTVKSTD-K A-TAKGQGE-
Pru-per_KAN4 GHERATPKSV LELMNVKDLT LAHVKSHLQM YRTVKSTDNK G--TGHEGQ-
Amb-tri_KAN4 GHERATPKSV LELMNVKDLT LAHVKSHLQM YRTVKSTD-K G---AGQGP-
Fra-vesca_KAN4 GHERATPKSV LELMNVKDLT LAHVKSHLQM YRTVKSTDHK G--TGHEGQL
Mus-acu_KAN4 GHERATPKSV LELMNVKDLT LAHVKSHLQM YRTVKSTD-R G--AAGQGQ-
Glyc_KAN4 GHERATPKSV LELMNVKDLT LAHVKSHLQM YRTVKSTD-K GITAAGHGQ-
Glyc-KAN4 GHERATPKSV LELMNVKDLT LSHVKSHLQM YRTVKSSD-K G--SAGYGQ-
Cam-sat_KAN4 GHERATPKSV LELMNVKDLT LAHVKSHLQM YRTVKCTD-K G--SSGEGKV
Bra-rapa_KAN4 GHERATPKSV LELMNVKDLT LAHVKSHLQM YRTVKCTD-K R--SSGERKV
Bra-nap_KAN4 GHERATPKSV LELMNVKDLT LAHVKSHLQM YRTVKCTD-K R--SSGERKV

```
                                 501
Ath_KAN1                         ---SDG---- --SGE---EE MGING-NEVH HQSSTDQRAQ --------SD
Amb-tric_KAN1                    ---SDGFD-S GSAGE---DD FVAGNLAGSD RNLGAFGF-- --------QG
Cam-sat_KAN1                     ---SDG---- --SGE---EE MGING-NEVH HQSSTDQRAQ --------SD
Bras-ole_KAN1-like               ---SDG---- --SGE---EE MGING-NDVH HQSSTDQKAQ --------SD
Bra-nap_KAN1-like                ---SDG---- --SGE---EE MGING-NDVH HQSSTDQKAQ --------SD
Vit-vin_KAN1                     ---SDG---- --SGE---ED I-SPM-GN-- --ASDCGLCR FTDQRGA-SE
Pru-mum_KAN1-variantX1           ---SDG---- --SGE---DD ILSPI-GSAT DLHGLRSPHQ FPEQRGP-SD
The-cacao_KAN1-variantX1         ---SDG---- --SGE---ED ISTAVSGSER GLRGLIDQ-- --------IG
Ric-com_KAN1-variantX1           ---SDG---- --SGE---ED I-STM-GS-G NDRGSGGLRR FSDQRGA-SD
Pop-eup_KAN1                     ---SDG---- --SGE---ED M-SPI-GSYG TANERGGL-- ---QRGVQSD
Pru-per_KAN1-variantX1           ---SDG---- --SGE---DD ILSPI-GSAT DLHGLRSPHQ FPEQRGP-SD
Sol-tub_KAN1-like-variantX2      ---SDG---- --SGE---DD L-TTI-GSTG G-GDRAGLGQ FMDQRGG-SD
Nel-nuc_KAN1-variantX1           ---SDG---- --SGE---ED F-SPA-AT-- --NNDLNLRR FIDQRGS-TD
Vig-rad_KAN1                     ---SDG---- --SGE---DD I-SPM-GSSG GMRQFSDQRS L-------SD
Ath_KAN2                         ---SDVYE-N GSSGDNNSDD WMFDM-NRKS RDSEEL--TN P-------LE
Amb-tri_KAN2                     ---IDIFG-N GTPGEISNEI LLENM----- ---------- --------TQ
Ara-tha_GARP-like_KAN2           ---SDVYE-N GSSGDNNSDD WMFDM-NRKS RDSEEL--TN P-------LE
Cam-sat_KAN2                     ---SDVYE-N GSSGDNNSDD WMFDM-NRKS RDSEEL--TN P-------LE
Bra-nap_KAN2                     ---SDVYE-N GSSGDNNSDD WMFDM-NRKS RDTDEL--TN P-------LE
Bra-rapa_KAN2                    ---SDVYE-N GSSGDNNSDD WMFDM-NRKS RDTDEL--TN P-------LE
Cit-sin_KAN2                     ---SDAFE-N GSSGD-TSED IMFGI-QHPR R-PETSSIQQ --------QG
Vit-vin_KAN2                     ---SDVYE-N GSSGD-TSED VMFDI-QNPR K-SELS--IQ --------QG
The-cacao_KAN2                   ---SDAFE-N GSSGD-TSED LMFDI-QNPR R-SEIS--VQ --------QG
Med-tru_KAN2                     ---SDVYD-N GSSGD-NSDD IMFDI-NSSR RSSDLS--IK Q-------QG
Glycine_KAN2                     ---SDVYD-N GSSGD-TSDD LMFDI-KSSR R-SDLS--VK --------QG
Vig-rad_KAN2                     ---SDVYD-N GSSGD-TSDD LMFDI-KSSR R-SDVS--IK --------QG
Vig-ang_KAN2                     ---SDVYD-N GSSGD-TSDD LMFDI-KSSR R-SDVS--IK --------QG
Vit-vin_KAN2-variantX4           ---SDVYE-N GSSGD-TSED VMFDI-QNPR KSELSIQ--- --------QG
Pop-trichocarpa                  ---SDVFD-N GSSGD-TSED LVFDI-ENPR R-SEMS--MQ --------QG
Ath_KAN3                         ---SDC---- -ENGS----- ---QV-NSER EARNL----- ----------
Bra-napus_KAN3                   ---SDIGQSD TCENE----- --LKV-NSER QARDL----- ----------
Bra-nap_KAN3                     ---SDIGQSD TCENE----- --LKV-NSER HARDL----- ----------
Bra-nap_KAN3                     ---SDIGQSD TCENE----- --LKV-NSER HARDLQG--- ----------
Cam-sat_KAN3                     ---------- ---------- ---------- ---------- ----------
Raph-sat_KAN3                    ---SDIEQSD TFEKE----- --LKV-NSER QAKDL----- ----------
Vig-ang_KAN2                     ---SDVYD-N GSSGD-TSDD LMFDI-KSSR R-SDVS--IK --------QG
Med-tru_KAN2                     ---SDVYD-N GSSGD-NSDD IMFDI-NSSR RSSDLS--IK Q-------QG
Vig-rad_KAN2                     ---SDVYD-N GSSGD-TSDD LMFDI-KSSR R-SDVS--IK --------QG
Glyc_KAN2                        ---SDVYD-N GSSGD-TSDD LMFDI-KSSR R-SDLS--VK --------QG
Vit-vin_KAN2                     ---SDVYE-N GSSGD-TSED VMFDI-QNPR K-SELS--IQ --------QG
Theo-cac_KAN2                    ---SDAFE-N GSSGD-TSED LMFDI-QNPR R-SEIS--VQ --------QG
Pop-tri_hypothetical.protein     ---SDVFD-N GSSGD-TSED LVFDI-ENPR R-SEMS--MQ --------QG
Ric-com_KAN2                     ---SDIFD-N GSSGD-TSED LMFEI-QNSR R-SDIS--MQ --------QG
Ath_KAN4                         EKEAEQRIED NNNNE--EAD EGTDT-NSPN SSSVQK---- --------TQ
Ric-com_KAN4                     ---TDMGL-K QRAGI---VD VDAGV-SAGK ADANPCCSLN PPPPP---TP
Pop-tri_KAN4                     ----PGKLYC LSSGKADANN LPQPL-NNPP PSPPPP---- ----------
Nel-nuc_KAN4                     ---TELGL-N QRSGV---IE VDAGLSSCEK TDPNPYSLNP P-------LP
The-cac_KAN4                     ---TEMSL-N QRTGI---VD LDGRL-SCGK ADTNPSYSLK --------PS
Vit-vin_KAN4                     ---TDMGL-N QRTGI----- --GQV-ELGG LSCDKADATP SF------SS
Glycine_KAN4                     ---TDIGL-N PRLGI---NV HLHAP-SPIC DTPNLPDPIQ N-------SQ
Sol-lyc_KAN4                     ---INIFL-N QNNGI----- ---NN-GEYE KSCEEIDALS QPSTISQNMA
Med_tru_KAN4                     ---------L QTQGI----- NIVPL-HGAN SSADERPNLP Q---------
Vit-vin_KAN4                     ---TDMGL-N QRTGIGQVEL GGLSC-DKAD ATPSFS---- --------SN
Pop-tri_KAN4                     ---TDMGL-N QRARI----- ---------- ---------- ----------
Sol_tub_KAN4                     ---IKIFL-N QNNGI----- ------NNGE ---------- --------YE
Pru-per_KAN4                     ---TDMGL-N QKPGI----- -------NGG LSCDK----- --------AN
Amb-tri_KAN4                     ---TEMGL-R QRSRV----- ------SQPE SGGFLRERAN --------HF
Fra-vesca_KAN4                   ---MDMGL-D QNPGN---NN VQVNG-SALP CDSEKA---- --------ID
Mus-acu_KAN4                     ---AEMCF-N QRRGM---EE VEGGL-SCDK AGNEITPPCS S-------LS
Glyc_KAN4                        ---TGIGLMN PRPGI---NV HLHAL-SPIC DTPNLPDPIQ S-------SH
Glyc-KAN4                        ---TGIGL-S QKPGI---VD LHGVL-ACER PDLPQPLLK- --------FQ
Cam-sat_KAN4                     EKEAEQRMED NNNNE--EAD EGTDT-NSPN SSSVQK---- --------TQ
Bra-rapa_KAN4                    EKEAEQKTED NNNNN---ED ADADP-ISPN SSSVQK---- --------TQ
Bra-nap_KAN4                     EKEAEQKTED NNNNN---ED ADADP-ISPN SSSVQK---- --------TQ
```

```
                                  551
Ath_KAN1                          DT--SLHQET DISS----TQ PRWSNSS--R ETWPLSNNCS SDIDTMIRTS
Amb-tric_KAN1                     EVDYSSTNNS HPPA-----P SLWSNSSS-R GPWLHTTSS- -DAESIRSPA
Cam-sat_KAN1                      DT--SLHQEI DISS----TQ PRWSNSS--R ETWPLSNNCS SDMDTLIRTS
Bras-ole_KAN1-like                DT--SPHQEI DFPS----TQ PRWSNSS--R ETWPLSNNYS SDVDTMIRTS
Bra-nap_KAN1-like                 DT--SPHQEI DFPS----TQ PRWSNSS--R ETWPLSNNYS SDVDTMIRTS
Vit-vin_KAN1                      GS---LQQEI DYPS-----T NLWSNSSSSR EAWLQQNSS- -EMDGLK-TA
Pru-mum_KAN1-variantX1            RS--SVQPDL DYTS----ST TLWSNSSSSR EAFPHTSSH- -DMDGLT-PV
The-cacao_KAN1-variantX1          TSDGSLQQEL DYPC--NPTT TLWSNSS--R EAWLHANSN- -DIDGVRGPL
Ric-com_KAN1-variantX1            GS---VQQEM SYPS---TAT TLWSNSSSSR EAWPQTNSN- -DPDGIR-EA
Pop-eup_KAN1                      GS--PVQQDM DYPSST-TTT TLWSNSSSGR EAWPQTNSN- -EMDGHR-QG
Pru-per_KAN1-variantX1            RS--SVQPDL DYTS----ST TLWSNSSSSR EAFPHTSSH- -DMDGLT-PV
Sol-tub_KAN1-like-variantX2       GS---LQPEP DYPS--TATN TLWSNSSSSR EGWLQANSN- -DTNVLMRSH
Nel-nuc_KAN1-variantX1            GS---VQQDT DYPS----TA TLWSNSSSNR GSWLQTNSS- -DMDGHR-SE
Vig-rad_KAN1                      RP---VQQDM DYSS----AN TLWSNSSSNR ELWQQNSSN- -DINGFR-PP
Ath_KAN2                          KS-------- --NG------ -LWTNSS--G EARLHGKLI- -DNVAEI-ML
Amb-tri_KAN2                      RP--PLQHGL DYCN------ -MWSNSSS-R RNWLQAKSR- -DSMSMS-PS
Ara-tha_GARP-like_KAN2            KS-------- --NG------ -LWTNSS--G EARLHGKLI- -DNVAEI-ML
Cam-sat_KAN2                      KG-------- --NG------ -LWTNSS--G EARLHGKLI- -DNVAEI-ML
Bra-nap_KAN2                      KS-------- --NG------ -RWTNSS--G EARLHGKLI- -DNVAEI-IL
Bra-rapa_KAN2                     KS-------- --NG------ -RWTNSS--G EARLHGKLI- -DNVAEI-IL
Cit-sin_KAN2                      RA-------- -SNG------ -LWSNSS--R EAWLHGKTK- -DSAINL-P-
Vit-vin_KAN2                      RP--NVHQEK DYHG------ -LWSNSS--R EAWLHGKQR- -DSGGNT-P-
The-cacao_KAN2                    RPSSNAHQDK EYHG------ -LWSNSS--R EAWLHGKPK- -DSARNL-P-
Med-tru_KAN2                      RS--SVNQDK ECYG------ -LWSNSS--R EAWLHGKPKV -DSIGNM-Q-
Glycine_KAN2                      RS--SVNQDK EYHG------ -LWGNSS--R EAWLHGKTKT -DSVGNV-PF
Vig-rad_KAN2                      RS--SVNQDK DYHG------ -LWGNSS--R EAWLHGKTRT -DSVGNV-P-
Vig-ang_KAN2                      RS--SVNQDK DCHG------ -LWGNSS--R EAWLHGKTKT -DSVGNV-P-
Vit-vin_KAN2-variantX4            RP--NVHQEK DYHG------ -LWSNSS--R EAWLHGKQR- -DSGGNTPTL
Pop-trichocarpa                   RP--NAHQDK DYQA------ -LWSNSS--R EAWLHGKLK- -DSDGNL-P-
Ath_KAN3                          ---------- --TG------ -LWNNSS--S EARFQLKAK- -ASSGVD-IS
Bra-napus_KAN3                    ---------- --QG------ -LWTNSS--S EARFHLKAKA SGLDMSS---
Bra-nap_KAN3                      ---------- --QG------ -LWSNSS--S EARFHLKAKA SGLDMSS---
Bra-nap_KAN3                      ---------- ---------- -LWSNSS--S EARFHLKAKA SGLDMSS---
Cam-sat_KAN3                      ---------- ---------- ---------- ---------- ----------
Raph-sat_KAN3                     ---------- --QG------ -LWNNSS--S ESRFHLKAKA SGLDITS---
Vig-ang_KAN2                      RS--SVNQDK DCHG------ -LWGNSS--R EAWLHGKTKT -DSVGNV-P-
Med-tru_KAN2                      RS--SVNQDK ECYG------ -LWSNSS--R EAWLHGKPKV -DSIGNM-Q-
Vig-rad_KAN2                      RS--SVNQDK DYHG------ -LWGNSS--R EAWLHGKTRT -DSVGNV-P-
Glyc_KAN2                         RS--SVNQDK EYHG------ -LWGNSS--R EAWLHGKTKT -DSVGNV-PF
Vit-vin_KAN2                      RP--NVHQEK DYHG------ -LWSNSS--R EAWLHGKQR- -DSGGNT-P-
Theo-cac_KAN2                     RPSSNAHQDK EYHG------ -LWSNSS--R EAWLHGKPK- -DSARNL-P-
Pop-tri_hypothetical.protein      RP--NAHQDK DYQA------ -LWSNSS--R EAWLHGKLK- -DSDGNL-P-
Ric-com_KAN2                      RP--NGHHDK DYQG------ -LWSNSS--R EAWLHVKSK- -DSCGNL-T-
Ath_KAN4                          RA-------- ---------- -SWSST---K EVSRSISTQA YSHLGTT-HH
Ric-com_KAN4                      TTPLPSVQKT QIRG------ -SWSSSMETR DINIS----- -NSEALIYSH
Pop-tri_KAN4                      ---------- ---------- -PLSSTQ--K NQRSLGDEK- ----------
Nel-nuc_KAN4                      KS-------- -PRG------ -SWSSME--T NGWDPST--- -KESGLIPSH
The-cac_KAN4                      SPSSQPTPQR TQSG------ -SWLSST--K TNNLSISSH- --GNGLTFKP
Vit-vin_KAN4                      NTPQPSTPQK ISRS------ -SWSLSM--- ETNDEGRLS- -HENGLKYSH
Glycine_KAN4                      RT-------- ---------- -PWQSSI--- ETKTDNSRQE EPEIGLTYSH
Sol-lyc_KAN4                      HMRDPSSFMP DQTN------ -VWSHSN--R ERTFAN---- ----------
Med_tru_KAN4                      ---------- ---------- -PLQNSL--R TSWQPSIETN TNNTEEKSEI
Vit-vin_KAN4                      TPQPSTPQKI SRLG----IR SSWSLSM--- ETNDEGRLS- -HENGLKYSH
Pop-tri_KAN4                      ---------- ---------- ---------- ---------- ----------
Sol_tub_KAN4                      KS-------- ---------- ---------- ---------- ----------
Pru-per_KAN4                      RG-------- ---------- -SWPSSSETN GSCNLSPGN- -ALTYSHSPQ
Amb-tri_KAN4                      FSSENVPSHV VSRG------ -SWLDSLTKE NESIYSQAET NQYTNLEVEV
Fra-vesca_KAN4                    SPSTLLLQNP SIRG------ -SWHSTR--- --KTNGSCK- -PSPGDFLTC
Mus-acu_KAN4                      TPTPPTPQSK SPRK------ ---LNPSGEG CAWNIARLLN NDNKVLL---
Glyc_KAN4                         RT-------- ---------- -PWQSSI--- ETKTNNRRQE EPEIGLTYSH
Glyc-KAN4                         RA-------- ---------- -SWQSSK--E TNSINYTQN- -PIINSMYSH
Cam-sat_KAN4                      RA-------- ---------- -SWSSTK--G VSMSISKHAE -PQLGTT-HH
Bra-rapa_KAN4                     RS-------- ---------- -PWSSTK--- ---------- ---------GV
Bra-nap_KAN4                      RS-------- ---------- -PWSSTK--- ---------- ---------GV
```

```
                              601
Ath_KAN1                      STS-MISHYQ RSS---IQNQ E-QRSNDQAK RCGNLSC-EN PSLEFTLGRP
Amb-tric_KAN1                 LLL-QEKDGH HIE------- ---------A KGNNHDL-KN PSLEFTLGRP
Cam-sat_KAN1                  STS-MISHHQ RSS---LQNQ E-QRSNDQAK RCGDLSC-NN PSLEFTLGRP
Bras-ole_KAN1-like            STS-VISHHQ RSI---LQNQ EQQRSSDQTK RCGDLSC-NN PSLEFTLGRP
Bra-nap_KAN1-like             STS-VISHHQ RSI---LQNQ EQQRSSDQTK RCGDLSC-NN PSLEFTLGRP
Vit-vin_KAN1                  SFP-SQQRS- GHQ---IEEC NST-LLKDYL GP-NLDC-KN PSLEFTLGRP
Pru-mum_KAN1-variantX1        SSQ-LQKIS- GHQ---IQEC DSTTQLKSYL GS-NFET-RN PSLEFTLGRP
The-cacao_KAN1-variantX1      SFQSNRTEES CDS------- ---------- -TPLLNC-KN PSLEFTLGRP
Ric-com_KAN1-variantX1        TVQ-SQQRS- GHP---VQEC NSN-RMKGYL GS-NLDC-KN PSLEFTLGRP
Pop-eup_KAN1                  TFQ-SQQRS- GQL---MEES DST-RPKSYL GS-SLAC-KN PSLEFTLGRP
Pru-per_KAN1-variantX1        SSQ-LQKIS- GHQ---IQEC ESTTQLKSYL GS-NFET-RN PSLEFTLGRP
Sol-tub_KAN1-like-variantX2   SFP-SQQRS- GHQ---IEEC NSSRQKSYNI GS-NLDHQKN PSLEFTLGRP
Nel-nuc_KAN1-variantX1        TFP-SQQRS- GHQ---IEES DLT-RTKSFL GS-HLEL-KN PSLEFTLGRP
Vig-rad_KAN1                  IFQ-SQQISG GHQ---IQDC DSSQLKNSLS GSGNLEC-KN PSLEFTLGRP
Ath_KAN2                      PSE-KELDGK CSS---YERI SSEEMSSSSI SGTSPFK-PN --LEFTLGRS
Amb-tri_KAN2                  SEVKGGLEDT SDE---LFEI SSSDMNQ--- --------KK PNLEFTLGRP
Ara-tha_GARP-like_KAN2        PSE-KELDGK CSS---YERI SSEEMSSSSI SGTSPFK-PN --LEFTLGRS
Cam-sat_KAN2                  PS-------- ---------- ---------- ---------- ---EFVL*--
Bra-nap_KAN2                  PSE-NEIDGK SSS---YERM SSEDMSSSNI SGTSPFK-PN --LEFTLG*-
Bra-rapa_KAN2                 PSE-NEIDGK SSS---YERM SSEDMSSSNI SGTSPFK-PN --LEFTLG*-
Cit-sin_KAN2                  SLE--EVDPK CMS---YEGI --SDVSSPNI SATSPKK-PN --LEFTLGRP
Vit-vin_KAN2                  TLE-EDMDPK CLS---YERI --SDVSSSNL SGTSPKK-PN --LEFTLGRS
The-cacao_KAN2                SLE-KDMDPK CLS---YERI --SDVSSSSL SGTSPKK-PN --LEFTLGVP
Med-tru_KAN2                  SLE-KEMDPK CLS---YERI -SDGSSSTNL SGSSPKK-PN LDLEFTLGQP
Glycine_KAN2                  SLE-KEMDPK CLS---YERI -SDGSSSSNL SGSSPKK-PN LDLEFSLGQP
Vig-rad_KAN2                  SLE-KEMDPK CVS---YERI -SDGSSSSNV SGSSPKK-AN LDLEFSLGQP
Vig-ang_KAN2                  SLE-KEMDPK CVS---YERI -SDGSSSSNV SGSSPKK-AN LDLEFSLGQP
Vit-vin_KAN2-variantX4        ERDGVKRWHH TIM------- ---------- --------PN LHHLFQIS*-
Pop-trichocarpa               PLE-KGMDPK CLS---YEKI --SDVSSSTV SGTSPKK-PN --LEFTLGRP
Ath_KAN3                      SNENEWKNRR CPS---NERL SSD---SSSL TGTRPET-ET PNLDFTLATP
Bra-napus_KAN3                ---NKNVDQR CPS---YERL SSD---SSSL TGTRPEI-ET PNLEFTLAIP
Bra-nap_KAN3                  ---NKNVDQR CPS---YERL SSD---SSSL TGTRPEI-ET PNLEFTLAIP
Bra-nap_KAN3                  ---NKNVDQR CPS---YERL SSD---SSSL TGTRPEI-ET PNLEFTLAIP
Cam-sat_KAN3                  ---------- ---------- ---------- --------XN YYISLFLSNP
Raph-sat_KAN3                 ---NKNVDQR CPS---YERL SSD---SSSL TGTRPEI-ET PNLEFTLAIP
Vig-ang_KAN2                  SLE-KEMDPK CVS---YERI -SDGSSSSNV SGSSPKK-AN LDLEFSLGQP
Med-tru_KAN2                  SLE-KEMDPK CLS---YERI -SDGSSSTNL SGSSPKK-PN LDLEFTLGQP
Vig-rad_KAN2                  SLE-KEMDPK CVS---YERI -SDGSSSSNV SGSSPKK-AN LDLEFSLGQP
Glyc_KAN2                     SLE-KEMDPK CLS---YERI -SDGSSSSNL SGSSPKK-PN LDLEFSLGQP
Vit-vin_KAN2                  TLE-EDMDPK CLS---YERI --SDVSSSNL SGTSPKK-PN --LEFTLGRS
Theo-cac_KAN2                 SLE-KDMDPK CLS---YERI --SDVSSSSL SGTSPKK-PN --LEFTLGVP
Pop-tri_hypothetical.protein  PLEQKGMDPK CLS---YEKI --SDVSSSTV SGTSPKK-PN --LEFTLGRP
Ric-com_KAN2                  SLE------- ---------- ---------- ---------- --------VP
Ath_KAN4                      TKDNEEKEDT N--------- ---------- --------IH LNLDFTLGRP
Ric-com_KAN4                  FSAHDTKENG PMADLHMSDR VKESLDSSSL SSTD----ML VNLEFTLGRP
Pop-tri_KAN4                  ----NISSLE ALTYVNVEAH DHSKRLDSSS SSSSD---ML LNLEFTLGRP
Nel-nuc_KAN4                  LKSTDLTNEK NKMDGADFYV SHNESERLYL SPLASSH-VL PNLEFTLGRQ
The-cac_KAN4                  NHVKVDGDKA VLH---VSDR MKERLDSSSL SPSD----MF LNLEFTLGRP
Vit-vin_KAN4                  LMANDTKMEG HKV---ALHV AEGPKERVDS SALSPSD-ML LNLEFTLGRP
Glycine_KAN4                  LKGNNNNNET MVD----GHN NSGGLDSSPL SRSSE---AM LDLEFTLGRP
Sol-lyc_KAN4                  ---------- ------YPHI YTNMDGIETA NNSSSND-TM LDLEFTLGRP
Med_tru_KAN4                  GLTYSQLKEN DTM---VKRL DSA------- --------QL IDLEFTLGRP
Vit-vin_KAN4                  LMANDTKMEG HKVALHVAEG PKERVDSSAL SPSD----ML LNLEFTLGRP
Pop-tri_KAN4                  ---------- ---------- ---------- ---------- LEVDAA----
Sol_tub_KAN4                  ---------- ------FEEI ---------- --------DA LSQPSTISQN
Pru-per_KAN4                  SNGTKVEEEE SGRHVCDDGV GMKERGTTLA CGSLSASDMY LNLEFTLGRP
Amb-tri_KAN4                  DKYKEASDSY HEE---AEKW HSSSYPSPQL TSSRLLS-KL PNLEFTLGRE
Fra-vesca_KAN4                SDSPSSIGTK VDE---EDSG SGRQLDCSSL SSSDH---MY LNLEFTLGRP
Mus-acu_KAN4                  ---------- ------MEDQ PQELRPTLVP PSMPP---RM PNLEITLGRQ
Glyc_KAN4                     LKGNNNNNET TVD----GHN NYGGLDSTPL SRSEE---AM LDLEFTLGRP
Glyc-KAN4                     LKG-NQTMVG GQHYGGLSNC MKEKLDSCSL SRSD----MT LDLEFTLGRP
Cam-sat_KAN4                  TKENKEKEAT N--------- ---------- --------VY LNLEFTLGRP
Bra-rapa_KAN4                 SMSISQADPH ME----INRI TKDDVEK--- --------DH LSLEFTLGRP
Bra-nap_KAN4                  SMSISQADPH ME----INRI TKDDVEK--- --------DH LSLEFTLGRP
```

```
                               651
Ath_KAN1                       --DWHEK*-- ---------- ---------- ---------- ----------
Amb-tric_KAN1                  --GWHGKDRD *--------- ---------- ---------- ----------
Cam-sat_KAN1                   --DWHDK*-- ---------- ---------- ---------- ----------
Bras-ole_KAN1-like             --DWHEK*-- ---------- ---------- ---------- ----------
Bra-nap_KAN1-like              --DWHEK*-- ---------- ---------- ---------- ----------
Vit-vin_KAN1                   --DWHGKEHD *--------- ---------- ---------- ----------
Pru-mum_KAN1-variantX1         --DWNGKPHD *--------- ---------- ---------- ----------
The-cacao_KAN1-variantX1       --DWHGTQHK HT*------- ---------- ---------- ----------
Ric-com_KAN1-variantX1         --DWQGNEH* ---------- ---------- ---------- ----------
Pop-eup_KAN1                   --DWPGKEHC *--------- ---------- ---------- ----------
Pru-per_KAN1-variantX1         --DWNGKPHD *--------- ---------- ---------- ----------
Sol-tub_KAN1-like-variantX2    --DWIEKDHE *--------- ---------- ---------- ----------
Nel-nuc_KAN1-variantX1         --DWCGQEHD *--------- ---------- ---------- ----------
Vig-rad_KAN1                   --DWNGKGQA *--------- ---------- ---------- ----------
Ath_KAN2                       --H*------ ---------- ---------- ---------- ----------
Amb-tri_KAN2                   --D*------ ---------- ---------- ---------- ----------
Ara-tha_GARP-like_KAN2         --H*------ ---------- ---------- ---------- ----------
Cam-sat_KAN2                   ---------- ---------- ---------- ---------- ----------
Bra-nap_KAN2                   ---------- ---------- ---------- ---------- ----------
Bra-rapa_KAN2                  ---------- ---------- ---------- ---------- ----------
Cit-sin_KAN2                   --H*------ ---------- ---------- ---------- ----------
Vit-vin_KAN2                   --H*------ ---------- ---------- ---------- ----------
The-cacao_KAN2                 --H*------ ---------- ---------- ---------- ----------
Med-tru_KAN2                   --L*------ ---------- ---------- ---------- ----------
Glycine_KAN2                   --L*------ ---------- ---------- ---------- ----------
Vig-rad_KAN2                   --L*------ ---------- ---------- ---------- ----------
Vig-ang_KAN2                   --L*------ ---------- ---------- ---------- ----------
Vit-vin_KAN2-variantX4         ---------- ---------- ---------- ---------- ----------
Pop-trichocarpa                --H*------ ---------- ---------- ---------- ----------
Ath_KAN3                       --NLSP*--- ---------- ---------- ---------- ----------
Bra-napus_KAN3                 --NLPP*--- ---------- ---------- ---------- ----------
Bra-nap_KAN3                   --NLPP*FTI YTIKTKKNC* NIQI*TLKLC LRYEKNMK*C LLLRLDICSF
Bra-nap_KAN3                   --NLPP*FTI YTIKTKKNC* NIQI*TLKLC LRYEKNMK*C LLLRLDICSF
Cam-sat_KAN3                   --KIST*--- ---------- ---------- ---------- ----------
Raph-sat_KAN3                  --NLPP*--- ---------- ---------- ---------- ----------
Vig-ang_KAN2                   --L*QGSDPS EYVLLIN**E TERGRELERE RGSR*ENERK RKLKENKREK
Med-tru_KAN2                   --L*------ ---------- ---------- ---------- ----------
Vig-rad_KAN2                   --L*------ ---------- ---------- ---------- ----------
Glyc_KAN2                      --L*------ ---------- ---------- ---------- ----------
Vit-vin_KAN2                   --H*------ ---------- ---------- ---------- ----------
Theo-cac_KAN2                  --H*------ ---------- ---------- ---------- ----------
Pop-tri_hypothetical.protein   --H*SYPLCT THFESLLSLR GCW*RESGTQ VRKSELKKKK RKGRKKEKKR
Ric-com_KAN2                   --*------- ---------- ---------- ---------- ----------
Ath_KAN4                       --SWGMEYAE PSSDLTLLKC *--------- ---------- ----------
Ric-com_KAN4                   --SWQMDYAE SSNELTLLKC *--------- ---------- ----------
Pop-tri_KAN4                   --SWQLDYAE TTNELTLLKC *--------- ---------- ----------
Nel-nuc_KAN4                   --SWEMDYAD PTNELTLLKC *--------- ---------- ----------
The-cac_KAN4                   --SWQMDYAE SSNELTLLKC *--------- ---------- ----------
Vit-vin_KAN4                   --SWQIDYAD QSPNELALLK C*VL*IS*IM CFQREKISSM HEICSRNFVL
Glycine_KAN4                   --NWQKDHPE SSRELTLLKC *--------- ---------- ----------
Sol-lyc_KAN4                   --SWKMGQAG SSSNDLTLLK C*-------- ---------- ----------
Med_tru_KAN4                   --NWGKDHAE SSRELSLLKC *--------- ---------- ----------
Vit-vin_KAN4                   --SWQIDYAD QSPNELALLK C*-------- ---------- ----------
Pop-tri_KAN4                   ---------- ---------- ---------- ---------- ----------
Sol_tub_KAN4                   --MSHLR*-- ---------- ---------- ---------- ----------
Pru-per_KAN4                   TAEWTVLNSL NLQMT*---- ---------- ---------- ----------
Amb-tri_KAN4                   --SWQNDYSE TTSELALLKC *--------- ---------- ----------
Fra-vesca_KAN4                 SGEWSMLNTL NLQMT*---- ---------- ---------- ----------
Mus-acu_KAN4                   --SWQMAYAE TSNEMTLLRC L*-------- ---------- ----------
Glyc_KAN4                      --NWQKDHTE SSRELTLLKC *--------- ---------- ----------
Glyc-KAN4                      --TSCKQTTR X--------- ---------- ---------- ----------
Cam-sat_KAN4                   --SWGMDYAE PSNDLTLLKC *LFNVKNYNK *VSLDCQF*H NLTC*IIWTL
Bra-rapa_KAN4                  --SWGIDHV* ---------- ---------- ---------- ----------
Bra-nap_KAN4                   --SWGIDHV* ---------- ---------- ---------- ----------
```

```
                                701
Ath_KAN1                        ---------- ---------- ---------- ---------- ----------
Amb-tric_KAN1                   ---------- ---------- ---------- ---------- ----------
Cam-sat_KAN1                    ---------- ---------- ---------- ---------- ----------
Bras-ole_KAN1-like              ---------- ---------- ---------- ---------- ----------
Bra-nap_KAN1-like               ---------- ---------- ---------- ---------- ----------
Vit-vin_KAN1                    ---------- ---------- ---------- ---------- ----------
Pru-mum_KAN1-variantX1          ---------- ---------- ---------- ---------- ----------
The-cacao_KAN1-variantX1        ---------- ---------- ---------- ---------- ----------
Ric-com_KAN1-variantX1          ---------- ---------- ---------- ---------- ----------
Pop-eup_KAN1                    ---------- ---------- ---------- ---------- ----------
Pru-per_KAN1-variantX1          ---------- ---------- ---------- ---------- ----------
Sol-tub_KAN1-like-variantX2     ---------- ---------- ---------- ---------- ----------
Nel-nuc_KAN1-variantX1          ---------- ---------- ---------- ---------- ----------
Vig-rad_KAN1                    ---------- ---------- ---------- ---------- ----------
Ath_KAN2                        ---------- ---------- ---------- ---------- ----------
Amb-tri_KAN2                    ---------- ---------- ---------- ---------- ----------
Ara-tha_GARP-like_KAN2          ---------- ---------- ---------- ---------- ----------
Cam-sat_KAN2                    ---------- ---------- ---------- ---------- ----------
Bra-nap_KAN2                    ---------- ---------- ---------- ---------- ----------
Bra-rapa_KAN2                   ---------- ---------- ---------- ---------- ----------
Cit-sin_KAN2                    ---------- ---------- ---------- ---------- ----------
Vit-vin_KAN2                    ---------- ---------- ---------- ---------- ----------
The-cacao_KAN2                  ---------- ---------- ---------- ---------- ----------
Med-tru_KAN2                    ---------- ---------- ---------- ---------- ----------
Glycine_KAN2                    ---------- ---------- ---------- ---------- ----------
Vig-rad_KAN2                    ---------- ---------- ---------- ---------- ----------
Vig-ang_KAN2                    ---------- ---------- ---------- ---------- ----------
Vit-vin_KAN2-variantX4          ---------- ---------- ---------- ---------- ----------
Pop-trichocarpa                 ---------- ---------- ---------- ---------- ----------
Ath_KAN3                        ---------- ---------- ---------- ---------- ----------
Bra-napus_KAN3                  ---------- ---------- ---------- ---------- ----------
Bra-nap_KAN3                    FPVNLRSLFX ---------- ---------- ---------- ----------
Bra-nap_KAN3                    FPVNLRSLFX ---------- ---------- ---------- ----------
Cam-sat_KAN3                    ---------- ---------- ---------- ---------- ----------
Raph-sat_KAN3                   ---------- ---------- ---------- ---------- ----------
Vig-ang_KAN2                    KKKSKRS*RT QGDKKDRKQQ KKGLREKSMA FLSELL*DPN QHIIFSW*IY
Med-tru_KAN2                    ---------- ---------- ---------- ---------- ----------
Vig-rad_KAN2                    ---------- ---------- ---------- ---------- ----------
Glyc_KAN2                       ---------- ---------- ---------- ---------- ----------
Vit-vin_KAN2                    ---------- ---------- ---------- ---------- ----------
Theo-cac_KAN2                   ---------- ---------- ---------- ---------- ----------
Pop-tri_hypothetical.protein    RAKE*QRDSK DRKQQKVKSG KGMSFISSSE IQINILYSYV NMKSN*FCML
Ric-com_KAN2                    ---------- ---------- ---------- ---------- ----------
Ath_KAN4                        ---------- ---------- ---------- ---------- ----------
Ric-com_KAN4                    ---------- ---------- ---------- ---------- ----------
Pop-tri_KAN4                    ---------- ---------- ---------- ---------- ----------
Nel-nuc_KAN4                    ---------- ---------- ---------- ---------- ----------
The-cac_KAN4                    ---------- ---------- ---------- ---------- ----------
Vit-vin_KAN4                    SK*KKGKKRR EFINGHNPLI *IX------- ---------- ----------
Glycine_KAN4                    ---------- ---------- ---------- ---------- ----------
Sol-lyc_KAN4                    ---------- ---------- ---------- ---------- ----------
Med_tru_KAN4                    ---------- ---------- ---------- ---------- ----------
Vit-vin_KAN4                    ---------- ---------- ---------- ---------- ----------
Pop-tri_KAN4                    ---------- ---------- ---------- ---------- ----------
Sol_tub_KAN4                    ---------- ---------- ---------- ---------- ----------
Pru-per_KAN4                    ---------- ---------- ---------- ---------- ----------
Amb-tri_KAN4                    ---------- ---------- ---------- ---------- ----------
Fra-vesca_KAN4                  ---------- ---------- ---------- ---------- ----------
Mus-acu_KAN4                    ---------- ---------- ---------- ---------- ----------
Glyc_KAN4                       ---------- ---------- ---------- ---------- ----------
Glyc-KAN4                       ---------- ---------- ---------- ---------- ----------
Cam-sat_KAN4                    EESLSSHMIL FQL*CSMIVF GCINKRIGLS SX-------- ----------
Bra-rapa_KAN4                   ---------- ---------- ---------- ---------- ----------
Bra-nap_KAN4                    ---------- ---------- ---------- ---------- ----------
```

```
                                 751
Ath_KAN1                         ---------- ---------- ---------- ---------- ----------
Amb-tric_KAN1                    ---------- ---------- ---------- ---------- ----------
Cam-sat_KAN1                     ---------- ---------- ---------- ---------- ----------
Bras-ole_KAN1-like               ---------- ---------- ---------- ---------- ----------
Bra-nap_KAN1-like                ---------- ---------- ---------- ---------- ----------
Vit-vin_KAN1                     ---------- ---------- ---------- ---------- ----------
Pru-mum_KAN1-variantX1           ---------- ---------- ---------- ---------- ----------
The-cacao_KAN1-variantX1         ---------- ---------- ---------- ---------- ----------
Ric-com_KAN1-variantX1           ---------- ---------- ---------- ---------- ----------
Pop-eup_KAN1                     ---------- ---------- ---------- ---------- ----------
Pru-per_KAN1-variantX1           ---------- ---------- ---------- ---------- ----------
Sol-tub_KAN1-like-variantX2      ---------- ---------- ---------- ---------- ----------
Nel-nuc_KAN1-variantX1           ---------- ---------- ---------- ---------- ----------
Vig-rad_KAN1                     ---------- ---------- ---------- ---------- ----------
Ath_KAN2                         ---------- ---------- ---------- ---------- ----------
Amb-tri_KAN2                     ---------- ---------- ---------- ---------- ----------
Ara-tha_GARP-like_KAN2           ---------- ---------- ---------- ---------- ----------
Cam-sat_KAN2                     ---------- ---------- ---------- ---------- ----------
Bra-nap_KAN2                     ---------- ---------- ---------- ---------- ----------
Bra-rapa_KAN2                    ---------- ---------- ---------- ---------- ----------
Cit-sin_KAN2                     ---------- ---------- ---------- ---------- ----------
Vit-vin_KAN2                     ---------- ---------- ---------- ---------- ----------
The-cacao_KAN2                   ---------- ---------- ---------- ---------- ----------
Med-tru_KAN2                     ---------- ---------- ---------- ---------- ----------
Glycine_KAN2                     ---------- ---------- ---------- ---------- ----------
Vig-rad_KAN2                     ---------- ---------- ---------- ---------- ----------
Vig-ang_KAN2                     ---------- ---------- ---------- ---------- ----------
Vit-vin_KAN2-variantX4           ---------- ---------- ---------- ---------- ----------
Pop-trichocarpa                  ---------- ---------- ---------- ---------- ----------
Ath_KAN3                         ---------- ---------- ---------- ---------- ----------
Bra-napus_KAN3                   ---------- ---------- ---------- ---------- ----------
Bra-nap_KAN3                     ---------- ---------- ---------- ---------- ----------
Bra-nap_KAN3                     ---------- ---------- ---------- ---------- ----------
Cam-sat_KAN3                     ---------- ---------- ---------- ---------- ----------
Raph-sat_KAN3                    ---------- ---------- ---------- ---------- ----------
Vig-ang_KAN2                     EI*LICMFLQ SERKKET*ES ERE*ESERER ERERELS*KY EVILGNCNGA
Med-tru_KAN2                     ---------- ---------- ---------- ---------- ----------
Vig-rad_KAN2                     ---------- ---------- ---------- ---------- ----------
Glyc_KAN2                        ---------- ---------- ---------- ---------- ----------
Vit-vin_KAN2                     ---------- ---------- ---------- ---------- ----------
Theo-cac_KAN2                    ---------- ---------- ---------- ---------- ----------
Pop-tri_hypothetical.protein     LHSRRKRERE RERERPLERD GEREL*PSQS IKRSLVIVTE QFTLI*NIFI
Ric-com_KAN2                     ---------- ---------- ---------- ---------- ----------
Ath_KAN4                         ---------- ---------- ---------- ---------- ----------
Ric-com_KAN4                     ---------- ---------- ---------- ---------- ----------
Pop-tri_KAN4                     ---------- ---------- ---------- ---------- ----------
Nel-nuc_KAN4                     ---------- ---------- ---------- ---------- ----------
The-cac_KAN4                     ---------- ---------- ---------- ---------- ----------
Vit-vin_KAN4                     ---------- ---------- ---------- ---------- ----------
Glycine_KAN4                     ---------- ---------- ---------- ---------- ----------
Sol-lyc_KAN4                     ---------- ---------- ---------- ---------- ----------
Med_tru_KAN4                     ---------- ---------- ---------- ---------- ----------
Vit-vin_KAN4                     ---------- ---------- ---------- ---------- ----------
Pop-tri_KAN4                     ---------- ---------- ---------- ---------- ----------
Sol_tub_KAN4                     ---------- ---------- ---------- ---------- ----------
Pru-per_KAN4                     ---------- ---------- ---------- ---------- ----------
Amb-tri_KAN4                     ---------- ---------- ---------- ---------- ----------
Fra-vesca_KAN4                   ---------- ---------- ---------- ---------- ----------
Mus-acu_KAN4                     ---------- ---------- ---------- ---------- ----------
Glyc_KAN4                        ---------- ---------- ---------- ---------- ----------
Glyc-KAN4                        ---------- ---------- ---------- ---------- ----------
Cam-sat_KAN4                     ---------- ---------- ---------- ---------- ----------
Bra-rapa_KAN4                    ---------- ---------- ---------- ---------- ----------
Bra-nap_KAN4                     ---------- ---------- ---------- ---------- ----------
```

```
                                801
Ath_KAN1                          ---------- ---------- ---------- ---------- ----------
Amb-tric_KAN1                     ---------- ---------- ---------- ---------- ----------
Cam-sat_KAN1                      ---------- ---------- ---------- ---------- ----------
Bras-ole_KAN1-like                ---------- ---------- ---------- ---------- ----------
Bra-nap_KAN1-like                 ---------- ---------- ---------- ---------- ----------
Vit-vin_KAN1                      ---------- ---------- ---------- ---------- ----------
Pru-mum_KAN1-variantX1            ---------- ---------- ---------- ---------- ----------
The-cacao_KAN1-variantX1          ---------- ---------- ---------- ---------- ----------
Ric-com_KAN1-variantX1            ---------- ---------- ---------- ---------- ----------
Pop-eup_KAN1                      ---------- ---------- ---------- ---------- ----------
Pru-per_KAN1-variantX1            ---------- ---------- ---------- ---------- ----------
Sol-tub_KAN1-like-variantX2       ---------- ---------- ---------- ---------- ----------
Nel-nuc_KAN1-variantX1            ---------- ---------- ---------- ---------- ----------
Vig-rad_KAN1                      ---------- ---------- ---------- ---------- ----------
Ath_KAN2                          ---------- ---------- ---------- ---------- ----------
Amb-tri_KAN2                      ---------- ---------- ---------- ---------- ----------
Ara-tha_GARP-like_KAN2            ---------- ---------- ---------- ---------- ----------
Cam-sat_KAN2                      ---------- ---------- ---------- ---------- ----------
Bra-nap_KAN2                      ---------- ---------- ---------- ---------- ----------
Bra-rapa_KAN2                     ---------- ---------- ---------- ---------- ----------
Cit-sin_KAN2                      ---------- ---------- ---------- ---------- ----------
Vit-vin_KAN2                      ---------- ---------- ---------- ---------- ----------
The-cacao_KAN2                    ---------- ---------- ---------- ---------- ----------
Med-tru_KAN2                      ---------- ---------- ---------- ---------- ----------
Glycine_KAN2                      ---------- ---------- ---------- ---------- ----------
Vig-rad_KAN2                      ---------- ---------- ---------- ---------- ----------
Vig-ang_KAN2                      ---------- ---------- ---------- ---------- ----------
Vit-vin_KAN2-variantX4            ---------- ---------- ---------- ---------- ----------
Pop-trichocarpa                   ---------- ---------- ---------- ---------- ----------
Ath_KAN3                          ---------- ---------- ---------- ---------- ----------
Bra-napus_KAN3                    ---------- ---------- ---------- ---------- ----------
Bra-nap_KAN3                      ---------- ---------- ---------- ---------- ----------
Bra-nap_KAN3                      ---------- ---------- ---------- ---------- ----------
Cam-sat_KAN3                      ---------- ---------- ---------- ---------- ----------
Raph-sat_KAN3                     ---------- ---------- ---------- ---------- ----------
Vig-ang_KAN2                      LYIYEIFRYF HQRSWVPNPI FNSTPRHMLY TLGTKERIEN MDLGKD*KI*
Med-tru_KAN2                      ---------- ---------- ---------- ---------- ----------
Vig-rad_KAN2                      ---------- ---------- ---------- ---------- ----------
Glyc_KAN2                         ---------- ---------- ---------- ---------- ----------
Vit-vin_KAN2                      ---------- ---------- ---------- ---------- ----------
Theo-cac_KAN2                     ---------- ---------- ---------- ---------- ----------
Pop-tri_hypothetical.protein      FLX------- ---------- ---------- ---------- ----------
Ric-com_KAN2                      ---------- ---------- ---------- ---------- ----------
Ath_KAN4                          ---------- ---------- ---------- ---------- ----------
Ric-com_KAN4                      ---------- ---------- ---------- ---------- ----------
Pop-tri_KAN4                      ---------- ---------- ---------- ---------- ----------
Nel-nuc_KAN4                      ---------- ---------- ---------- ---------- ----------
The-cac_KAN4                      ---------- ---------- ---------- ---------- ----------
Vit-vin_KAN4                      ---------- ---------- ---------- ---------- ----------
Glycine_KAN4                      ---------- ---------- ---------- ---------- ----------
Sol-lyc_KAN4                      ---------- ---------- ---------- ---------- ----------
Med_tru_KAN4                      ---------- ---------- ---------- ---------- ----------
Vit-vin_KAN4                      ---------- ---------- ---------- ---------- ----------
Pop-tri_KAN4                      ---------- ---------- ---------- ---------- ----------
Sol_tub_KAN4                      ---------- ---------- ---------- ---------- ----------
Pru-per_KAN4                      ---------- ---------- ---------- ---------- ----------
Amb-tri_KAN4                      ---------- ---------- ---------- ---------- ----------
Fra-vesca_KAN4                    ---------- ---------- ---------- ---------- ----------
Mus-acu_KAN4                      ---------- ---------- ---------- ---------- ----------
Glyc_KAN4                         ---------- ---------- ---------- ---------- ----------
Glyc-KAN4                         ---------- ---------- ---------- ---------- ----------
Cam-sat_KAN4                      ---------- ---------- ---------- ---------- ----------
Bra-rapa_KAN4                     ---------- ---------- ---------- ---------- ----------
Bra-nap_KAN4                      ---------- ---------- ---------- ---------- ----------
```

	851
Ath_KAN1	--------
Amb-tric_KAN1	--------
Cam-sat_KAN1	--------
Bras-ole_KAN1-like	--------
Bra-nap_KAN1-like	--------
Vit-vin_KAN1	--------
Pru-mum_KAN1-variantX1	--------
The-cacao_KAN1-variantX1	--------
Ric-com_KAN1-variantX1	--------
Pop-eup_KAN1	--------
Pru-per_KAN1-variantX1	--------
Sol-tub_KAN1-like-variantX2	--------
Nel-nuc_KAN1-variantX1	--------
Vig-rad_KAN1	--------
Ath_KAN2	--------
Amb-tri_KAN2	--------
Ara-tha_GARP-like_KAN2	--------
Cam-sat_KAN2	--------
Bra-nap_KAN2	--------
Bra-rapa_KAN2	--------
Cit-sin_KAN2	--------
Vit-vin_KAN2	--------
The-cacao_KAN2	--------
Med-tru_KAN2	--------
Glycine_KAN2	--------
Vig-rad_KAN2	--------
Vig-ang_KAN2	--------
Vit-vin_KAN2-variantX4	--------
Pop-trichocarpa	--------
Ath_KAN3	--------
Bra-napus_KAN3	--------
Bra-nap_KAN3	--------
Bra-nap_KAN3	--------
Cam-sat_KAN3	--------
Raph-sat_KAN3	--------
Vig-ang_KAN2	IKCQKEYQ
Med-tru_KAN2	--------
Vig-rad_KAN2	--------
Glyc_KAN2	--------
Vit-vin_KAN2	--------
Theo-cac_KAN2	--------
Pop-tri_hypothetical.protein	--------
Ric-com_KAN2	--------
Ath_KAN4	--------
Ric-com_KAN4	--------
Pop-tri_KAN4	--------
Nel-nuc_KAN4	--------
The-cac_KAN4	--------
Vit-vin_KAN4	--------
Glycine_KAN4	--------
Sol-lyc_KAN4	--------
Med_tru_KAN4	--------
Vit-vin_KAN4	--------
Pop-tri_KAN4	--------
Sol_tub_KAN4	--------
Pru-per_KAN4	--------
Amb-tri_KAN4	--------
Fra-vesca_KAN4	--------
Mus-acu_KAN4	--------
Glyc_KAN4	--------
Glyc-KAN4	--------
Cam-sat_KAN4	--------
Bra-rapa_KAN4	--------
Bra-nap_KAN4	--------
