# Supplementary material for: Evidence for the Extensive Conservation of Mechanisms of Ovule Integument Development Since the Most Recent Common Ancestor of Living Angiosperms
Source: Front Plant Sci. 2018 Sep 19;9:1352. doi: 10.3389/fpls.2018.01352 (PMC6156155; doi:10.3389/fpls.2018.01352)
Supplement: FIGURE S4 — Alignment of angiosperm KANADI family proteins used to produce the phylogeny shown in Supplementary Figure 3. Sites used in phylogenetic analysis are underlined. [file Image_4.pdf]

```
                              1
AmTr_v1.0_scaffold00148.15    ---------- ---------- -------MAV AVHK------ ----------
AmTr_v1.0_scaffold00056.60    ---------- ---------- -------MAV TACK------ ----------
AmTr_v1.0_scaffold00155.45    ---------- ---------- -------MAL VLQK------ ----------
Ara_thaPHABULOSA              ---------- ---------- --------MM MVHS-MS--- ----RDMMNR
Ara_thaATHB15                 ---------- ---------- -------MAM SCKD------ ----------
Ara-thaPHAVOLUTA              ---------- ---------- --------MM AHHSMDD--- ----RD----
Ara_thaATHB8                  ---------- ---------- -------MG- ---------- ---------G
Ara_thaREVOLUTA               ---------- ---------- -------MEM AVAN--H--- ----RERSSD
Vit_vinHOX32-like             ---------- ---------- -------MAL SMHK------ ----------
The_cacXP_007022870.1         ---------- ---------- ---------- ---------- ----------
Ory_satHOX32                  ---------- ---------- -------MAA AMVAAVHGVG R---QDRSSP
Pru_perppa001343mg            ---------- ---------- -------MAL VMN------- ----RD--SP
Bra_disHOX32-like             ---------- ---------- -------MAG AMVAGLHGGG RDRDRDRSSP
Pop_triPHABULOSA              ---------- ---------- -------MAL SIHS------ ----------
Pop_triPHABULOSAb             ---------- ---------- -------MAL SMHS------ ----------
Fra_vesHOX32-like             ---------- ---------- -------MAL VMHK------ ---------E
Gly_maxATHB-14-like           ---------- ---------- -------MAL SMHK------ ----------
Gly_maxREVOLUTA-like          ---------- ---------- -------MAM VVAQ--H--- ----RESSSS
Gly_maxREVOLUTA-likeb         ---------- ---------- -------MAM AVAQ--H--- ----RESSSS
Pru_perREV_IFL1               ---------- ---------- -------MAM AVAH--H--- ----RESSSG
Ory_satHOX33                  ---------- ---------- ----MAAAAV GGRG-ER--- ----LSSSSP
Sol_tubATHB-14-like           ---------- ---------- -------MAL CLQR------ -----GGGGE
The_cacXP_007022872.1         ---------- ---------- -------MAL SMHK------ ---------D
Ory_satHOX33-like             ---------- ---------- -------MAA AA-------- ----------
Sol_lycREVOLUTA-like          ---------- ---------- -------MAM VAQQ--H--- ----RE-SSS
Sol_lycHOX32-like             ---------- ---------- -------MAL CLQR------ ------GEGE
Gly_maxATHB-14-likeb          ---------- ---------- -------MAL CMQ------- ----RD--SA
Pop_triXP_006378973.1         ---------- ---------- -------MAM AVAP-QH--- ----RESSGS
Ory_satACA64837.1             ---------- ---------- ----MAAAAV GGRG-ER--- ----LSSSSP
Vit_vin013992                 ---------- ---------- -------MAM AIAQ-QH--- ----RESSSS
Ory_satOsJ_11861              ---------- ---------- ---------- ---------- ----------
Sol_tubREVOLUTA-like          ---------- ---------- -------MAM VAQQ--H--- ----RESSSG
Gly_maxATHB-14-likec          ---------- ---------- -------MAL CMQS-QQ--- ----RD--SA
The_cacXP_007024277.1         ---------- ---------- -------MAM SCKD------ ----------
Vit_vinATHB-15-like           ---------- ---------- -------MAM SCKD------ ----------
Ric_comXP_002515977.1         ---------- ---------- -------MAM SCKD------ ----------
The_cacXP_007016751.1         ---------- ---------- -------MAM AVAQ--H--- ----RESSSG
Sol_tubHOX32-like             ---------- ---------- -------MAL CLQR------ ------GEGE
Sol_lycATHB-14-like           ---------- ---------- ---------- ---------- ----------
Sol_tubATHB-14-like_X1        ---------- ---------- ---------- ---------- ----------
Med_truATHB-14                ---------- ---------- -------MAL SMH------- ----------
Zea_mayNP_001142394.1         ---------- ---------- -------MAA VASR------ ------ERLS
Vit_vinREVOLUTA               ---------- ---------- -------MAM AIAQ-QH--- ----RESSSS
Ory_braHOX32-like             ---------- ---------- -------MEV PAYQ------ ----------
Ric_comXP_002529946.1         ---------- ---------- -------MAM AMVQ--H--- ----RETSSG
Tha_cacXP_007016749.1         ---------- ---------- -------MAM AVAQ--H--- ----RESSSG
Fra_vesREVOLUTA-like          ---------- ---------- -------MAM AVAHHQH--- ----RESSSG
Zea_mayNP_001146215.1         ---------- ---------- -------MAS SRG------- ----RLRLSP
The_cacXP_007012152.1         MLIAAEGTGK TKAKATKNLE WIFEVSPVIR SCPKVPEENE VMMAVTSSCK
Pru_perppa001386mg            ---------- ---------- -------MMA VTSA------ -------CKD
Pop_triAAX19057.1             ---------- ---------- ---------- ---------- ----------
Pop_triXP_002309538.2         ---------- ---------- ---------- ---------- ----------
Fra_vesATHB-8-like            ---------- ---------- -------MAV TSSS-SC--- ----KE-LSG
Pop_triXP_002324794.1         ---------- ---------- ---------- ---------- ----------
Vit_vin009744                 ---------- ---------- ---------- ---------- ----------
Gly_maxATHB-8-like            ---------- ---------- -------MMT VSSA--C--- ----KD----
Sol_lycATHB-15-like           ---------- ---------- -------MMA VTSS------ ----------
Gly_maxATHB-8-likeb           ---------- ---------- -------MMA VSSA--C--- ----KD----
Med_truXP_003603630.1         ---------- ---------- -------MAV SSGC------ ----KD----
```

```
                             51
AmTr_v1.0_scaffold00148.15   -DSSKHM-DS SKYVRYTAEQ VEALERVYNE CPKPSSLRRQ QLIRECPILC
AmTr_v1.0_scaffold00056.60   -EGKPGM-DP GKYVRYTPEQ VEALERLYHE CPKPSSIRRQ QLIRECPILS
AmTr_v1.0_scaffold00155.45   -DSNKHM-DT SKYVRYTPEQ VEALERVYSE CPKPSSLRRQ QLIRECPILS
Ara_thaPHABULOSA             ESPDKGL-DS GKYVRYTPEQ VEALERVYTE CPKPSSLRRQ QLIRECPILS
Ara_thaATHB15                -GKLGCL-DN GKYVRYTPEQ VEALERLYHD CPKPSSIRRQ QLIRECPILS
Ara-thaPHAVOLUTA             -SPDKGF-DS GKYVRYTPEQ VEALERVYAE CPKPSSLRRQ QLIRECPILC
Ara_thaATHB8                 GSNNSHNMDN GKYVRYTPEQ VEALERLYND CPKPSSMRRQ QLIRECPILS
Ara_thaREVOLUTA              -SMNRHLDSS GKYVRYTAEQ VEALERVYAE CPKPSSLRRQ QLIRECSILA
Vit_vinHOX32-like            -ESKQQM-DS SKYVRYTPEQ VEALERVYSE CPKPSSMRRQ QLIRECPILS
The_cacXP_007022870.1        ------M-DS SKYVRYTPEQ VEALERVYSE CPKPSSLRRQ QLIRECPILS
Ory_satHOX32                 GGGGAPQVDT GKYVRYTPEQ VEALERVYGE CPKPSSLRRQ QLIRECPILS
Pru_perppa001343mg           GSSSKQM-DS SKYVRYTPEQ VEALERVYSE CPKPSSLRRQ QLIRECPILS
Bra_disHOX32-like            GGGGAPQVDT GKYVRYTPEQ VDALERVYSE CPKPSSLRRQ QLIRECPILS
Pop_triPHABULOSA             --KDKHM-DS SKYVRYTPEQ VEALERVYTE CPKPSSLRRQ QLIRECPILS
Pop_triPHABULOSAb            --KDKQM-DS SKYVRYTPEQ VEALERVYTE CPKPSSLRRQ QLIRECPILC
Fra_vesHOX32-like            KHNNNQM-DS SKYVRYTPEQ VEALERVYSE CPKPSSLRRQ QLIRECPILS
Gly_maxATHB-14-like          DSANNQM-DS SKYVRYTPEQ VEALERVYAE CPKPSSLRRQ QLIRECPILS
Gly_maxREVOLUTA-like         GSIDKHL-DS GKYVRYTAEQ VEALERVYAE CPKPSSLRRQ QLIRECPILS
Gly_maxREVOLUTA-likeb        GSIDKHL-DS GKYVRYTAEQ VEALERVYAE CPKPSSLRRQ QLIRECPILS
Pru_perREV_IFL1              SSINKHL-DA GKYVRYTSEQ VEALERVYAE CPKPSSLRRQ QLIRECPILS
Ory_satHOX33                 TAAAPQV-DA GKYVRYTPEQ VEALERVYTE CPKPSSLRRQ QLIRECPILS
Sol_tubATHB-14-like          SGSKNDM-DN GKYVRYTPEQ VEALERVYAE CPKPTSLRRQ QLIRECPILS
The_cacXP_007022872.1        SSNNKQM-DS SKYVRYTPEQ VEALERVYSE CPKPSSLRRQ QLIRECPILS
Ory_satHOX33-like            ---------- -----YTPEQ VEALERVYTE CPKPSSLRRQ QLIRECPILS
Sol_lycREVOLUTA-like         GSITKHLDSS GKYVRYTAEQ VEALERVYAE CPKPSSLRRQ QLIRECHILS
Sol_lycHOX32-like            SGSKNDM-DN GKYVRYTPEQ VEALERVYAE CPKPTSLRRQ QLIRESPILS
Gly_maxATHB-14-likeb         SNKQQLM-DC GKYVRYTPEQ VEALERVYIE CPKPSSSRRQ QIIRECPLLA
Pop_triXP_006378973.1        GSLNKHLTDN GKYVRYTSEQ VEALERVYAE CPKPSSLRRQ QLIRECPILA
Ory_satACA64837.1            TAAAPQV-DA GKYVRYTPEQ VEALERVYTE CPKPSSLRRQ QLIRECPILS
Vit_vin013992                GSINKHL-DS GKYVRYTAEQ VEALERVYLE CPKPSSLRRQ QLIRECPILS
Ory_satOsJ_11861             ---------- ---MPYHP-- --------IT C--------- TLIRECPILS
Sol_tubREVOLUTA-like         -SITKHLDSS GKYVRYTAEQ VEALERVYAE CPKPSSLRRQ QLIRECHILS
Gly_maxATHB-14-likec         SNK-LLM-DC GKYVRYTPEQ VEALERVYVE CPKPSSSRRQ QIIRECPLLA
The_cacXP_007024277.1        -GKLGNL-DN GKYVRYTPEQ VEALERLYHE CPKPSSIRRQ QLIRECPILS
Vit_vinATHB-15-like          ---GKGIMDN GKYVRYTPEQ VEALERLYHE CPKPSSIRRQ QLIRECPILS
Ric_comXP_002515977.1        GKQPANL-DN GKYVRYTPEQ VEALERLYHE CPKPSSIRRQ QFIRECPILS
The_cacXP_007016751.1        SSINKHL-DA GKYVRYTAEQ VEALERVYAE CPKPSSLRRQ QLIRECPILS
Sol_tubHOX32-like            SGSKNDM-DN GKYVRYTPEQ VEALERVYAE CPKPTSLRRQ QLIRESPILS
Sol_lycATHB-14-like          ------M-DS SKYVRYTPEQ VEALERVYAE CPKPTSLKRH QLIRECSILS
Sol_tubATHB-14-like_X1       ------M-DS SKYVRYTPEQ VEALERVYAE CPKPTSLKRH QLIRECSILS
Med_truATHB-14               ----KEM-DA GKYVRYTPEQ VEALERVYSE CPKPSSLRRQ QLIRECPILS
Zea_mayNP_001142394.1        PGAAPQV-DA GKYVRYTPEQ VEALERVYSE CPKPSSLRRQ HLIRDCPILR
Vit_vinREVOLUTA              GSINKHL-DS GKYVRYTAEQ VEALERVYLE CPKPSSLRRQ QLIRECPILS
Ory_braHOX32-like            ---------- -----FHAMA M--------- ---------- -----CSWDA
Ric_comXP_002529946.1        -SINKHLTDS GKYVRYTAEQ VEALERVYSE CPKPSSLRRQ QLIRECPILS
Tha_cacXP_007016749.1        SSINKHL-DA GKYVRYTAEQ VEALERVYAE CPKPSSLRRQ QLIRECPILS
Fra_vesREVOLUTA-like         SSINKHLNDA GKYVRYTSEQ VEALERVYAE CPKPSSLRRQ QLIRDCPILS
Zea_mayNP_001146215.1        RAAAPQL-DA GKYVRYTAEQ VDALELAYGE CPKPSSLRRQ QLIRDCAVLT
The_cacXP_007012152.1        EGNKIAM-DN GKYVRYTPEQ VDALERLYHE CPKPSSMRRQ QLIRECPILA
Pru_perppa001386mg           GGMKMQM-DN GKYVRYTPEQ VEALERLYHE CPKPSSMRRQ QLIRECPILS
Pop_triAAX19057.1            ------M-DN GKYVRYTPEQ VEALERLYHE CPKPSSMRRQ QLIRECPILS
Pop_triXP_002309538.2        ------M-DN GKYVRYTPEQ VEALERLYHE CPKPSSMRRQ QLIRECPILS
Fra_vesATHB-8-like           GGMKMQM-DN GKYVRYTPEQ VEALERLYHE CPKPSSMRRQ QLIRECPILS
Pop_triXP_002324794.1        ------M-DN GKYVRYTPEQ VEALERLYHE CPKPTSTRRQ QLIRECPILS
Vit_vin009744                ----MAL-DN GKYVRYTPEQ VEALERLYHD CPKPSSLRRQ QLIRECPILS
Gly_maxATHB-8-like           -GSKVAL-DN GKYVRYTPEQ VEALERLYHE CPKPSSLRRQ QLIRECPILS
Sol_lycATHB-15-like          -CKDKFGMDS GKYVRYTPEQ VEALERLYHE CPKPSSLRRQ QLIRECPILS
Gly_maxATHB-8-likeb          -GSKVAL-DN GKYVRYTPEQ VEALERLYHE CPKPSSLRRQ QLIRECPILS
Med_truXP_003603630.1        -GSKIAM-DN GKYVRYTPEQ VEALERLYHE CPKPTSLRRQ QLIRECPILS
```

	101				
AmTr_v1.0_scaffold00148.15	NIEPKQIKVW	FQNRRCREKQ	RKEASRLQTV	NRKLTAMNKL	LMEENDRLQK
AmTr_v1.0_scaffold00056.60	NIEPKQIKVW	FQNRRCREKQ	RKEASRLQAV	NRKLTAMNKL	LMEENDRLQK
AmTr_v1.0_scaffold00155.45	NIEPKQIKVW	FQNRRCREKQ	RKEASRLQTV	NRKLTAMNKL	LMEENDRLQK
Ara_thaPHABULOSA	NIEPKQIKVW	FQNRRCREKQ	RKEAARLQTV	NRKLNAMNKL	LMEENDRLQK
Ara_thaATHB15	NIEPKQIKVW	FQNRRCREKQ	RKEASRLQAV	NRKLTAMNKL	LMEENDRLQK
Ara-thaPHAVOLUTA	NIEPRQIKVW	FQNRRCREKQ	RKESARLQTV	NRKLSAMNKL	LMEENDRLQK
Ara_thaATHB8	NIEPKQIKVW	FQNRRCREKQ	RKEASRLQAV	NRKLTAMNKL	LMEENDRLQK
Ara_thaREVOLUTA	NIEPKQIKVW	FQNRRCRDKQ	RKEASRLQSV	NRKLSAMNKL	LMEENDRLQK
Vit_vinHOX32-like	NIEPKQIKVW	FQNRRCREKQ	RKEASRLQTV	NRKLTAMNKL	LMEENDRLQK
The_cacXP_007022870.1	NIEPKQIKVW	FQNRRCREKQ	RKEASRLQTV	NRKLSAMNKL	LMEENDRLQK
Ory_satHOX32	NIEPKQIKVW	FQNRRCREKQ	RKEASRLQTV	NRKLTAMNKL	LMEENDRLQK
Pru_perppa001343mg	NIEPKQIKVW	FQNRRCREKQ	RKESSRLQTV	NRKLTAMNKL	LMEENDRLQK
Bra_disHOX32-like	NIEPKQIKVW	FQNRRCREKQ	RKEASRLQTV	NRKLSAMNKL	LMEENDRLQK
Pop_triPHABULOSA	NIEPKQIKVW	FQNRRCREKQ	RKEASRLQTV	NRKLTAMNKL	LMEENDRLQK
Pop_triPHABULOSAb	NIEPKQIKVW	FQNRRCREKQ	RKESSRLQTV	NRKLTAMNKL	LMEENDRLQK
Fra_vesHOX32-like	NIEPKQIKVW	FQNRRCREKQ	RKESSRLTTV	NRKLSAMNKL	LMEENDRLQK
Gly_maxATHB-14-like	NIEPKQIKVW	FQNRRCREKQ	RKEASRLQTV	NRKLTAMNKL	LMEENDRLQK
Gly_maxREVOLUTA-like	NIEPKQIKVW	FQNRRCREKQ	RKEASRLQTV	NRKLTAMNKL	LMEENDRLQK
Gly_maxREVOLUTA-likeb	NIEPKQIKVW	FQNRRCREKQ	RKEASRLQTV	NRKLTAMNKL	LMEENDRLQK
Pru_perREV_IFL1	NIEPRQIKVW	FQNRRCREKQ	RKESSRLQTV	NRKLTAMNKL	LMEENDRLQK
Ory_satHOX33	NIEPKQIKVW	FQNRRCREKQ	RKEASRLQTV	NRKLNAMNKL	LMEENDRLQK
Sol_tubATHB-14-like	NIEPKQIKVW	FQNRRCREKQ	RKEASRLQTV	NRKLSAMNKL	LMEENDRLQK
The_cacXP_007022872.1	NIEPKQIKVW	FQNRRCREKQ	RKEASRLQTV	NRKLSAMNKL	LMEENDRLQK
Ory_satHOX33-like	NIEPKQIKVW	FQNRRCREKQ	RKEASRLQTV	NRKLNAMNKL	LMEENDRLQK
Sol_lycREVOLUTA-like	NIEPKQIKVW	FQNRRCREKQ	RKESSRLQTV	NRKLSAMNKL	LMEENDRLQK
Sol_lycHOX32-like	NIEPKQIKVW	FQNRRCREKQ	RKEASRLQTV	NRKLSAMNKL	LMEENDRLQK
Gly_maxATHB-14-likeb	NIETKQIKVW	FQNRRCREKQ	RKEASRLQTV	NRKLSAMNKL	LMEENDRLQK
Pop_triXP_006378973.1	NIEPKQIKVW	FQNRRCREKQ	RKESSRLQTV	NRKLTAMNKL	LMEENDRLQK
Ory_satACA64837.1	NIEPKQIKVW	FQNRRCREKQ	RKEASRLQTV	NRKLNAMNKL	LMEENDRLQK
Vit_vin013992	NIEPKQIKVW	FQNRRCREKQ	RKEASRLQTV	NRKLTAMNKL	LMEENDRLQK
Ory_satOsJ_11861	NIEPKQIKVW	FQNRRCREKQ	RKEASRLQTV	NRKLTAMNKL	LMEENDRLQK
Sol_tubREVOLUTA-like	NIEPKQIKVW	FQNRRCREKQ	RKESSRLQTV	NRKLSAMNKL	LMEENDRLQK
Gly_maxATHB-14-likec	NIETKQIKVW	FQNRRCREKQ	RKEASRLQTV	NRKLSSMNKL	LMEENDRLQK
The_cacXP_007024277.1	NIEPKQIKVW	FQNRRCREKQ	RKEASRLQAV	NRKLTAMNKL	LMEENDRLQK
Vit_vinATHB-15-like	NIEPKQIKVW	FQNRRCREKQ	RKEASRLQAV	NRKLTAMNKL	LMEENDRLQK
Ric_comXP_002515977.1	NIEPKQIKVW	FQNRRCREKQ	RKEASRLQAV	NRKLTAMNKL	LMEENDRLQK
The_cacXP_007016751.1	NIEPKQIKVW	FQNRRCREKQ	RKEASRLQTV	NRKLTAMNKL	LMEENDRLQK
Sol_tubHOX32-like	NIEPKQIKVW	FQNRRCREKQ	RKEASRLQTV	NRKLSAMNKL	LMEENDRLQK
Sol_lycATHB-14-like	NIDPKQIKVW	FQNRRCREKQ	RKEASHLQTV	NRKLTAMNKL	LMEENDRLQK
Sol_tubATHB-14-like_X1	NIDPKQIKVW	FQNRRCREKQ	RKEASHLQTV	NRKLTAMNKL	LMEENDRLQK
Med_truATHB-14	NIEPKQIKVW	FQNR------	----------	----SFLHLL	VIV----VTK
Zea_mayNP_001142394.1	NIEPKQIKVW	FQNRRCREKQ	RKESSRLQTV	NRKLSAMNKL	LMEENDRLQK
Vit_vinREVOLUTA	NIEPKQIKVW	FQNRRCREKQ	RKEASRLQTV	NRKLTAMNKL	LMEENDRLQK
Ory_braHOX32-like	FYLTRLMLTW	DAICLCREKQ	RKEASRLQTV	NRKLTAMNKL	LMEENDRLQK
Ric_comXP_002529946.1	NIEPKQIKVW	FQNRRCREKQ	RKESSRLQTV	NRKLTAMNKL	LMEENDRLQK
Tha_cacXP_007016749.1	NIEPKQIKVW	FQNRRCREKQ	RKEASRLQTV	NRKLTAMNKL	LMEENDRLQK
Fra_vesREVOLUTA-like	NIEPKQIKVW	FQNRRCREKQ	RKESSRLQTV	NRKLSAMNKL	LMEENDRLQK
Zea_mayNP_001146215.1	NVEPRQIKVW	FQNRRCREKQ	RRESSRLQTV	NRKLGAMNKL	LMEENDRLQK
The_cacXP_007012152.1	NIEPKQIKVW	FQNRRCREKQ	RKEASRLQAV	NRKLTAMNKL	LMEENDRLQK
Pru_perppa001386mg	NIEPKQIKVW	FQNRRCREKQ	RKEASRLQTV	NRKLTAMNKL	LMEENDRLQK
Pop_triAAX19057.1	HIEPKQIKVW	FQNRRCREKQ	RKEASRLQAV	NRKLTAMNKL	LMEENDRLQK
Pop_triXP_002309538.2	HIEPKQIKVW	FQNRRCREKQ	RKEASRLQAV	NRKLTAMNKL	LMEENDRLQK
Fra_vesATHB-8-like	NIEPKQIKVW	FQNRRCREKQ	RKEASRLQTV	NRKLTAMNKL	LMEENDRLQK
Pop_triXP_002324794.1	HIEPKQIKVW	FQNRRCREKQ	RKEASRLQAV	NRKLTAMNKL	LMEENDRLQK
Vit_vin009744	NIEPKQIKVW	FQNRRCREKQ	RKEASRLQAV	NRKLTAMNKL	LMEENDRLQK
Gly_maxATHB-8-like	NIEPKQIKVW	FQNRRCREKQ	RKEASRLQAV	NRKLTAMNKL	LMEENDRLQK
Sol_lycATHB-15-like	NIEPKQIKVW	FQNRRCREKQ	RKESSRLQGV	NRKLTAMNKL	LMEENDRLQK
Gly_maxATHB-8-likeb	NIEPKQIKVW	FQNRRCREKQ	RKEASRLQAV	NRKLTAMNKL	LMEENDRLQK
Med_truXP_003603630.1	HIEPKQIKVW	FQNRRCREKQ	RKEAGRLQAV	NRKLTAMNKL	LMEENDRLQK

```
                              151
AmTr_v1.0_scaffold00148.15    QVAQLVYENG YMRQQLQN-- -VSVATTD-- TSCESVVTSG PQQQ--NPTP
AmTr_v1.0_scaffold00056.60    QVSQLVYENG YFRQQTQN-- -VAITTTD-- TSCESVVTSG QH----HLTP
AmTr_v1.0_scaffold00155.45    QVSQLVYENG YMKQQLQT-- -ASVATTD-- TSCESVVTSG QHQQL-NPTP
Ara_thaPHABULOSA              QVSNLVYENG HMKHQLHT-- -ASGTTTD-- NSCESVVVSG QQHQQQNPNP
Ara_thaATHB15                 QVSQLVHENS YFRQHTPN-- -PSLPAKD-- TSCESVVTSG QH----QLAS
Ara-thaPHAVOLUTA              QVSNLVYENG FMKHRIHT-- -ASGTTTD-- NSCESVVVSG QQRQQQNPTH
Ara_thaATHB8                  QVSHLVYENS YFRQHPQN-- QGNLATTD-- TSCESVVTSG QH----HLTP
Ara_thaREVOLUTA               QVSQLVCENG YMKQQLTT-- ----VVND-- PSCESVVT-- --------TP
Vit_vinHOX32-like             QVSQLVYENG YMRQQLQS-- -ASTATTD-- TSCESVVMSG QHQQQQNPTP
The_cacXP_007022870.1         QVSQLVYENG YMRQQLQT-- -GSATTTD-N NSCESVVMSG QHQQQQNPTP
Ory_satHOX32                  QVSRLVYENG YMRQQLHN-- -PSVATTD-- TSCESVVTSG QHHQQQNPAA
Pru_perppa001343mg            QVSHLVYENG FMRQQLHS-- -ASGTTTD-- NSCESVVMSG QHQQQQNPTP
Bra_disHOX32-like             QVSRLVYENG YMRTHLSN-- -PSVATTD-- TSCESVVTSG QHQQQQNPAV
Pop_triPHABULOSA              QVSHLVYENG FMRQQIQT-- -ASATTTD-- NSCESVVMSG QHQQQQNPTP
Pop_triPHABULOSAb             QVSHLVYENG YMRQQIQT-- -ASATATD-- NSCESVVMSG QHQQQQNPTP
Fra_vesHOX32-like             QVSHLVYENG FMKNKLHS-- -ASGTTTD-- TSCESVVMNG QHQQQHNPTP
Gly_maxATHB-14-like           QVSHLVYENG YMKQQIHT-- ASAGTTTD-- NSCESVVMSG QNQQQ-NPTP
Gly_maxREVOLUTA-like          QVSQLVCENG FMRQQLHT-- -PSATTTD-- ASCDSVVT-- --------TP
Gly_maxREVOLUTA-likeb         QVSQLVCENG FMRQQLHT-- -PSAATTD-- ASCDSVVT-- --------TP
Pru_perREV_IFL1               QVSQLVCENG FMRQQLHT-- ---APTTD-- ASCDSVVT-- --------TP
Ory_satHOX33                  QVSRLVYENG YMRTQLHN-- -PSAATTD-- TSCESVVTSG QHHQQQNPAV
Sol_tubATHB-14-like           QVSQLVYENG YMKQQINT-- -VSSTTTD-- TSCESVVVSG QQQRK-NPTP
The_cacXP_007022872.1         QVSQLVYENG YMRQQLQT-- -GSATTTD-N NSCESVVMSG QHQQQQNPTP
Ory_satHOX33-like             QVSRLVYENG YMRTQLHN-- -PSAATTD-- TSCESVVTSS QHQQQ-NPAV
Sol_lycREVOLUTA-like          QVSQLVCENG YMRQQLQS-- -VSAATTD-- VSCESVVT-- --------TP
Sol_lycHOX32-like             QVSQLVYENG YMKQQINT-- -VSSTTTD-- TSCESVVVSG QQQRK-NPTP
Gly_maxATHB-14-likeb          QVSQLVYDNG FMKQQIHT-- ASATTTTD-- NSCESVVVSG QRQHQ-NPKI
Pop_triXP_006378973.1         QVSQLVCENG FMQQQLQT-- --APAAAD-- ASCDSAVT-- --------TP
Ory_satACA64837.1             QVSRLVYENG YMRTQLHN-- -PSAATTD-- TSCESVVTSG QHHQQQNPAV
Vit_vin013992                 QVSQLVCENG YMRQQLQT-- -ASAATTD-- ASCESVVT-- --------TP
Ory_satOsJ_11861              QVSRLVYENG YMRQQLHN-- -PSVATTD-- TSCESVVTSG QHHQQQNPAA
Sol_tubREVOLUTA-like          QVSQLVCENG YMRQQLQS-- -VSAATTD-- VSCESVVT-- --------TP
Gly_maxATHB-14-likec          QVSQLVYDNG FMKQQIHTAS ATTTTTTD-- NSCESVVVSG QHQPQ-NPKT
The_cacXP_007024277.1         QVSQLVYENG YFRQHTQN-- -ATLATKD-- PSCESVVTSG QH----HVTP
Vit_vinATHB-15-like           QVSQLVYENG YFRQHTQN-- -TTLATKD-- TSCESVVTSG QH----HLTP
Ric_comXP_002515977.1         QVSQLVYENG YFRQHTQN-- -TTLATKD-- TSCDSVVTSG QH----HLTP
The_cacXP_007016751.1         QVSQLVCENG YMRQQLHT-- VNASAATD-- ASCDSVVT-- --------TP
Sol_tubHOX32-like             QVSQLVYENG YMKQQINT-- -VSSTTTD-- TSCESVVVSG QQQRK-NPTP
Sol_lycATHB-14-like           HVSHLVYDNG FMRQQLHT-- -VNSTTTD-- TCCESVVVSG QQQQQ-NPMP
Sol_tubATHB-14-like_X1        HVSHLVYDNG FMRQQLHT-- QVNSTTTD-- TCCESVVVSD QQQQQ-NPIP
Med_truATHB-14                QVSHLVYENG YMKQQIHTVS ASAATTTD-- NSCDSVVMSG QNQQQ-NPT-
Zea_mayNP_001142394.1         QVSRLVFDNG YMKNRLHS-- -PSVATTD-- TSCESVVTSG QHNQQQNPAV
Vit_vinREVOLUTA               QVSQLVCENG YMRQQLQT-- --VSATTD-- ASCESVVT-- --------TP
Ory_braHOX32-like             QVSRLVYENG YMRQQLHN-- -PSVATTD-- TSCESVVTSG QHHQQQNPAA
Ric_comXP_002529946.1         QVSQLVCENG YMRQQLQT-- --ASAATD-- ASCDSVVT-- --------TP
Tha_cacXP_007016749.1         QVSQLVCENG YMRQQLHT-- VNASAATD-- ASCDSVVT-- --------TP
Fra_vesREVOLUTA-like          QVSQLVCENG YMRQQLHT-- ---APATD-- ASCDSVVT-- --------TP
Zea_mayNP_001146215.1         QVSRLVFDNG YMKNRLHS-- -PSVATTD-- TSCESVVTSG QHKQQQNPAV
The_cacXP_007012152.1         QVSQLVYENS YFRQQTQN-- -ATLATTD-- TSCESVVTSG QH----HLTP
Pru_perppa001386mg            QVSQLVYENS YFRQQTQN-- -TNLATTD-- TSCESVVTSG QH----HLTP
Pop_triAAX19057.1             QVSQLVYENS YFRQQTQN-- ATNLATTD-- TSCESVVTSG QH----HLTP
Pop_triXP_002309538.2         QVSQLVYENS YFRQQTQN-- ATNLATTD-- TSCESVVTSG QH----HLTP
Fra_vesATHB-8-like            QVSHLVYENS YFRQQTQN-- -TTLATTD-- TSCESVVTSG QH----HLTP
Pop_triXP_002324794.1         QVSQLVYENS YFRQQTQN-- ATTLATTD-- TSCESVVTSG QH----RLTP
Vit_vin009744                 QVSQLVYENS FFRQQTQN-- -ATLATTD-- TSCESVVTSG QH----HLTP
Gly_maxATHB-8-like            QVSHLVYENS FFRQQTHN-- NATLATTDTN TSCESVVTSG QR----NLTP
Sol_lycATHB-15-like           QVSQLVYENS FFRQQTQT-- -AALATTD-N NSCESVVTSG QH----NLTP
Gly_maxATHB-8-likeb           QVSHLVYENS FFRQQTHN-N NATLATTDTN TSCESVVTSG QR----NLTP
Med_truXP_003603630.1         QVSHLVYENT FFRQHTQN-- -TTLATTD-- TSCESVVTSG QQ--------
```

```
                               201
AmTr_v1.0_scaffold00148.15   Q-RP-PRDAN NPAGLLSIAE ETLAEFLSKA TGTAVDWVQM PGMKPGPDSI
AmTr_v1.0_scaffold00056.60   Q-HP-PRDA- SPAGLLSIAE ETLAEFLSKA TGTAVEWIQM PGMKPGPDSI
AmTr_v1.0_scaffold00155.45   PPHP-PRDAN NPAGLLSIAE ETLAEFLSKA TGTAVDWVQM LGMKPGPDSI
Ara_thaPHABULOSA             Q-HQ-QRDAN NPAGLLSIAE EALAEFLSKA TGTAVDWVQM IGMKPGPDSI
Ara_thaATHB15                Q-NP-QRDA- SPAGLLSIAE ETLAEFLSKA TGTAVEWVQM PGMKPGPDSI
Ara-thaPHAVOLUTA             Q-HP-QRDVN NPANLLSIAE ETLAEFLCKA TGTAVDWVQM IGMKPGPDSI
Ara_thaATHB8                 Q-HQ-PRDA- SPAGLLSIAD ETLTEFISKA TGTAVEWVQM PGMKPGPDSI
Ara_thaREVOLUTA              Q-HS-LRDAN SPAGLLSIAE ETLAEFLSKA TGTAVDWVQM PGMKPGPDSV
Vit_vinHOX32-like            Q-HP-QRDAS NPAGLLAIAE ETLAEFLSKA TGTAVDWVQM IGMKPGPDSI
The_cacXP_007022870.1        Q-HP-QRDAN SPAGLLAIAE ETLAEFLSKA TGTAVDWVQM IGMKPGPDSI
Ory_satHOX32                 T-RP-QRDAN NPAGLLAIAE ETLAEFLSKA TGTAVDWVQM VGMKPGPDSI
Pru_perppa001343mg           Q-HP-QRDAN NPAGLLAIAE ETLAEFLSKA TGTAVDWVQM IGMKPGPDSI
Bra_disHOX32-like            P-RP-QRDAN NPAGLLAIAE ETLAEFLSKA TGTAVDWVQM VGMKPGPDSI
Pop_triPHABULOSA             Q-QP-QRDAN NPAGLLAIAE ETLAEFLSKA TGTAVDWVQM IGMKPGPDSI
Pop_triPHABULOSAb            Q-QP-QRDAN NPAGLLTIAE ETLAEFLSKA TGTAVDWVQM IGMKPGPDSI
Fra_vesHOX32-like            Q-HP-QRDAN NPAGLLAIAE ETLAEFLSKA TGTAVDWVQM IGMKPGPDSI
Gly_maxATHB-14-like          Q-HP-NRDAN NPAGLLAIAE ETLAEFLSKA TGTAVDWVQM IGMKPGPDSI
Gly_maxREVOLUTA-like         Q-HT-LRDAS NPAGLLSIAE ETLTEFLSKA TGTAVDWVQM PGMKPGPDSV
Gly_maxREVOLUTA-likeb        Q-HT-MRDAN NPAGLLSIAE ETLTEFLSKA TGTAVDWVQM PGMKPGPDSV
Pru_perREV_IFL1              Q-HS-LRDAN NPAGLLSIAE ETLAEFLSKA TGTAVDWVQM PGMKPGPDSV
Ory_satHOX33                 L-HP-QRDAN NPAGLLAIAE ETLAEFMSKA TGTAVEWVQM VGMKPGPDSI
Sol_tubATHB-14-like          Q-HP-ERDAN NPAGLLAIAE ETLAEFLGKA TGTAVDWVQM IGMKPGPDSI
The_cacXP_007022872.1        Q-HP-QRDAN SPAGLLAIAE ETLAEFLSKA TGTAVDWVQM IGMKPGPDSI
Ory_satHOX33-like            L-HP-QRDAN NPAGLLAIAE ETLAEFMSKA TGTAVEWVQM VGMKPGPDSI
Sol_lycREVOLUTA-like         Q-HS-LRDAN NPAGLLPIAE ETLAEFLSKA TGTAVDWVPM PGMKPGPDSV
Sol_lycHOX32-like            Q-HP-ERDAN NPAGLLAIAE ETLAEFLGKA TGTAVDWVQL IGMKPGPDSI
Gly_maxATHB-14-likeb         Q-HP-QWDAN NPAGLLAIAQ ETLAAFLSKA TGTAVNWVQM IGMKPGPDSI
Pop_triXP_006378973.1        Q-HS-LRDAN NPAGLLSLAE ETLAEFLSKA TGTAVDWVQM PGMKPGPDSV
Ory_satACA64837.1            L-HP-QRDAN NPAGLLAIAE ETLAEFMSKA TGTAVEWVQM VGMKPGPDSI
Vit_vin013992                Q-HS-LRDAN NPAGLLSIAE ETLAEFLSKA TGTAVDWVQM PGMKPGPDSV
Ory_satOsJ_11861             T-RP-QRDAN NPAGLLAIAE ETLAEFLSKA TGTAVDWVQM VGMKPGPDSI
Sol_tubREVOLUTA-like         Q-HS-LRDAN NPAGLLPIAE ETLAEFLSKA TGTAVDWVPM PGMKPGPDSV
Gly_maxATHB-14-likec         Q-HP-QWDAN NPAGLLAIAQ ETLVEFLSKA TGTAVNWVQM IGMKPGPDSI
The_cacXP_007024277.1        Q-HP-PRDA- SPAGLLSIAE ETLAEFLSKA TGTAVEWVQM PGMKPGPDSI
Vit_vinATHB-15-like          Q-HP-PRDA- SPAGLLSIAE ETLTEFLSKA TGTAVEWVQM PGMKPGPDSI
Ric_comXP_002515977.1        Q-HQ-PRDA- SPAGLLSIAE ETLTEFLSKA TGTAVEWVQM PGMKPGPDSI
The_cacXP_007016751.1        Q-HS-LRDAN NPAGLLSIAE ETLAEFLSKA TGTAVDWVQM PGMKPGPDSV
Sol_tubHOX32-like            Q-HP-ERDAN NPAGLLAIAE ETLAEFLGKA TGTAVDWVQL IGMKPGPDSI
Sol_lycATHB-14-like          QHHP-QRDAN SPAGLLAIAE ETLTEFLGKA TGTAVDWVQM IGMKPGPESI
Sol_tubATHB-14-like_X1       QHHP-QRDAN SPAGLLAIAE ETLTEFLGKA TGTAVDWVQM IGMKPGPESI
Med_truATHB-14               Q-RP-QRDAN NPAGLLAVAE ETLAEFLSKA TGTAVDWVQM IGMKPGPESI
Zea_mayNP_001142394.1        L-HPPQRDAN NPAGLLAIAE ETLAEFMSKA TGTAVNWVQM VGMKPGPDSV
Vit_vinREVOLUTA              Q-HS-LRDAN NPAGLLSIAE ETLAEFLSKA TGTAVDWVQM PGMKPGPDSV
Ory_braHOX32-like            A-RP-QRDAN NPAGLLAIAE ETLAEFLSKA TGTAVDWVQM VGMKPGPDSI
Ric_comXP_002529946.1        Q-HS-LRDAN NPAGLLSIAE ETLAEFLSKA TGTAVDWVQM PGMKPGPDSV
Tha_cacXP_007016749.1        Q-HS-LRDAN NPAGLLSIAE ETLAEFLSKA TGTAVDWVQM PGMKPGPDSV
Fra_vesREVOLUTA-like         Q-HS-LRDAN NPAGLLSIAE ETLAEFLSKA TGTAVDWVQM PGMKPGPDSV
Zea_mayNP_001146215.1        L-HPPQRDAN NPAGLLAIAE ETLAEFMSKA TGTAVNWVQM VGMKPGPDSV
The_cacXP_007012152.1        Q-HP-PRDA- SPAGLLSIAE ETLTEFLSKA TGTAVEWVQM PGMKPGPDSI
Pru_perppa001386mg           Q-HP-PRDA- SPAGLLSIAE ETLAEFLSKA TGTAVEWVQM PGMKPGPDSI
Pop_triAAX19057.1            Q-HP-PRDA- SPAGLLSIAE ETLAQFLSKA TGTAVEWVQM PGMKPGPDSI
Pop_triXP_002309538.2        Q-HP-PRDA- SPAGLLSIAE ETLAQFLSKA TGTAVEWVQM PGMKPGPDSI
Fra_vesATHB-8-like           Q-HP-PRDA- SPAGLLSIAE ETLAEFLSKA TGTAVEWVQL PGMKPGPDSI
Pop_triXP_002324794.1        Q-HP-PRDA- SPAGLLSIAE ENLAQFLSKA TGTAVEWVQM PGMKPGPDSI
Vit_vin009744                Q-HP-PRDA- SPAGLLSIAE ETLTEFLSKA TGTAVEWVQM PGMKPGPDSI
Gly_maxATHB-8-like           QQHP-PRDA- SPAGLLSIAE ETLAEFLSKA TGTAVEWVQM PGMKPGPDSI
Sol_lycATHB-15-like          Q-RP-PRDA- SPAGLLSLAE ETLTEFLSKA TGTAVEWVQM PGMKPGPDSI
Gly_maxATHB-8-likeb          QQHP-PRDA- SPAGLLSIAE ETLAEFLSKA TGTAVEWVQM PGMKPGPDSI
Med_truXP_003603630.1        --HPQPRDA- SPAGLLSIAE ETLAEFLSKA TGTAVEWVQM PGMKPGPDSI
```

	251				
AmTr_v1.0_scaffold00148.15	GIVAISHSCS	GVAA---RAC	GLVSLEPTKV	VEILKDRMSW	FRDCRNFDIL
AmTr_v1.0_scaffold00056.60	GIVAISHGCT	GVAA---RAC	GLVGLEPTKV	SEILKDRPTW	FRDCRSVEVL
AmTr_v1.0_scaffold00155.45	GIVAVSHNCS	GMAA---RVC	GLVSLEPAKV	AEILKDRPSW	FRDCRSHDVL
Ara_thaPHABULOSA	GIVAISRNCS	GIAA---RAC	GLVSLEPMKV	AEILKDRPSW	LRDCRSVDTL
Ara_thaATHB15	GIIAISHGCT	GVAA---RAC	GLVGLEPTRV	AEIVKDRPSW	FRECRAVEVM
Ara-thaPHAVOLUTA	GIVAVSRNCS	GIAA---RAC	GLVSLEPMKV	AEILKDRPSW	FRDCRCVETL
Ara_thaATHB8	GIVAISHGCT	GIAA---RAC	GLVGLDPTRV	AEILKDKPCW	LRDCRSLDIV
Ara_thaREVOLUTA	GIFAISQRCN	GVAA---RAC	GLVSLEPMKI	AEILKDRPSW	FRDCRSLEVF
Vit_vinHOX32-like	GIVAVSRNCS	GVAA---RAC	GLVSLEPTKV	AEILKDRPSW	FRDCRCLDVL
The_cacXP_007022870.1	GIVAVSRNCS	GVAA---RAC	GLVSLEPTKV	AEILKDRPSW	FRDCRCLDVL
Ory_satHOX32	GIIAVSHNCS	GVAA---RAC	GLVSLEPTKV	AEILKDRPSW	YRDCRCVDVL
Pru_perppa001343mg	GIVAVSRNCS	GVAA---RAC	GLVSLEPTKV	AEILKDRTSW	FRDCRCLDVL
Bra_disHOX32-like	GIIAVSHNCS	GVAA---RAC	GLVSLEPTKV	AEILKDRPSW	YRDCRCVDVL
Pop_triPHABULOSA	GIVAVSRNCS	GVAA---RAC	GLVSLEPTKV	AEILKDRPSW	FRDCRCLDIL
Pop_triPHABULOSAb	GIVAVSRNCS	GVAA---RAC	GLVSLEPIKV	AEILKDRPSW	FRDCRCLDIM
Fra_vesHOX32-like	GIVAVSRNTS	GVAA---RAC	GLVSLEPTKV	AEILKDRPSW	FRDCRCLDVL
Gly_maxATHB-14-like	GIVAVSRNCS	GVAA---RAC	GLVSLEPTKV	AEILKDRQSW	YRDCRCVDVL
Gly_maxREVOLUTA-like	GIFAISQSCS	GVAA---RAC	GLVSLEPTKI	AEILKDRPSW	FRDCRSLEVF
Gly_maxREVOLUTA-likeb	GIFAISQSCS	GVAA---RAC	GLVSLEPTKI	AEILKDRPSW	FRDCRSLEVF
Pru_perREV_IFL1	GIFAISQSCS	GVAA---RAC	GLVSLEPTKI	AEILKDRPSW	FRDCRSLEVF
Ory_satHOX33	GIIAVSHNCS	GVAA---RAC	GLVSLEPTKV	AEILKDRPSW	YRDCRCVDII
Sol_tubATHB-14-like	GIVAVSRNCS	GVAA---RAC	GLVSLEPTKV	AEILKDRPSW	YRDCRCLNVL
The_cacXP_007022872.1	GIVAVSRNCS	GVAA---RAC	GLVSLEPTKV	AEILKDRPSW	FRDCRCLDVL
Ory_satHOX33-like	GIIAVSHNCS	GVAA---RAC	GLVSLEPTKV	AEILKDRPSW	YRDCRCVDII
Sol_lycREVOLUTA-like	GIFAISHSCS	GVAA---RAC	GLVSLEPTKI	ADILKDRPSW	FRDCRNVEVI
Sol_lycHOX32-like	GIVAVSRNCS	GVAA---RAC	GLVSLEPTKV	AEILKDCPSW	YRDCRCLNVL
Gly_maxATHB-14-likeb	GIVAVSRNCS	GVAA---RAC	GLVSLEPTKV	AEILKDRPSW	YRDCRCLNVL
Pop_triXP_006378973.1	GIFAISQRCS	GVAA---RAC	GLVSLEPTKL	AEILKDRQSW	FRDCRNLEVF
Ory_satACA64837.1	GIITVSHNCS	GVAA---RAC	GLVSLEPTKV	AEILKDRPSW	YRDCRCVDII
Vit_vin013992	GIFAISHSCS	GSGSSSMRSC	KFRTLK----	-EILKDRPSW	FRDCRSLEVF
Ory_satOsJ_11861	GIIAVSHNCS	GVAA---RAC	GLVSLEPTKV	AEILKDRPSW	YRDCRCVDVL
Sol_tubREVOLUTA-like	GIFAISHSCS	GVAA---RAC	GLVSLEPTKI	AEILKDRPSW	FRDCRNVEVI
Gly_maxATHB-14-likec	GIVAVSRNCS	GVAA---RAC	GLVSLEPTKV	AEILKDRPSW	YRDCRCLNVL
The_cacXP_007024277.1	GIVAISHGCT	GVAA---RAC	GLVGLEPTRV	AELLKDRPSW	FRDCRAVDVL
Vit_vinATHB-15-like	GIVAISHGCT	GVAA---RAC	GLVGLEPTRV	AEILKDRPSW	FRDCRAVDVL
Ric_comXP_002515977.1	GIIAISHGCT	GVAA---RAC	GLVGLEPTRV	AEILKDRPSW	FRDCRAVDVL
The_cacXP_007016751.1	GIFAISQSCS	GVAA---RAC	GLVSLEPTKI	AEILKDRPSW	FRDCRNLEVF
Sol_tubHOX32-like	GIVAVSRNCS	GVAA---RAC	GLVSLEPTKV	AEILKDCPSW	YRDCRCLNVL
Sol_lycATHB-14-like	GIVAVSRNCN	GVAA---RAC	GLVSLEPMKV	AEILKDRPSW	YRDCRCLDIL
Sol_tubATHB-14-like_X1	GIVAVSRNCN	GVAA---RAC	GLVSLEPMKV	AEILKDRPSW	YRDCRCLDIL
Med_truATHB-14	GIVAVSRNSS	GIAA---RAC	GLVSLEPTKV	AEILKDRLSW	YRDCRCVDVL
Zea_mayNP_001142394.1	GIIAVSHNCS	GVAA---RAC	GLVSLEPTKV	AEILKDRASW	YRDCRHVDVL
Vit_vinREVOLUTA	GIFAISHSCS	GVAA---RAC	GLVSLEPSKI	AEILKDRPSW	FRDCRSLEVF
Ory_braHOX32-like	GIIAVSHNCS	GVAA---RAC	GLVSLEPTKV	AEILKDRPSW	YRDCRCVDVL
Ric_comXP_002529946.1	GIFAISQSCS	GVAA---RAC	GLVSLEPTKI	AEILKDRPSW	FRDCRSLEVF
Tha_cacXP_007016749.1	GIFAISQSCS	GVAA---RAC	GLVSLEPTKI	AEILKDRPSW	FRDCRNLEVF
Fra_vesREVOLUTA-like	GIFAISQSCS	GVAA---RAC	GLVSLEPTKI	AEILKDRPSW	FRDCRSLEVF
Zea_mayNP_001146215.1	GIIAVSHNCS	GVAA---RAC	GLVSLEPTKV	AEILKDRASW	YRDCRRVDIL
The_cacXP_007012152.1	GIVAISHGCT	GVAA---RAC	GLVGLDPTRV	AEILKDRPSW	FRDCRAVDVM
Pru_perppa001386mg	GIVAISHGCT	GVAA---RAC	GLVGLEPTRV	AEILKDRPSW	FRNCRSVDVL
Pop_triAAX19057.1	GIVAISHGCT	GVAA---RAC	GLVGLEPTRV	AEILKDRPSW	FRDCRAVDVV
Pop_triXP_002309538.2	GIVAISHGCT	GVAA---RAC	GLVGLEPTRV	AEILKDRPSW	FRDCRAVDVV
Fra_vesATHB-8-like	GIVAISHGCT	GVAA---RAC	GLVGLEPTRV	SEILKDRPSW	FRNCRSVDVL
Pop_triXP_002324794.1	GIVAISHGCT	GVAA---RAC	GLVGLEPTRV	AEILKDRPSW	FRDCRAVDVI
Vit_vin009744	GIVAISHGCT	GVAA---RAC	GLVSLEPTRV	AEILKDWPSW	YRECRNVDVL
Gly_maxATHB-8-like	GIVAISHGCP	GVAA---RAC	GLVGLEPARV	AEILKDRLSW	FRDCRTVDVL
Sol_lycATHB-15-like	GIIAISHGCS	GVAS---RAC	GLVGLEPTRV	AEILKDRPSW	FRDCRAVDVL
Gly_maxATHB-8-likeb	GIVAISHGCP	GVAA---RAC	GLVGLEPTRV	AEILKDRLSW	FRDCRTVDVL
Med_truXP_003603630.1	GIVAISHGSP	GVAA---RAC	GLVGLEPARV	AEILKDRLSW	YRDCRTVDVL

	301				
AmTr_v1.0_scaffold00148.15	TVLPLGSGGT	IELIYMQT--	YAPTTLA---	----PARDFW	TLRYTCGLED
AmTr_v1.0_scaffold00056.60	TALSTGNGGT	IELLYMQM--	YAPTTLA---	----SARDFW	LLRYTSVLED
AmTr_v1.0_scaffold00155.45	TVFPAGNGGT	IELIYMQT--	YAPTTLA---	----AARDFW	TLRYTTGLDD
Ara_thaPHABULOSA	SVIPAGNGGT	IELIYTQM--	YAPTTLA---	----AARDFW	TLRYSTCLED
Ara_thaATHB15	NVLPTANGGT	VELLYMQL--	YAPTTLA---	----PPRDFW	LLRYTSVLED
Ara-thaPHAVOLUTA	NVIPTGNGGT	IELVNTQI--	YAPTTLA---	----AARDFW	TLRYSTSLED
Ara_thaATHB8	NVLSTANGGT	LELIYMQL--	YAPTTLA---	----PARDFW	MLRYTSVMED
Ara_thaREVOLUTA	TMFPAGNGGT	IELVYMQT--	YAPTTLA---	----PARDFW	TLRYTTSLDN
Vit_vinHOX32-like	SVIPTGNGGT	IELIYMQT--	YAPTTLA---	----SARDFW	TLRYTTSLED
The_cacXP_007022870.1	SVIPTGNGGT	IELIYMQT--	YAPTTLA---	----AARDFW	TLRYTTSLED
Ory_satHOX32	HVIPTGNGGT	IELIYMQT--	YAPTTLA---	----APRDFW	ILRYTSGLED
Pru_perppa001343mg	SLIPAGNGGT	IELTYMQT--	YAPTTLA---	----AARDFW	TLRYTTSLED
Bra_disHOX32-like	QIIPTGNGGT	IELIYMQT--	YAPTTLA---	----APRDFW	ILRYTSGLED
Pop_triPHABULOSA	SVIPTGSGGT	IELIYMQT--	YAPTTLA---	----AARDFW	TLRYTTTLED
Pop_triPHABULOSAb	SVIPTGSGGT	IELIYMQT--	YAPTTLA---	----AARDFW	TLRYTTTLED
Fra_vesHOX32-like	SIIPAGNGGT	IELLYMQI--	YAPTTLA---	----AARDFW	TLRYTTTLED
Gly_maxATHB-14-like	SIVPTGNGGT	IELMYMQT--	YAPTTLA---	----AARDFW	TLRYTTSLED
Gly_maxREVOLUTA-like	TMFPAGNGGT	IELVYTQT--	YAPTTLA---	----PARDFW	TLRYTTSLEN
Gly_maxREVOLUTA-likeb	TMFPAGNGGT	IELVYTQT--	YAPTTLA---	----PARDFW	TLRYTTSLEN
Pru_perREV_IFL1	TMFPAGNGGT	IELIYTQT--	YAPSTLA---	----PARDFW	TLRYTTSLDN
Ory_satHOX33	HVIPTGNGGT	IELIYMQT--	YAPTTLA---	----APRDFW	TLRYTSGLED
Sol_tubATHB-14-like	SVIPTGNGGT	IELIYLQT--	YAPTTLA---	----TARDFW	TLRYTTSLED
The_cacXP_007022872.1	SVIPTGNGGT	IELIYMQT--	YAPTTLA---	----AARDFW	TLRYTTSLED
Ory_satHOX33-like	HVIPTGNGGT	IELIYMQT--	YAPTTLA---	----APRDFW	TLRYTSGLED
Sol_lycREVOLUTA-like	TMFPAGNGGT	VELLYTQI--	YAPTTLA---	----PARDFW	TLRYTTTLDN
Sol_lycHOX32-like	SVIPTGNGGT	IELIYLQT--	YAPTTLA---	----TARDFW	TLRYTTSLED
Gly_maxATHB-14-likeb	SVIPTGNGGT	IELMYMQT--	YAPTTLA---	----AARDFW	TLRYTTSLED
Pop_triXP_006378973.1	TVFPAGNGGT	IELLYSQI--	YAPTTLA---	----PARDFW	TLRYTINLEN
Ory_satACA64837.1	HVIPTGNGGT	IELIYMQT--	HAPTTLA---	----APRDFW	TLRYTSGLED
Vit_vin013992	TMFPAGNGGT	VELLYTQI--	YAPTTLA---	----PARDFW	TLRYTTSLDN
Ory_satOsJ_11861	HVIPTGNGGT	IELIYMQT--	YAPTTLA---	----APRDFW	ILRYTSGLED
Sol_tubREVOLUTA-like	TIFPAGNGGT	VELLYTQI--	YAPTTLA---	----PARDLW	TLRYTTTLDN
Gly_maxATHB-14-likec	SVVSAGNGGT	IELMYMQT--	YAPTTLA---	----AARDFW	TLRYSTSLED
The_cacXP_007024277.1	NVLPTANGGT	IELLYMQL--	YAPTTLA---	----PARDFW	LLRYTSVLED
Vit_vinATHB-15-like	NVLPTANGGT	IELLYMQL--	YAPTTLA---	----PARDFW	LLRYTSVMED
Ric_comXP_002515977.1	NVLPTANGGT	IELLYMQL--	YAPTTLA---	----PARDFW	LLRYTSVLED
The_cacXP_007016751.1	TMFPAGNGGT	IELVYTQT--	YAPTTLA---	----PARDFW	TLRYTTTLEN
Sol_tubHOX32-like	SVIPTGNGGT	IELIYLQT--	YAPTTLA---	----TARDFW	TLRYTTSLED
Sol_lycATHB-14-like	NVIPTGNGGT	IELIYLQT--	YAPTTLA---	----PARDFW	TLRYTTSLED
Sol_tubATHB-14-like_X1	NVIPTGNGGT	IELIYLQT--	YAPTTLA---	----PARDFW	TLRYTTSLED
Med_truATHB-14	SIVPTGGGGT	IELMYMQVLF	YSTKELTMPF	LFLLTSRDFW	TLRYTTSLED
Zea_mayNP_001142394.1	HVIPTGNGGT	IELIYMQT--	YALTTLA---	----EPRDFW	TLRYTSGLDD
Vit_vinREVOLUTA	TMFPAGNGGT	VELLYTQI--	YAPTTLA---	----PARDFW	TLRYTTSLDN
Ory_braHOX32-like	HVIPTGNGGT	IELIYMQT--	YAPTTLA---	----APRDFW	TLRYTSGLED
Ric_comXP_002529946.1	TMFPAGNGGT	IELVYSQV--	YAPTTLA---	----PARDFW	TLRYTSSLDN
Tha_cacXP_007016749.1	TMFPAGNGGT	IELVYTQT--	YAPTTLA---	----PARDFW	TLRYTTTLEN
Fra_vesREVOLUTA-like	TMFPAGNGGT	IELIYTQT--	YAPTTLA---	----PARDFW	TLRYTTSLDN
Zea_mayNP_001146215.1	HVIPTGNGGT	IELIYMQT--	YALTTLA---	----EPRDFW	TLRYTSGLDD
The_cacXP_007012152.1	NVLSTGNGGT	IELLYMQL--	YAPTTLA---	----PARDFW	LLRYTSVLED
Pru_perppa001386mg	NVLSTGNGGT	IELLYMQL--	YAPTTLA---	----PARDFW	LLRYTSVLED
Pop_triAAX19057.1	NALSTGSGGT	IELLYMQL--	YAPTTLA---	----PARDFW	LLRYTSVLED
Pop_triXP_002309538.2	NALSTGSGGT	IELLYMQL--	YAPTTLA---	----PARDFW	LLRYTSVLED
Fra_vesATHB-8-like	NVLSTGNGGT	IELLYMQL--	YAPTTLA---	----PARDFW	LLRYTSVLED
Pop_triXP_002324794.1	NAMSTANGGT	IELLYMQL--	YAPTTLA---	----PARDFL	LLRYTSVLED
Vit_vin009744	NVLSTGNGGT	IELLYMQL--	YAPTTLA---	----PARDFW	LLRYTSVLED
Gly_maxATHB-8-like	NVMSTGNGGT	IELLYMQL--	YAPTTLA---	----PGRDFW	LLRYTSLLED
Sol_lycATHB-15-like	NVMSTGNGGT	IELLYMQL--	YAPTTLA---	----PARDFW	LMRYTSVMED
Gly_maxATHB-8-likeb	NVMSTGNGGT	IELLYMQL--	YAPTTLA---	----PGRDFW	LLRYTSLLED
Med_truXP_003603630.1	NVMSTGNGGT	IELLYMQL--	YAPTTLA---	----PGRDFW	LLRYTSLLED

```
                             351
AmTr_v1.0_scaffold00148.15   GSLVVCERSL TSSNGSPHGQ PVPNFVRAEM LPSGYLIRPC EGGGSIIHIV
AmTr_v1.0_scaffold00056.60   GSLVVCERSL SNTQGGPSMP PVQQFVRAEM HPSGYLIRPC EGGGSIIHLV
AmTr_v1.0_scaffold00155.45   GSLVVCERSL TSSTGGPSGP TSLSFVRAEM MPSGYLIRPC EGGGSIIHIV
Ara_thaPHABULOSA             GSYVVCERSL TSATGGPTGP PSSNFVRAEM KPSGFLIRPC DGGGSILHIV
Ara_thaATHB15                GSLVVCERSL KSTQNGPSMP LVQNFVRAEM LSSGYLIRPC DGGGSIIHIV
Ara-thaPHAVOLUTA             GSYVVCERSL TSATGGPNGP LSSSFVRAKM LSSGFLIRPC DGGGSIIHIV
Ara_thaATHB8                 GSLVICERSL NNTQNGPSMP PSPHFVRAEI LPSGYLIRPC EGGGSILHIV
Ara_thaREVOLUTA              GSFVVCERSL SGSGAGPNAA SASQFVRAEM LSSGYLIRPC DGGGSIIHIV
Vit_vinHOX32-like            GSLVICERSL TSSTGGPTGP PASSYIRAEM LPSGYLIRPC EGGGSIIHIV
The_cacXP_007022870.1        GSLVICERSL TSSTGGPTGP PTSSFVRAEM LPSGFLIRPC EGGGSIIHIV
Ory_satHOX32                 GSLVICERSL TQSTGGPSGP NTPNFVRAEV LPSGYLIRPC EGGGSMIHIV
Pru_perppa001343mg           GSLVVCERSL TSSTGGPTGP PSASFVRAEM LPSGYLIRPC EGGGSIINIV
Bra_disHOX32-like            GSLVICERSL TQATGGPSGP NTPNFVRAEV LPSGYLIRPC EGGGSMIHIV
Pop_triPHABULOSA             GSLVICERSL TSSTGGPTGP PPSSFIRAEM LPSGYLIRPC EGSGSIIHIV
Pop_triPHABULOSAb            GSLVICERSL TSSTGGPTGP PSSSFVRADM LPSGYLIRPC EGGGSIIHIV
Fra_vesHOX32-like            GSLVVCERSL TSSTGGPTGT PSPSFVRAEM LPSGFLIRPC EGGGSIIHIV
Gly_maxATHB-14-like          GSLVICERSL TSSTGGPAGP PSTTFVRAEM LPSGFLIRPC EGGGSIIHIV
Gly_maxREVOLUTA-like         GSLVVCERSL SGSGTGPNPA AAAQFVRAET LPSGYLIRPC EGGGSIIHIV
Gly_maxREVOLUTA-likeb        GSLVVCERSL SGSGTGPNPA AAAQFVRAET LPSGYLIRPC EGGGSIIHIV
Pru_perREV_IFL1              GSFVVCERSL SGSGAGPNAA SAAQFVRAEM LPSGYLIRPC EGGGSIIHIV
Ory_satHOX33                 GSLVICERSL TQSTGGPSGP NTPNFIRAEV LPSGYLIRPC EGGGSMIYIV
Sol_tubATHB-14-like          GSLVICERSL TTATGGPTGP PATSFVRAEM LPSGYLIRPC EGGGSMIHIV
The_cacXP_007022872.1        GSLVICERSL TSSTGGPTGP PTSSFVRAEM LPSGFLIRPC EGGGSIIHIV
Ory_satHOX33-like            GSLVICERSL TQSTGGPSGP NTPNFIRAEV LPSGYLIRPC EGGGSMIYIV
Sol_lycREVOLUTA-like         GSLVVCERSL SGNGPGPNPT AASQFVRAQM LPSGYLIRPC DGGGSIIHIV
Sol_lycHOX32-like            GSLVICERSL TTATGGPTGP PATSFVRAEM LPSGYLIRPC EGGGSMIHIV
Gly_maxATHB-14-likeb         GSLVICERSL TSSTGGPTGP AASNFVRAEM LPSGYLIRSC EGGGSIVHIV
Pop_triXP_006378973.1        GSLVVCERSL SGSGAGPNAA AAAQFVRAEM LPSGYLIRPC EGGGSIIHIV
Ory_satACA64837.1            GSLVICERSL TQSTGGPSGP NTLNFIRAEV LPSGYLIRPC EGGGSMIYIV
Vit_vin013992                GSLVVCERSL SGSGAGPNTA AAAQFVRAEM LPSGYLIRPC EGGGSIIHIV
Ory_satOsJ_11861             GSLVICERSL TQSTGGPSGP NTPNFVRAEV LPSGYLIRPC EGGGSMIHIV
Sol_tubREVOLUTA-like         GSLVVCERSL SGNGPGPNPA AASQFVRAQM LPSGYLIRPC DGGGSIIHIV
Gly_maxATHB-14-likec         GSLVICERSL TSSTGGPTGP AASNFIRAEM LPSGYLIRSC EGGGSIIHIV
The_cacXP_007024277.1        GSLVVCERSL KNTQNGPSMP AVQHFVRAEM LPSGYLIRPC EGGGSIIHIV
Vit_vinATHB-15-like          GSLVVCERSL KNTQNGPSMP PVQHFVRAEM LPSGYLIRPC EGGGSIIHIV
Ric_comXP_002515977.1        GSLVICERSL KNTQNGPSMP PVQHFVRAEM LPSGYLIRPC EGGGSIIHIV
The_cacXP_007016751.1        GSLVVCERSL SGSGAGPSAA AAAQFVRAEM LPSGYLIRPC EGGGSIIHIV
Sol_tubHOX32-like            GSLVICERSL TTATGGPTGP PATSFVRAEM LPSGYLIRPC EGGGSMIHIV
Sol_lycATHB-14-like          GSLVICERSL TSNTGGPVGP PATSSVRAKM LPSGFLIRPC EGGGSIIHIV
Sol_tubATHB-14-like_X1       GSLVICERSL TSNTGGPVGP PATSSVRAKM LPSGFLIRPC EGGGSIIHIV
Med_truATHB-14               GSLVICERSL NASTGGPTGP SPSNFVRAEM LPSGFLIRPC EGGGSIIHIV
Zea_mayNP_001142394.1        GSLVICERSL TQSTGGPSGP NTPNFIRAEV LPSGYLIRPC DGGGSMIYIV
Vit_vinREVOLUTA              GSLVVCERSL SGSGAGPNTA AAAQFVRAEM LPSGYLIRPC EGGGSIIHIV
Ory_braHOX32-like            GSLVICERSL TQSTGGPSGP NTPNFVRADV LPSGYLIRPC EGGGSMIHIV
Ric_comXP_002529946.1        GSLVVCERSL SGSGAGPNAA AAAQFVRAEM LPSGYLIRPC DGGGSIIHIV
Tha_cacXP_007016749.1        GSLVVCERSL SGSGAGPSAA AAAQFVRAEM LPSGYLIRPC EGGGSIIHIV
Fra_vesREVOLUTA-like         GSFVVCERSL SGSGAGPSGA SAAQFVRAEM LPSGYLIRPC EGGGSIIHIV
Zea_mayNP_001146215.1        GSLVICERSL THSTGGPSGP KTPDFIRAEV LPSGYLIRPC DGGGSMIYIV
The_cacXP_007012152.1        GSLVVCERSL NNTQNGPSIP PAANFVRAEM LPSGYLIRPC EGGGSIIHIV
Pru_perppa001386mg           GSLVVCERSL NNTQNGPSMP PVQNFVRAEM LPSGYLIRPC EGGGSIIHIV
Pop_triAAX19057.1            GSLVVCERSL NNTQNGPSMP PTQHFVRAEM LPSGYLIRPC EGGGSIIHVV
Pop_triXP_002309538.2        GSLVVCERSL NNTQNGPSMP PTQHFVRAEM LPSGYLIRPC EGGGSIIHVV
Fra_vesATHB-8-like           GSLVVCERSL NNTQNGPSMP PVQNFVRAEM LPSGYLIRPC EGGGSIIHIV
Pop_triXP_002324794.1        GSLVVCERSL NNTQNGPSMP PTQHFVRAEM LPSGYLIRPC EGGGSIIHIV
Vit_vin009744                GSLVVCERSL NNTQNGPSMP PVQYFVRAEK LPSGYLIRPC EGGGSIIHIV
Gly_maxATHB-8-like           GSLVVCERSL NNTQNGPAMP PVQHFVRADM LASGYLIRPC EGGGSIIHIV
Sol_lycATHB-15-like          GSLVICERSL NNTQNGPSMP PVQSFVRADI LPSGYLIRPC EGGGSIIHIV
Gly_maxATHB-8-likeb          GSFVVCERSL NNTQNGPAMP PVQHFVRADM LPSGYLIRPC EGGGSIIHIV
Med_truXP_003603630.1        GSLVVCERSL NNTQNGPSMP PVPHFVRAEV LPSGYLIRPC EGGGSIIHIV
```

```
                            401
AmTr_v1.0_scaffold00148.15  DHLNLEAWSV PEVLRPLYES SKVLAQKMTI AALRHIRQIA QEMSGEV--V
AmTr_v1.0_scaffold00056.60  DHMDLEPWSV PEVLRPLYES STVLAQKMTM AALRHLRQIA QEVSQNT--V
AmTr_v1.0_scaffold00155.45  DHVDLEAWNV PEVLRPLYES SKILAQKMTL AALRHIRQIA HEASGEV--N
Ara_thaPHABULOSA            DHVDLDAWSV PEVMRPLYES SKILAQKMTV AALRHVRQIA QETSGEV--Q
Ara_thaATHB15               DHMDLEACSV PEVLRPLYES PKVLAQKTTM AALRQLKQIA QEVTQTNSSV
Ara-thaPHAVOLUTA            DHVDLDVSSV PEVLRPLYES SKILAQKMTV AALRHVRQIA QETSGEV--Q
Ara_thaATHB8                DHFDLEPWSV PEVLRSLYES STLLAQRTTM AALRYLRQIS QEISQPN--V
Ara_thaREVOLUTA             DHLNLEAWSV PDVLRPLYES SKVVAQKMTI SALRYIRQLA QESNGEV--V
Vit_vinHOX32-like           DHVDLDAWSV PEVLRPLYES SKILAQKTTV AALRHIRQIA QETSGEI--Q
The_cacXP_007022870.1       DHVDLDVWSV PEVLRPLYES SKILAQKMTI AALRHIRQIA QETSGEI--Q
Ory_satHOX32                DHVDLDAWSV PEVLRPLYES PKILAQKMTI AALRHIRQIA HESSGEM--P
Pru_perppa001343mg          DHVDLDAWSV PEVLRSLYES SKILAQKMTI SALRHIRQIA QETSGEI--Q
Bra_disHOX32-like           DHVDLDAWSV PEVLRPLYES PKILAQKMTI AALRHIRQIA HESSGEM--P
Pop_triPHABULOSA            DHVDLDVWSV PEVLRPLYES SKILAQKMTM AALRHIRQIA QETSGEI--Q
Pop_triPHABULOSAb           DHVDLDVWSV PEVLRPLYES SKILAQKMTM AALRYIRQIA QETSGEI--Q
Fra_vesHOX32-like           DHVDLDAWSV PEVLRSLYES SKIIAQKMTV AALRHIRQIA QEATGEI--Q
Gly_maxATHB-14-like         DHIDLDVWSV PEVLRPLYES SKILAQKLTI AALQHIRQIA LESSGEI--Q
Gly_maxREVOLUTA-like        DHLNLEAWSV PEVLRPLYES SKVVAQKMTI AALRYIRQIA QETSGEV--V
Gly_maxREVOLUTA-likeb       DHLNLEAWSV PEVLRPLYES SKVVAQKMTI AALRYIRQIA QETSGEV--V
Pru_perREV_IFL1             DHLNLEAWSV PEVLRPLYES SKVVAQRMTI AALRYIRQIA QETSGEV--V
Ory_satHOX33                DHVDLDAWSV PEVLRPLYES PKILAQKMTI AALRHIRQIA HESSGEI--P
Sol_tubATHB-14-like         DHIDLDAWSV PEVLRPLYES SKILAQKMTM AALRHIRQIA QETSGEI--Q
The_cacXP_007022872.1       DHVDLDVWSV PEVLRPLYES SKILAQKMTI AALRHIRQIA QETSGEI--Q
Ory_satHOX33-like           DHVDLDAWSV PEVLRPLYES PKILAQKMTI AALRHIRQIA HESSGEI--P
Sol_lycREVOLUTA-like        DHLNLEAWSA PEILRPLYES SKVVAQKMTI AALRYARQLA QETSGEV--V
Sol_lycHOX32-like           DHIDLDAWSV PEVLRPLYES SKILAQKTTM AALRHIRQIA QETSGEI--Q
Gly_maxATHB-14-likeb        DHVDLDVWSV PEVLRPLYES PKFLAQKLTT AALRNVRQIA QESSGEV--Q
Pop_triXP_006378973.1       DHLNLQAWSV PEVLRPLYES SKAVAQKVTI TALRHVRQIA HETSGEV--V
Ory_satACA64837.1           DHVDLDAWSV PEVLRPLYES PKILAQKMTI AALRHIRQIA HESSGEI--P
Vit_vin013992               DHLNLEAWSV PEVLRPLYES SRVVAQKMTI AALRYIRQIA QETSGEV--V
Ory_satOsJ_11861            DHVDLDAWSV PEVLRPLYES PKILAQKMTI AALRHIRQIA HESSGEM--P
Sol_tubREVOLUTA-like        DHLNLEAWSA PEILRPLYES SKVVAQKMTI AALRYARQLA QETSGEV--V
Gly_maxATHB-14-likec        DHVDLDVWSV PEVLRPLYES PKFLAQKLTT AALRHARQIA QESSGDV--H
The_cacXP_007024277.1       DHMDLEPWRV PEVLRPLYES STVLAQKTTM AALRQLRQIA QEVSQSN--V
Vit_vinATHB-15-like         DHMDLEPWSV PEVLRPLYES STVLAQKTTM AALRQLRQIA QEVSQSN--V
Ric_comXP_002515977.1       DHMDLEPWSV PEVLRPLYES STVLAQKTTM AALRQLRQIA QEASQSN--V
The_cacXP_007016751.1       DHMNLEAWSV PEVLRPLYES SKVIAQKMTI AALRYIRQIA QETSGEV--V
Sol_tubHOX32-like           DHIDLDAWSV PEVLRPLYES SKILAQKTTM AALRHIRQIA QETSGEI--Q
Sol_lycATHB-14-like         DHIDLDACSV PEVLRPLYES SKILAQKLTM AAFRYIRQIA QETSGEI--Q
Sol_tubATHB-14-like_X1      DHIDLDACSV PEVLRPLYES SKILAQKMTM AAFRYIRQIA QETSGEI--Q
Med_truATHB-14              DHVDLDVWSV PEVLRPLYES SKILAQKLTI AALQHIKQIA QESSGEI--Q
Zea_mayNP_001142394.1       DHVNLNACSV PEVLRPLYES PKILAQKMTA AALRHIRQIA HESSGEM--P
Vit_vinREVOLUTA             DHLNLEAWSV PEVLRPLYES SRVVAQKMTI AALRYIRQIA QETSGEV--V
Ory_braHOX32-like           DHVDLDAWSV PEVLRPLYES PKILAQKMTI AALRHIRQIA HESSGEM--P
Ric_comXP_002529946.1       DHLNLEAWSV PEVLRPLYES SKVVAQKMTI AALRFIRQIA QETSGEV--V
Tha_cacXP_007016749.1       DHMNLEAWSV PEVLRPLYES SKVIAQKMTI AALRYIRQIA QETSGEV--V
Fra_vesREVOLUTA-like        DHLNLEAWSV PEVLRPLYES SKVVAQKRTI AALRYIRQIA QETSGEV--V
Zea_mayNP_001146215.1       DHVDLNACSV PEVLRPLYES PKILAQKMTA AALRHIRQIA HESSGET--P
The_cacXP_007012152.1       DHMDLEPWSV PEVLRPLYES STLLAQKTTM AALRHLRQIS QEISQPN--V
Pru_perppa001386mg          DHMDLEPWSV PEVLRPLYES STLLAQKTTM AALRNLRQIS QEVSQPN--A
Pop_triAAX19057.1           DHMDLEPWSV PEVLRPLYES STLLAQKTTM AALRHLRQVS QEVSQPN--V
Pop_triXP_002309538.2       DHMDLEPWSV PEVLRPLYES STLLAQKTTM AALRHLRQVS QEVSQPN--V
Fra_vesATHB-8-like          DHMDLEPWNV PEVLRPLYES STLLAQKTTM AALRNLRQIS QEVSQPN--T
Pop_triXP_002324794.1       DHMDLEPWSV PEVLRPLYES STLLAQKTTM AALRHLRQIS QEISQPN--V
Vit_vin009744               DHMDLEPWSV PEVLRPLYES STLLAQKTTM AALRQLRQIS QEVSQPT--N
Gly_maxATHB-8-like          DHMVLEPWSV PEVLRPLYES SMLLAQRTTM AALRHLRQIS QEVSQPS--V
Sol_lycATHB-15-like         DHMDLEPWSV PEVLRPLYES STLLSQRTTM AALRHLRQIS QEISHPT--V
Gly_maxATHB-8-likeb         DHMVLEPWSV PEVLRPLYES SMLLAQRTTM AALRHLRQIS QEVSQPS--V
Med_truXP_003603630.1       DHMLLEPWSV PEVLRPLYES SMLLAQRTTM AALRHLRQIS QEVSQPS--V
```

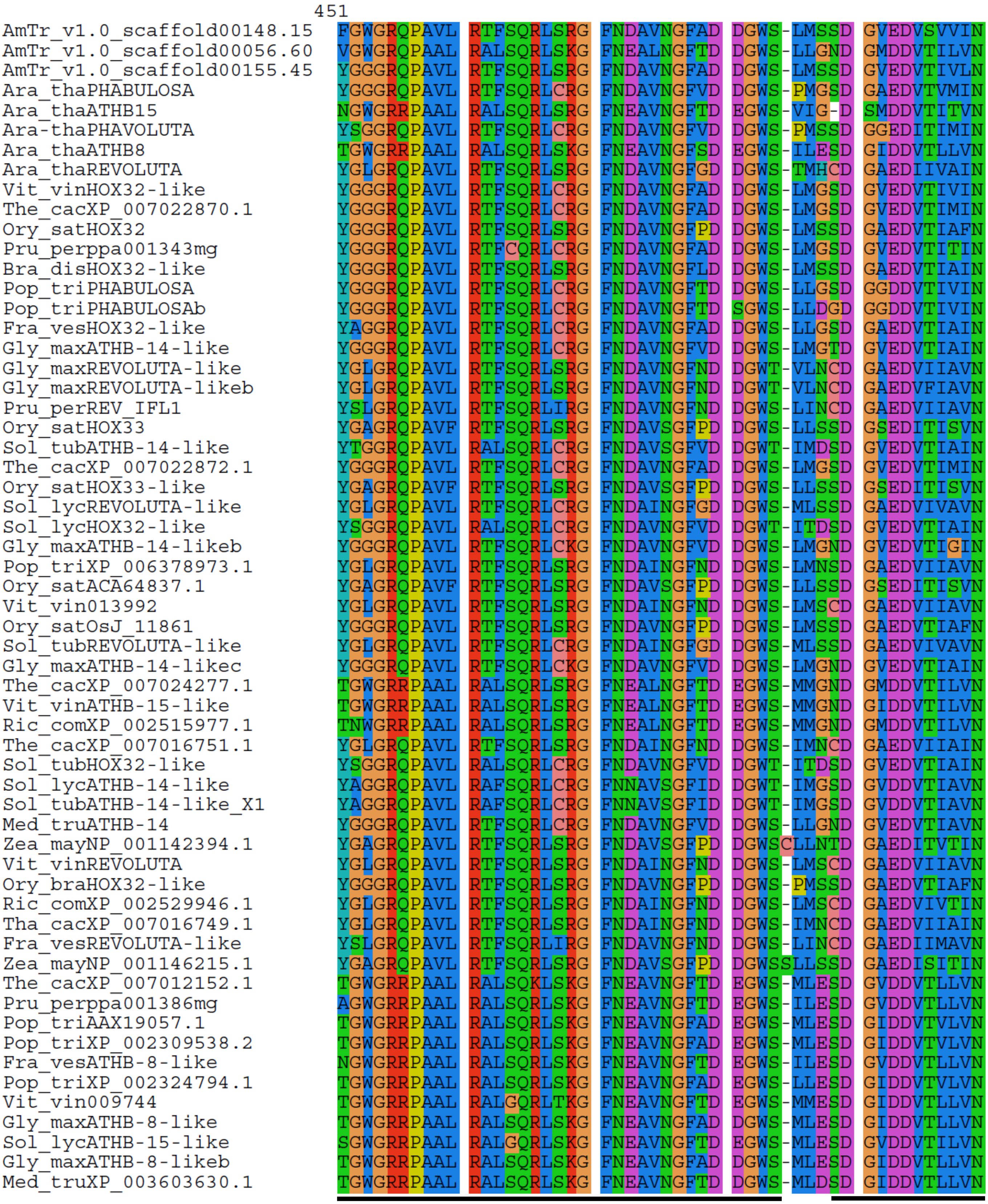
451
AmTr_v1.0_scaffold00148.15 FGWGRQPAVL RTFSQRLSRG FNDAVNGFAD DGWS-LMSSD GVEDVSVVIN
AmTr_v1.0_scaffold00056.60 VGWGRQPAAL RALSQRLSKG FNEALNGFTD DGWS-LLGND GMDDVTILVN
AmTr_v1.0_scaffold00155.45 YGGGRQPAVL RTFSQRLSRG FNDAVNGFAD DGWS-LMSSD GVEDVTIVLN
Ara_thaPHABULOSA YGGGRQPAVL RTFSQRLCRG FNDAVNGFVD DGWS-PMGSD GAEDVTVMIN
Ara_thaATHB15 NGWGRRPAAL RALSQRLSRG FNEAVNGFTD EGWS-VIG-D SMDDVTITVN
Ara-thaPHAVOLUTA YSGGRQPAVL RTFSQRLCRG FNDAVNGFVD DGWS-PMSSD GGEDITIMIN
Ara_thaATHB8 TGWGRRPAAL RALSQRLSKG FNEAVNGFSD EGWS-ILESD GIDDVTLLVN
Ara_thaREVOLUTA YGLGRQPAVL RTFSQRLSRG FNDAVNGFGD DGWS-TMHCD GAEDIIVAIN
Vit_vinHOX32-like YGGGRQPAVL RTFSQRLCRG FNDAVNGFAD DGWS-LMGSD GVEDVTIVIN
The_cacXP_007022870.1 YGGGRQPAVL RTFSQRLCRG FNDAVNGFAD DGWS-LMGSD GVEDVTIMIN
Ory_satHOX32 YGGGRQPAVL RTFSQRLSRG FNDAVNGFPD DGWS-LMSSD GAEDVTIAFN
Pru_perppa001343mg YGGGRQPAVL RTFCQRLCRG FNDAVNGFAD DGWS-LMGSD GVEDVTITIN
Bra_disHOX32-like YGGGRQPAVL RTFSQRLSRG FNDAVNGFLD DGWS-LMSSD GAEDVTIAIN
Pop_triPHABULOSA YGGGRQPAVL RTFSQRLCRG FNDAVNGFTD DGWS-LLGSD GGDDVTIVIN
Pop_triPHABULOSAb YGGGRQPAVL RTFSQRLCRG FNDAVNGFTD SGWS-LLDGD GGDDVTIVIN
Fra_vesHOX32-like YAGGRQPAVL RTFSQRLCRG FNDAVNGFAD DGWS-LLGSD GAEDVTIAIN
Gly_maxATHB-14-like YGGGRQPAVL RTFSQRLCRG FNDAVNGFVD DGWS-LMGTD GVEDVTIAIN
Gly_maxREVOLUTA-like YGLGRQPAVL RTFSQRLSRG FNDAVNGFND DGWT-VLNCD GAEDVIIAVN
Gly_maxREVOLUTA-likeb YGLGRQPAVL RTFSQRLSRG FNDAVNGFND DGWT-VLNCD GAEDVFIAVN
Pru_perREV_IFL1 YSLGRQPAVL RTFSQRLIRG FNDAVNGFND DGWS-LINCD GAEDVIIAVN
Ory_satHOX33 YGAGRQPAVF RTFSQRLSRG FNDAVSGFPD DGWS-LLSSD GSEDITISVN
Sol_tubATHB-14-like YTGGRQPAVL RALSQRLCRG FNDAVSGFVD DGWT-IMDSD GVEDVTIAIN
The_cacXP_007022872.1 YGGGRQPAVL RTFSQRLCRG FNDAVNGFAD DGWS-LMGSD GVEDVTIMIN
Ory_satHOX33-like YGAGRQPAVF RTFSQRLSRG FNDAVSGFPD DGWS-LLSSD GSEDITISVN
Sol_lycREVOLUTA-like YGLGRQPAVL RTFSQRLCRG FNDAINGFGD DGWS-MLSSD GAEDVIVAVN
Sol_lycHOX32-like YSGGRQPAVL RALSQRLCRG FNDAVNGFVD DGWT-ITDSD GVEDVTIAIN
Gly_maxATHB-14-likeb YGGGRQPAVL RTFSQRLCKG FNDAVNGFVD DGWS-LMGND GVEDVTIGIN
Pop_triXP_006378973.1 YGLGRQPAVL RTFSQRLSRG FNDAINGFND DGWS-LMNSD GAEDVIIAVN
Ory_satACA64837.1 YGAGRQPAVF RTFSQRLSRG FNDAVSGFPD DGWS-LLSSD GSEDITISVN
Vit_vin013992 YGLGRQPAVL RTFSQRLSRG FNDAINGFND DGWS-LMSCD GAEDVIIAVN
Ory_satOsJ_11861 YGGGRQPAVL RTFSQRLSRG FNDAVNGFPD DGWS-LMSSD GAEDVTIAFN
Sol_tubREVOLUTA-like YGLGRQPAVL RTFSQRLCRG FNDAINGFGD DGWS-MLSSD GAEDVIVAVN
Gly_maxATHB-14-likec YGGGRQPAVL RTFSQRLCKG FNDAVNGFVD DGWS-LMGND GVEDVTIAIN
The_cacXP_007024277.1 TGWGRRPAAL RALSQRLSRG FNEALNGFTD EGWS-MMGND GMDDVTILVN
Vit_vinATHB-15-like TGWGRRPAAL RALSQRLSRG FNEALNGFTD EGWS-MMGND GIDDVTILVN
Ric_comXP_002515977.1 TNWGRRPAAL RALSQRLSRG FNEALNGFTD EGWS-MMGND GMDDVTILVN
The_cacXP_007016751.1 YGLGRQPAVL RTFSQRLSRG FNDAINGFND DGWS-IMNCD GAEDVIIAIN
Sol_tubHOX32-like YSGGRQPAVL RALSQRLCRG FNDAVNGFVD DGWT-ITDSD GVEDVTIAIN
Sol_lycATHB-14-like YAGGRQPAVL RAFSQRLCRG FNNAVSGFID DGWT-IMGSD GVDDVTIAVN
Sol_tubATHB-14-like_X1 YAGGRQPAVL RAFSQRLCRG FNNAVSGFID DGWT-IMGSD GVDDVTIAVN
Med_truATHB-14 YGGGRQPAVL RTFSQRLCRG FNDAVNGFVD DGWS-LLGND GVEDVTIAVN
Zea_mayNP_001142394.1 YGAGRQPAVL RTFSQRLSRG FNDAVSGFPD DGWSCLLNTD GAEDITVTIN
Vit_vinREVOLUTA YGLGRQPAVL RTFSQRLSRG FNDAINGFND DGWS-LMSCD GAEDVIIAVN
Ory_braHOX32-like YGGGRQPAVL RTFSQRLSRG FNDAVNGFPD DGWS-PMSSD GAEDVTIAFN
Ric_comXP_002529946.1 YGLGRQPAVL RTFSQRLSRG FNDAINGFND DGWS-LMSCD GAEDVIVTIN
Tha_cacXP_007016749.1 YGLGRQPAVL RTFSQRLSRG FNDAINGFND DGWS-IMNCD GAEDVIIAIN
Fra_vesREVOLUTA-like YSLGRQPAVL RTFSQRLIRG FNDAVNGFND DGWS-LINCD GAEDIIMAVN
Zea_mayNP_001146215.1 YGAGRQPAVL RTFSQRLSRG FNDAVSGFPD DGWSSLLSSD GAEDISITIN
The_cacXP_007012152.1 TGWGRRPAAL RALSQKLSKG FNEAVNGFTD EGWS-MLESD GVDDVTLLVN
Pru_perppa001386mg AGWGRRPAAL RALSQRLSKG FNEAVNGFTD EGWS-ILESD GVDDVTLLVN
Pop_triAAX19057.1 TGWGRRPAAL RALSQRLSKG FNEAVNGFAD EGWS-MLESD GIDDVTVLVN
Pop_triXP_002309538.2 TGWGRRPAAL RALSQRLSKG FNEAVNGFAD EGWS-MLESD GIDDVTVLVN
Fra_vesATHB-8-like NGWGRRPAAL RALSQRLSKG FNEAVNGFTD EGWS-ILESD GVDDVTLLVN
Pop_triXP_002324794.1 TGWGRRPAAL RALSQRLSKG FNEAVNGFAD EGWS-LLESD GIDDVTVLVN
Vit_vin009744 TGWGRRPAAL RALGQRLTKG FNEAVNGFTD EGWS-MMESD GIDDVTLLVN
Gly_maxATHB-8-like TGWGRRPAAL RALSQRLSKG FNEAVNGFAD DGWS-MLESD GIDDVTLLVN
Sol_lycATHB-15-like SGWGRRPAAL RALGQRLSKG FNEAVNGFTD EGWS-MLESD GVDDVTILVN
Gly_maxATHB-8-likeb TGWGRRPAAL RALSQRLSKG FNEAVNGFAD DGWS-MLESD GIDDVTLLVN
Med_truXP_003603630.1 TGWGRRPAAL RALSQRLSKG FNEAVNGFTD DGWS-MLDSD GIDDVTLLVN

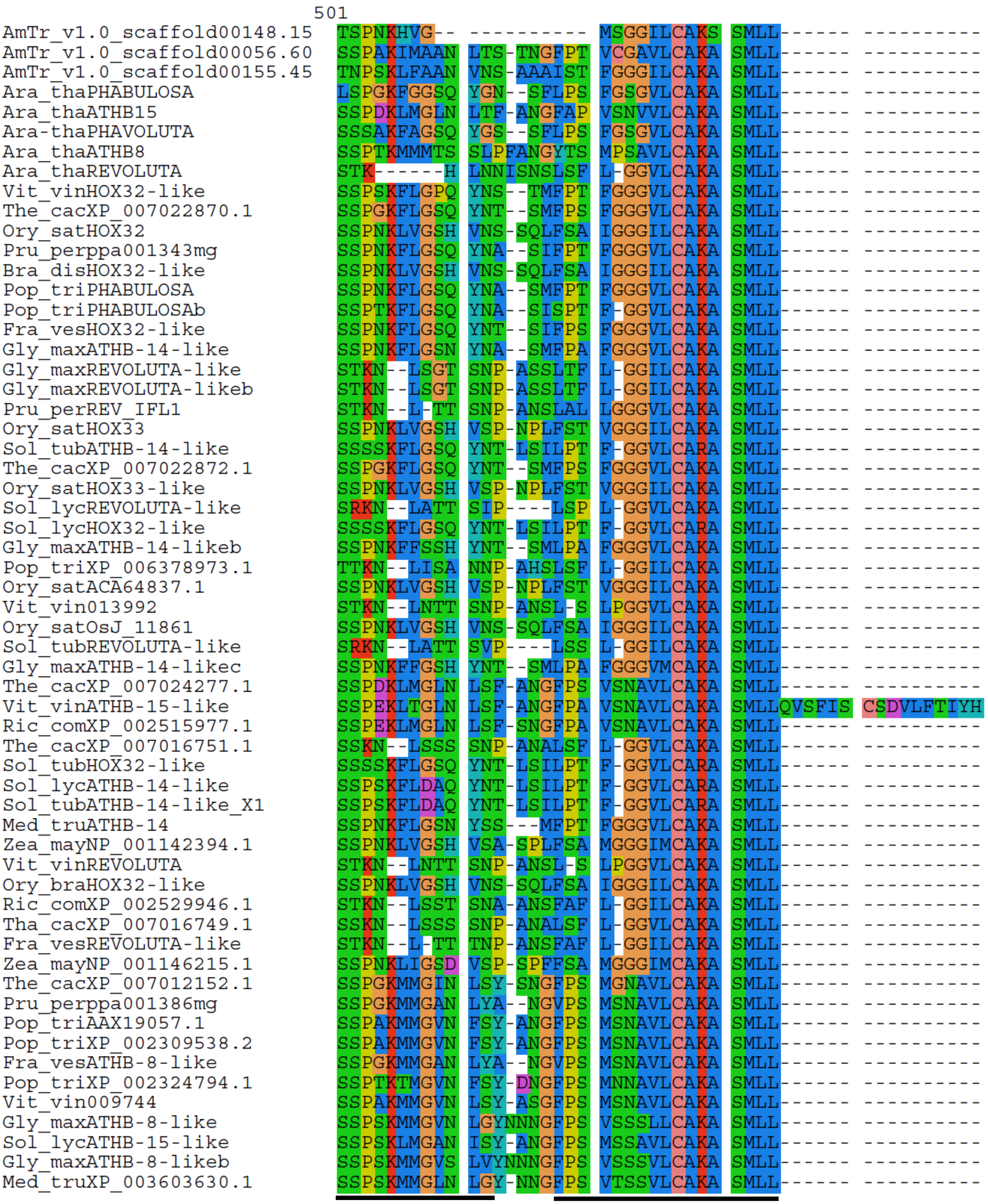
501
AmTr_v1.0_scaffold00148.15 TSPNKHVG-- ---------- MSGGILCAKS SMLL------ ----------
AmTr_v1.0_scaffold00056.60 SSPAKIMAAN LTS-TNGFPT VCGAVLCAKA SMLL------ ----------
AmTr_v1.0_scaffold00155.45 TNPSKLFAAN VNS-AAAIST FGGGILCAKA SMLL------ ----------
Ara_thaPHABULOSA LSPGKFGGSQ YGN--SFLPS FGSGVLCAKA SMLL------ ----------
Ara_thaATHB15 SSPDKLMGLN LTF-ANGFAP VSNVVLCAKA SMLL------ ----------
Ara-thaPHAVOLUTA SSSAKFAGSQ YGS--SFLPS FGSGVLCAKA SMLL------ ----------
Ara_thaATHB8 SSPTKMMMTS SLPFANGYTS MPSAVLCAKA SMLL------ ----------
Ara_thaREVOLUTA STK------H LNNISNSLSF L-GGVLCAKA SMLL------ ----------
Vit_vinHOX32-like SSPSKFLGPQ YNS--TMFPT FGGGVLCAKA SMLL------ ----------
The_cacXP_007022870.1 SSPGKFLGSQ YNT--SMFPS FGGGVLCAKA SMLL------ ----------
Ory_satHOX32 SSPNKLVGSH VNS-SQLFSA IGGGILCAKA SMLL------ ----------
Pru_perppa001343mg SSPNKFLGSQ YNA--SIFPT FGGGVLCAKA SMLL------ ----------
Bra_disHOX32-like SSPNKLVGSH VNS-SQLFSA IGGGILCAKA SMLL------ ----------
Pop_triPHABULOSA SSPNKFLGSQ YNA--SMFPT FGGGVLCAKA SMLL------ ----------
Pop_triPHABULOSAb SSPTKFLGSQ YNA--SISPT F-GGVLCAKA SMLL------ ----------
Fra_vesHOX32-like SSPNKFLGSQ YNT--SIFPS FGGGVLCAKA SMLL------ ----------
Gly_maxATHB-14-like SSPNKFLGSN YNA--SMFPA FGGGVLCAKA SMLL------ ----------
Gly_maxREVOLUTA-like STKN--LSGT SNP-ASSLTF L-GGILCAKA SMLL------ ----------
Gly_maxREVOLUTA-likeb STKN--LSGT SNP-ASSLTF L-GGILCAKA SMLL------ ----------
Pru_perREV_IFL1 STKN--L-TT SNP-ANSLAL LGGGVLCAKA SMLL------ ----------
Ory_satHOX33 SSPNKLVGSH VSP-NPLFST VGGGILCAKA SMLL------ ----------
Sol_tubATHB-14-like SSSSKFLGSQ YNT-LSILPT F-GGVLCARA SMLL------ ----------
The_cacXP_007022872.1 SSPGKFLGSQ YNT--SMFPS FGGGVLCAKA SMLL------ ----------
Ory_satHOX33-like SSPNKLVGSH VSP-NPLFST VGGGILCAKA SMLL------ ----------
Sol_lycREVOLUTA-like SRKN--LATT SIP----LSP L-GGVLCAKA SMLL------ ----------
Sol_lycHOX32-like SSSSKFLGSQ YNT-LSILPT F-GGVLCARA SMLL------ ----------
Gly_maxATHB-14-likeb SSPNKFFSSH YNT--SMLPA FGGGVLCAKA SMLL------ ----------
Pop_triXP_006378973.1 TTKN--LISA NNP-AHSLSF L-GGILCAKA SMLL------ ----------
Ory_satACA64837.1 SSPNKLVGSH VSP-NPLFST VGGGILCAKA SMLL------ ----------
Vit_vin013992 STKN--LNTT SNP-ANSL-S LPGGVLCAKA SMLL------ ----------
Ory_satOsJ_11861 SSPNKLVGSH VNS-SQLFSA IGGGILCAKA SMLL------ ----------
Sol_tubREVOLUTA-like SRKN--LATT SVP----LSS L-GGILCAKA SMLL------ ----------
Gly_maxATHB-14-likec SSPNKFFGSH YNT--SMLPA FGGGVMCAKA SMLL------ ----------
The_cacXP_007024277.1 SSPDKLMGLN LSF-ANGFPS VSNAVLCAKA SMLL------ ----------
Vit_vinATHB-15-like SSPEKLTGLN LSF-ANGFPA VSNAVLCAKA SMLLQVSFIS CSDVLFTIYH
Ric_comXP_002515977.1 SSPEKLMGLN LSF-SNGFPA VSNAVLCAKA SMLL------ ----------
The_cacXP_007016751.1 SSKN--LSSS SNP-ANALSF L-GGVLCAKA SMLL------ ----------
Sol_tubHOX32-like SSSSKFLGSQ YNT-LSILPT F-GGVLCARA SMLL------ ----------
Sol_lycATHB-14-like SSPSKFLDAQ YNT-LSILPT F-GGVLCARA SMLL------ ----------
Sol_tubATHB-14-like_X1 SSPSKFLDAQ YNT-LSILPT F-GGVLCARA SMLL------ ----------
Med_truATHB-14 SSPNKFLGSN YSS---MFPT FGGGVLCAKA SMLL------ ----------
Zea_mayNP_001142394.1 SSPNKLVGSH VSA-SPLFSA MGGGIMCAKA SMLL------ ----------
Vit_vinREVOLUTA STKN--LNTT SNP-ANSL-S LPGGVLCAKA SMLL------ ----------
Ory_braHOX32-like SSPNKLVGSH VNS-SQLFSA IGGGILCAKA SMLL------ ----------
Ric_comXP_002529946.1 STKN--LSST SNA-ANSFAF L-GGILCAKA SMLL------ ----------
Tha_cacXP_007016749.1 SSKN--LSSS SNP-ANALSF L-GGVLCAKA SMLL------ ----------
Fra_vesREVOLUTA-like STKN--L-TT TNP-ANSFAF L-GGILCAKA SMLL------ ----------
Zea_mayNP_001146215.1 SSPNKLIGSD VSP-SPFFSA MGGGIMCAKA SMLL------ ----------
The_cacXP_007012152.1 SSPGKMMGIN LSY-SNGFPS MGNAVLCAKA SMLL------ ----------
Pru_perppa001386mg SSPGKMMGAN LYA--NGVPS MSNAVLCAKA SMLL------ ----------
Pop_triAAX19057.1 SSPAKMMGVN FSY-ANGFPS MSNAVLCAKA SMLL------ ----------
Pop_triXP_002309538.2 SSPAKMMGVN FSY-ANGFPS MSNAVLCAKA SMLL------ ----------
Fra_vesATHB-8-like SSPGKMMGAN LYA--NGVPS MSNAVLCAKA SMLL------ ----------
Pop_triXP_002324794.1 SSPTKTMGVN FSY-DNGFPS MNNAVLCAKA SMLL------ ----------
Vit_vin009744 SSPAKMMGVN LSY-ASGFPS MSNAVLCAKA SMLL------ ----------
Gly_maxATHB-8-like SSPSKMMGVN LGYNNNGFPS VSSSLLCAKA SMLL------ ----------
Sol_lycATHB-15-like SSPSKLMGAN ISY-ANGFPS MSSAVLCAKA SMLL------ ----------
Gly_maxATHB-8-likeb SSPSKMMGVS LVYNNNGFPS VSSSVLCAKA SMLL------ ----------
Med_truXP_003603630.1 SSPSKMMGLN LGY-NNGFPS VTSSVLCAKA SMLL------ ----------

```
                             551
AmTr_v1.0_scaffold00148.15   -QNVPPALLI RFLREHRSEW ADSNIDAYCA ASLKANPFAI PGSGPSRFTG
AmTr_v1.0_scaffold00056.60   -QNVPPALLI RFLREHRSEW ADSNIDAYSA AALKSNPGTL PTSRMGGF-G
AmTr_v1.0_scaffold00155.45   -QNVPPALLV RFLREHRSEW ADSGVDAYAA SSMRTGQFVV PGSRTGSFPG
Ara_thaPHABULOSA             -QNVPPAVLV RFLREHRSEW ADYGVDAYAA ASLRASPFAV PCARAGGFPS
Ara_thaATHB15                -QNVPPAILL RFLREHRSEW ADNNIDAYLA AAVKVGPCS- --ARVGGF-G
Ara-thaPHAVOLUTA             -QNVPPLVLI RFLREHRAEW ADYGVDAYSA ASLRATPYAV PCVRTGGFPS
Ara_thaATHB8                 -QNVPPSILL RFLREHRQEW ADNSIDAYSA AAIKAGPCSL PIPRPGSF-G
Ara_thaREVOLUTA              -QNVPPAVLI RFLREHRSEW ADFNVDAYSA ATLKAGSFAY PGMRPTRFTG
Vit_vinHOX32-like            -QNVPPALLV RFLREHRSEW ADYGVDAYSA ACLKASPYEV PCARPGGFPS
The_cacXP_007022870.1        -QNVPPALLV RFLREHRSEW ADYGVDTYSA ACLKASPYAV PCARPGGFPS
Ory_satHOX32                 -QNVPPALLV RFLREHRSEW ADPGVDAYSA AALRASPYAV PGLRAGGFMG
Pru_perppa001343mg           -QSVPPALLV RFLREHRSEW ADYGVDAYSA ACLKASPYAV PCARPGGFPS
Bra_disHOX32-like            -QNVPPALLV RFLREHRSEW ADPGVDAYSA AALRASPYAV PGLRASGFMG
Pop_triPHABULOSA             -QNVPPALLV RFLREHRSEW ADYGVDAYSA ACLKASPYAV PCARPGGFPS
Pop_triPHABULOSAb            -QNVPPALLV RFLREHRSEW ADYGVDVYSA ACLKASPYAV PCARPGGFPS
Fra_vesHOX32-like            -QSVPPALLV RFLREHRSEW ADYGVDAYSA ACLKASPYAV PCARPGGFPS
Gly_maxATHB-14-like          -QNVPPALLV RFLREHRSEW ADYGVDAYSA ACLKASPYAV PCARPGGFPS
Gly_maxREVOLUTA-like         -QNVPPAVLV RFLREHRSEW ADFNVDAYSA ASLKAGTYAY PGMRPTRFTG
Gly_maxREVOLUTA-likeb        -QNVPPAVLV RFLREHRSEW ADFSVDAYSA ASLKAGTYAY PGMRPTRFTG
Pru_perREV_IFL1              -QNVPPAVLV RFLREHRSEW ADFNVDAYSA ASLKAGSYAY PGMRPTRFTG
Ory_satHOX33                 -QNVPPALLV RFLREHRSEW ADPGVDAYSA ASLRASPYAV PGLRTSGFMG
Sol_tubATHB-14-like          -QNVPPALLV RFLREHRSEW ADYGVDAYSS ASLKASPYAV PCARPGGFPS
The_cacXP_007022872.1        -QNVPPALLV RFLREHRSEW ADYGVDTYSA ACLKASPYAV PCARPGGFPS
Ory_satHOX33-like            -QNVPPALLV RFLREHRSEW ADPGVDAYSA ASLRASPYTV PGLRTSGFMG
Sol_lycREVOLUTA-like         -QNVPPAVLV RFLREHRSEW ADFNVDAFVA SALKSCPYTY PGMRPTRFTG
Sol_lycHOX32-like            -QNVPPALLV RFLREHRSEW ADYGVDAYSS ASLKASPYAV PCARPGGFPS
Gly_maxATHB-14-likeb         -QNVPPALLV RFLREHRSEW ANYGVDAYSS ACLKASPYAV PCARPSGFPS
Pop_triXP_006378973.1        -QNVPPAVLV RFLREHRSEW ADFSVDAYSA ASLKAGSYAY PGMRSMRFTG
Ory_satACA64837.1            -QNVPPALLV RFLREHRSEW ADPGVDAYSA ASLRASPYAV PGLRTSGFMG
Vit_vin013992                -QNVPPAVLV RFLREHRSEW ADFSVDAYSA ASLKASPYSY PGMRPTRFTG
Ory_satOsJ_11861             -QNVPPALLV RFLREHRSEW ADPGVDAYSA AALRASPYAV PGLRAGGFMG
Sol_tubREVOLUTA-like         -QNVPPAVLV RFLREHRSEW ADFNVDAFVA SALKSCPYTY PGMRPTRFTG
Gly_maxATHB-14-likec         -QNVPPALLV RFLREHRSEW ANYEVDAYSS ACLKASPYAV PCARPSGFPS
The_cacXP_007024277.1        -QNVPPAILL RFLREHRSEW ADSSIDAYSA AAVKVGPCSL PGSRVGGF-G
Vit_vinATHB-15-like          FQNVPPAILL RFLREHRSEW ADNNIDAYSA AAVKVGPCSL PGSRVGSF-G
Ric_comXP_002515977.1        -QNVPPAILL RFLREHRSEW ADNNIDAYSA AAIKVGPCTL PGTRIGSF-G
The_cacXP_007016751.1        -QNVPPAVLV RFLREHRSEW ADFNVDAYSA ASLKAGTYSY PGMRPTRFTG
Sol_tubHOX32-like            -QNVPPALLV RFLREHRSEW ADYGVDAYSS ASLKASPYAV PCARPGGFPS
Sol_lycATHB-14-like          -QDVCPALLV RFLREHRSEW ADYGVDAYSS ASLKASPYAV PCARPGVFPS
Sol_tubATHB-14-like_X1       -QDVCPALLV RFLREHRSEW ADYGVDAYSS ASLKASPYAV PCARPGVFPS
Med_truATHB-14               -QNVPPALLV RFLREHRSEW ADYGVDAYSA TCLKSSPYAV PCPRPGGFPS
Zea_mayNP_001142394.1        -QNVPPALLV RFLREHRSEW ADPGIDAYSV ASLRANPYTV PGLRAGGFMG
Vit_vinREVOLUTA              -QNVPPAVLV RFLREHRSEW ADFSVDAYSA ASLKASPYSY PGMRPTRFTG
Ory_braHOX32-like            -QNVPPALLV RFLREHRSEW ADPGVDAYSA AALRASPYAV PGLRAGGFMG
Ric_comXP_002529946.1        -QNVPPAVLV RFLREHRSEW ADFNVDAYSA ASLKAGSYAF PGMRPTRFTG
Tha_cacXP_007016749.1        -QNVPPAVLV RFLREHRSEW ADFNVDAYSA ASLKAGTYSY PGMRPTRFTG
Fra_vesREVOLUTA-like         -QNVPPAVLV RFLREHRSEW ADFNVDAYSA ASLKSGSYAY PGMRPTRFTG
Zea_mayNP_001146215.1        -QNVPPALLV RFLREHRSEW ADPGVDAYSA ASLRANPYNV PGLRAGGFMG
The_cacXP_007012152.1        -QNVPPAILL RFLREHRSEW ADSGIDAYSA AAVKAGPCSL PVSRGGSF-G
Pru_perppa001386mg           -QNVPPAILL RFLREHRSEW ADRSIDAYSA AAIKPGPCGL LGSRAGGF-G
Pop_triAAX19057.1            -QNVPPAILL RFLREHRSEW ADSGIDAYAA AAVKAGPCSL PMSRAGNF-G
Pop_triXP_002309538.2        -QNVPPAILL RFLREHRSEW ADSGIDAYAA AAVKAGPCSL PMSRAGNF-G
Fra_vesATHB-8-like           -QNVPPAILL RFLREHRSEW ADRSIDAYSA AAIKAGPCGM IGSRAGSF-G
Pop_triXP_002324794.1        -QNVPPAILL RFLREHRSEW ADNGIDAYAA SAIKAGPCSL PVSRAGNF-G
Vit_vin009744                -QNVPPAILL RFLREHRSEW ADSSIDAYSA AAVKAGPCTL PVSRAGGY-G
Gly_maxATHB-8-like           -QNVPPAILL RFLREHRSEW ADSSIDAYSA AAIKAGPCSL PGARPGGF-G
Sol_lycATHB-15-like          -QNVPPPILL RFLREHRSEW ADSGIDAYSA AAVKAGPCSI PVTRTGSF-G
Gly_maxATHB-8-likeb          -QNVPPAILL RFLREHRSEW ADSSIDAYSA AAIKAGPCSL PGARSGGF-G
Med_truXP_003603630.1        -QNVPPAILL RFLREHRSEW ADTSIDAYSA AAIKAGPCSL PGARTGAF-G
```

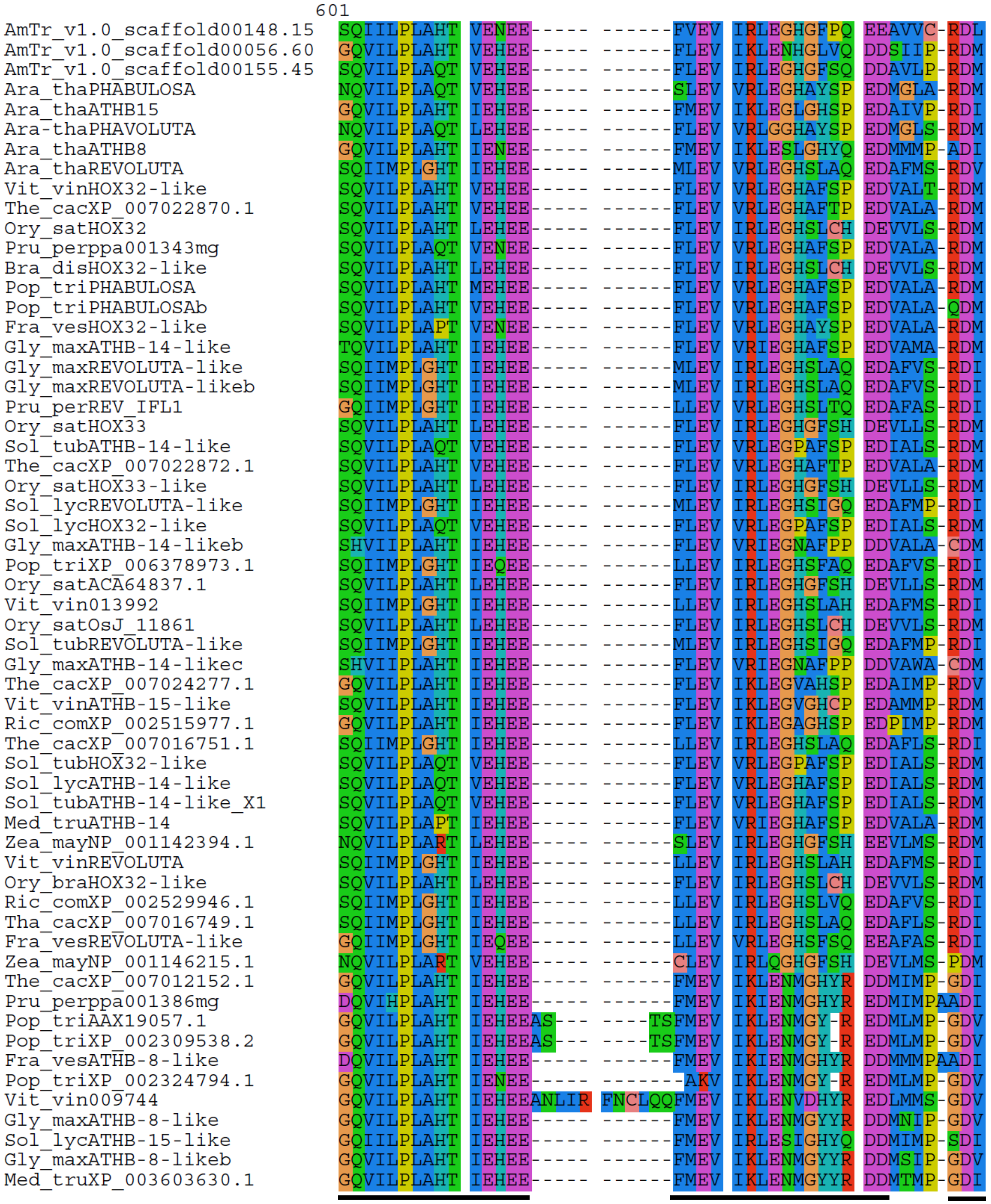
601
AmTr_v1.0_scaffold00148.15 SQIILPLAHT VENEE----- ------FVEV IRLEGHGFPQ EEAVVC-RDL
AmTr_v1.0_scaffold00056.60 GQVILPLAHT VEHEE----- ------FLEV IKLENHGLVQ DDSIIP-RDM
AmTr_v1.0_scaffold00155.45 SQVILPLAQT VEHEE----- ------FLEV IRLEGHGFSQ DDAVLP-RDM
Ara_thaPHABULOSA NQVILPLAQT VEHEE----- ------SLEV VRLEGHAYSP EDMGLA-RDM
Ara_thaATHB15 GQVILPLAHT IEHEE----- ------FMEV IKLEGLGHSP EDAIVP-RDI
Ara-thaPHAVOLUTA NQVILPLAQT LEHEE----- ------FLEV VRLGGHAYSP EDMGLS-RDM
Ara_thaATHB8 GQVILPLAHT IENEE----- ------FMEV IKLESLGHYQ EDMMMP-ADI
Ara_thaREVOLUTA SQIIMPLGHT IEHEE----- ------MLEV VRLEGHSLAQ EDAFMS-RDV
Vit_vinHOX32-like SQVILPLAHT VEHEE----- ------FLEV VRLEGHAFSP EDVALT-RDM
The_cacXP_007022870.1 SQVILPLAHT VEHEE----- ------FLEV VRLEGHAFTP EDVALA-RDM
Ory_satHOX32 SQVILPLAHT LEHEE----- ------FLEV IRLEGHSLCH DEVVLS-RDM
Pru_perppa001343mg SQVILPLAQT VENEE----- ------FLEV VRLEGHAFSP EDVALA-RDM
Bra_disHOX32-like SQVILPLAHT LEHEE----- ------FLEV IRLEGHSLCH DEVVLS-RDM
Pop_triPHABULOSA SQVILPLAHT MEHEE----- ------FLEV VRLEGHAFSP EDVALA-RDM
Pop_triPHABULOSAb SQVILPLAHT VEHEE----- ------FLEV VRLEGHAFSP EDVALA-QDM
Fra_vesHOX32-like SQVILPLAPT VENEE----- ------FLEV VRLEGHAYSP EDVALA-RDM
Gly_maxATHB-14-like TQVILPLAHT IEHEE----- ------FLEV VRIEGHAFSP EDVAMA-RDM
Gly_maxREVOLUTA-like SQIIMPLGHT IEHEE----- ------MLEV IRLEGHSLAQ EDAFVS-RDI
Gly_maxREVOLUTA-likeb SQIIMPLGHT IEHEE----- ------MLEV IRLEGHSLAQ EDAFVS-RDI
Pru_perREV_IFL1 GQIIMPLGHT IEHEE----- ------LLEV VRLEGHSLTQ EDAFAS-RDI
Ory_satHOX33 SQVILPLAHT LEHEE----- ------FLEV IRLEGHGFSH DEVLLS-RDM
Sol_tubATHB-14-like SQVILPLAQT VEHEE----- ------FLEV VRLEGPAFSP EDIALS-RDM
The_cacXP_007022872.1 SQVILPLAHT VEHEE----- ------FLEV VRLEGHAFTP EDVALA-RDM
Ory_satHOX33-like SQVILPLAHT LEHEE----- ------FLEV IRLEGHGFSH DEVLLS-RDM
Sol_lycREVOLUTA-like SQIIMPLGHT IEHEE----- ------MLEV IRLEGHSIGQ EDAFMP-RDI
Sol_lycHOX32-like SQVILPLAQT VEHEE----- ------FLEV VRLEGPAFSP EDIALS-RDM
Gly_maxATHB-14-likeb SHVIIPLAHT IEHEE----- ------FLEV VRIEGNAFPP DDVALA-CDM
Pop_triXP_006378973.1 SQIIMPLGHT IEQEE----- ------LLEV IRLEGHSFAQ EDAFVS-RDI
Ory_satACA64837.1 SQVILPLAHT LEHEE----- ------FLEV IRLEGHGFSH DEVLLS-RDM
Vit_vin013992 SQIIMPLGHT IEHEE----- ------LLEV IRLEGHSLAH EDAFMS-RDI
Ory_satOsJ_11861 SQVILPLAHT LEHEE----- ------FLEV IRLEGHSLCH DEVVLS-RDM
Sol_tubREVOLUTA-like SQIIMPLGHT IEHEE----- ------MLEV IRLEGHSIGQ EDAFMP-RDI
Gly_maxATHB-14-likec SHVIIPLAHT IEHEE----- ------FLEV VRIEGNAFPP DDVAWA-CDM
The_cacXP_007024277.1 GQVILPLAHT IEHEE----- ------FLEV IKLEGVAHSP EDAIMP-RDV
Vit_vinATHB-15-like SQVILPLAHT IEHEE----- ------FLEV IKLEGVGHCP EDAMMP-RDM
Ric_comXP_002515977.1 GQVILPLAHT IEHEE----- ------FLEV IKLEGAGHSP EDPIMP-RDM
The_cacXP_007016751.1 SQIIMPLGHT IEHEE----- ------LLEV IRLEGHSLAQ EDAFLS-RDI
Sol_tubHOX32-like SQVILPLAQT VEHEE----- ------FLEV VRLEGPAFSP EDIALS-RDM
Sol_lycATHB-14-like SQVILPLAQT VEHEE----- ------FLEV VRLEGHAFSP EDIALS-RDM
Sol_tubATHB-14-like_X1 SQVILPLAQT VEHEE----- ------FLEV VRLEGHAFSP EDIALS-RDM
Med_truATHB-14 SQVILPLAPT IEHEE----- ------FLEV VRIEGHAFSP EDVALA-RDM
Zea_mayNP_001142394.1 NQVILPLART LEHEE----- ------SLEV IRLEGHGFSH EEVLMS-RDM
Vit_vinREVOLUTA SQIIMPLGHT IEHEE----- ------LLEV IRLEGHSLAH EDAFMS-RDI
Ory_braHOX32-like SQVILPLAHT LEHEE----- ------FLEV IRLEGHSLCH DEVVLS-RDM
Ric_comXP_002529946.1 SQIIMPLGHT IEHEE----- ------LLEV IRLEGHSLVQ EDAFVS-RDI
Tha_cacXP_007016749.1 SQIIMPLGHT IEHEE----- ------LLEV IRLEGHSLAQ EDAFLS-RDI
Fra_vesREVOLUTA-like GQIIMPLGHT IEQEE----- ------LLEV VRLEGHSFSQ EEAFAS-RDI
Zea_mayNP_001146215.1 NQVILPLART VEHEE----- ------CLEV IRLQGHGFSH DEVLMS-PDM
The_cacXP_007012152.1 GQVILPLAHT IEHEE----- ------FMEV IKLENMGHYR DDMIMP-GDI
Pru_perppa001386mg DQVIHPLAHT IEHEE----- ------FMEV IKIENMGHYR EDMIMPAADI
Pop_triAAX19057.1 GQVILPLAHT IEHEEAS--- ----TSFMEV IKLENMGY-R EDMLMP-GDV
Pop_triXP_002309538.2 GQVILPLAHT IEHEEAS--- ----TSFMEV IKLENMGY-R EDMLMP-GDV
Fra_vesATHB-8-like DQVILPLAHT IEHEE----- ------FMEV IKIENMGHYR DDMMMPAADI
Pop_triXP_002324794.1 GQVILPLAHT IENEE----- -------AKV IKLENMGY-R EDMLMP-GDV
Vit_vin009744 GQVILPLAHT IEHEEANLIR FNCLQQFMEV IKLENVDHYR EDLMMS-GDV
Gly_maxATHB-8-like GQVILPLAHT IEHEE----- ------FMEV IKLENMGYYR DDMNIP-GDV
Sol_lycATHB-15-like GQIILPLAHT IEHEE----- ------FMEV IRLESIGHYQ DDMIMP-SDI
Gly_maxATHB-8-likeb GQVILPLAHT IEHEE----- ------FMEV IKLENMGYYR DDMSIP-GDV
Med_truXP_003603630.1 GQVILPLAHT IEHEE----- ------FMEV IKLENMGYYR DDMTMP-GDI

```
                                651
AmTr_v1.0_scaffold00148.15     YLLQLCSGVD ENAVGAISQL VFAPIDDSFA DDAPLLPSGF RVIPLDSKT-
AmTr_v1.0_scaffold00056.60     FLLQLCSGVD ENAVGACAEL VFAPIDPSFA DDAPLLPSGF RIIPLDNGV-
AmTr_v1.0_scaffold00155.45     FLLQLCSGVD ENAVGACAQL VFAPIDESFA DDAPLLPSGF RVIPLDSKS-
Ara_thaPHABULOSA               YLLQLCSGVD ENVVGGCAQL VFAPIDESFA DDAPLLPSGF RIIPLEQKS-
Ara_thaATHB15                  FLLQLCSGMD ENAVGTCAEL IFAPIDASFA DDAPLLPSGF RIIPLDSAK-
Ara-thaPHAVOLUTA               YLLQLCSGVD ENVVGGCAQL VFAPIDESFA DDAPLLPSGF RVIPLDQKT-
Ara_thaATHB8                   FLLQMCSGVD ENAVESCAEL IFAPIDASFS DDAPIIPSGF RIIPLDSKS-
Ara_thaREVOLUTA                HLLQICTGID ENAVGACSEL IFAPINEMFP DDAPLVPSGF RVIPVDAKT-
Vit_vinHOX32-like              YLLQLCSGVD ENAAGACAQL VFAPIDESFA DDAPLLPSGF RVIPLDPKT-
The_cacXP_007022870.1          YLLQLCSGID ENAVGACAQL VFAPIDESFA DDAPLLPSGF RVIPLDPKTV
Ory_satHOX32                   YLLQLCSGVD ENAAGACAQL VFAPIDESFA DDAPLLPSGF RVIPLDGKT-
Pru_perppa001343mg             YLLQLCSGVD ENAVGSCAQL VFAPIDESFA DDAPLLPSGF HVIPLDPKT-
Bra_disHOX32-like              YLLQLCSGVD ENAAGACAQL VFAPIDESFA DDAPLLPSGF RVIPLDAKT-
Pop_triPHABULOSA               YLLQLCSGVD ENAVGACAQL VFAPIDESFA DDAPLLSSGF RVIPLDPKT-
Pop_triPHABULOSAb              YLLQLCSGVD ENALGACAQL VFAPIDESFA DDAPLLPSGF RVIPLDPKT-
Fra_vesHOX32-like              FLLQMCSGVD ENAVGSCAQL VFAPIDESFA DDAPLLPSGF HVIPLDAKT-
Gly_maxATHB-14-like            YLLQLCSGVD ESAVGACAQL VFAPIDESFA DDALLLPSGF RVIPLDPKS-
Gly_maxREVOLUTA-like           HLLQICSGID ENAVGACSEL VFAPIDEMFP DDAPLVPSGF RIIPLDSKP-
Gly_maxREVOLUTA-likeb          HLLQICSGID ENAVGACSEL VFAPIDEMFP DDAPLIPSGF RIIPLDSKP-
Pru_perREV_IFL1                HLLQICSGVD ENAVGACSEL VFAPIDEMFP DDAPLVPSGF RIIPLDSKT-
Ory_satHOX33                   YLLQLCSGVD ENATSASAQL VFAPIDESFA DDAPLLPSGF RVIPLDTKM-
Sol_tubATHB-14-like            YLLQLCSGVD ENAAGACAQL VFAPIDESFG DDAPLIPSGF RVIPLEPKS-
The_cacXP_007022872.1          YLLQLCSGID ENAVGACAQL VFAPIDESFA DDAPLLPSGF RVIPLDPKTV
Ory_satHOX33-like              YLLQLCSGVD ENATSASAQL VFAPIDESFA DDAPLLPSGF RVIPLDTKM-
Sol_lycREVOLUTA-like           HLLQMCSGTD ENAVGACSEL VFAPIDEMFP DDAPLLPSGF RIIPLESKS-
Sol_lycHOX32-like              YLLQLCSGVD ENAAGACAQL VFAPIDESFG DDAPLLPSGF RVIPLEPKS-
Gly_maxATHB-14-likeb           YLMQLCSGID ENAIGACAQL VFAPIDESFA DDALLLPSGF RIIPLDPKT-
Pop_triXP_006378973.1          HLLQICSGID ENAVGACSEL VFAPIDEMFP DDAPLLPSGF RVIPLESKT-
Ory_satACA64837.1              YLLQLCSGVD ENATSASAQL VFAPIDESFA DDAPLLPSGF RVIPLDTKM-
Vit_vin013992                  HLLQICSGVD ENAVGACSEL VFAPIDEMFP DDAPLLPSGF RIIPLDSKS-
Ory_satOsJ_11861               YLLQLCSGVD ENAAGACAQL VFAPIDESFA DDAPLLPSGF RVIPLDGKT-
Sol_tubREVOLUTA-like           HLLQMCSGTD ENAVGACSEL VFAPIDEMFP DDAPLLPSGF RIIPLESKS-
Gly_maxATHB-14-likec           YLMQLCSGID ENAIGACAQL VFAPIDESFA DDALLLPSGF RIIPLDPKT-
The_cacXP_007024277.1          FLLQLCSGMD ENAVGTCAEL IFAPIDASFA DDAPLLPSGF RIIPLDSGK-
Vit_vinATHB-15-like            FLLQLCSGMD ENAVGTCAEL IFAPIDASFA DDAPLLPSGF RIIPLDSGK-
Ric_comXP_002515977.1          FLLQLCSGMD ENAVGTCAEL IFAPIDASFA DDAPLLPSGF RIIPLDSAK-
The_cacXP_007016751.1          HLLQICSGID ENAVGACSEL VFAPIDEMFP DDAPLLPSGF RIIPLDSKP-
Sol_tubHOX32-like              YLLQLCSGVD ENAAGACAQL VFAPIDESFG DDAPLLPSGF RVIPLEPKS-
Sol_lycATHB-14-like            YLLQLCSGVE ENATGACAQL VFAHIDESFG DDAPLLPSGF SVIPLEPKS-
Sol_tubATHB-14-like_X1         YLLQLCSGVE ENATGACAQL VFAHIDESFG DDAPLLPSGF SVIPLEPKS-
Med_truATHB-14                 YLLQLCSGID ENSVGACAQL VFAPIDESFA DDALLLPSGF RVIPLDPKS-
Zea_mayNP_001142394.1          FLLQLCSGVD EDAPGACAQL VFAPIDESFA DDAPLLPSGF RVIPLDAKT-
Vit_vinREVOLUTA                HLLQICSGVD ENAVGACSEL VFAPIDEMFP DDAPLLPSGF RIIPLDSKS-
Ory_braHOX32-like              YLLQLCSGVD ENAAGACAQL VFAPIDESFA DDAPLLPSGF RVIPLDAKA-
Ric_comXP_002529946.1          HLLQICSGID ENAVGACSEL VFAPIDEMFP DDAPLLPSGF RIIPLDSKT-
Tha_cacXP_007016749.1          HLLQICSGID ENAVGACSEL VFAPIDEMFP DDAPLLPSGF RIIPLDSKP-
Fra_vesREVOLUTA-like           HLLQICSGVD ENAVGACSEL FFAPIDEMFP DDAPLLPSGF RIIPLESKT-
Zea_mayNP_001146215.1          FLLQLCSGID EDAPGACAQL VFAPIDESFA DDAPLLPSGF RVIPLDAKT-
The_cacXP_007012152.1          FLLQLCSGVD ENAVGTCAEL IFAPIDASFS DDAPIIPSGF RIIPLDSGM-
Pru_perppa001386mg             FLLQLCSGVD ENSVGTCAEL VFAPIDASFS DDGPILPSGF RIIPLDSRM-
Pop_triAAX19057.1              FLLQLCSGVD ENAVGTCAEL IFAPIDASFS DDAPIIPSGF RIIPLDSGM-
Pop_triXP_002309538.2          FLLQLCSGVD ENAVGTCAEL IFAPIDASFS DDAPIIPSGF RIIPLDSGM-
Fra_vesATHB-8-like             FLLQLCSGVD ENAVGTCAEL VFAPIDASFS DDAPILPSGF RIIPLDSHM-
Pop_triXP_002324794.1          FLLQLCSGVD ENAVGTCAEL IFAPIDASFS DDAPIIPSGF RIIPLDSGM-
Vit_vin009744                  FLLQLCSGVD DNAVGTCSEL IFAPIDASFS DDAPLLPSGF RIIPLDSGV-
Gly_maxATHB-8-like             FLLQLCSGVD EHAVGTSAEL VFAPIDASFS DDAPILPSGF RIIPLDSGT-
Sol_lycATHB-15-like            FLLQLCNGVD ENAIGTCAEL MFAPIDASFA DDAPLLPSGF RIIPLDSKA-
Gly_maxATHB-8-likeb            FLLQLCSGVD EHAVGTSAEL VFAPIDASFS DDAPILPSGF RIIPLDSGT-
Med_truXP_003603630.1          FLLQLCSGVD EHAVGTSAEL IFAPIDASFS DDAPILPSGF RIIPLESGT-
```

```
                              701
AmTr_v1.0_scaffold00148.15    ----NPQDNT AAA-IRTLDL TSSLDIGSA- -TNRASGDGG GPNSCNNLRS
AmTr_v1.0_scaffold00056.60    --------DG SSP-NRTLDL ASALEVGPA- -GNRISGEFA GGSGS--LRS
AmTr_v1.0_scaffold00155.45    --------DG PGT-NRTLDL ASSIDVGST- -GSRPSGESG A-NGY-TMRS
Ara_thaPHABULOSA              ------TPNG ASA-NRTLDL ASALE---G- -STRQAGEAD P-NGC-NFRS
Ara_thaATHB15                 --------EV SSP-NRTLDL ASALEIGSA- -GTKASTDQS GNSTC--ARS
Ara-thaPHAVOLUTA              -----NPNDH QSA-SRTRDL ASSLD---G- -STKTDSET- ------NSRL
Ara_thaATHB8                  --------EG LSP-NRTLDL ASALDVG--- --SRTAGDSC GSRGN--SKS
Ara_thaREVOLUTA               ----GDVQDL LTANHRTLDL TSSLEVGPS- -PENASGNS- --FSSSSSRC
Vit_vinHOX32-like             --------DG PAA-TRTLDL ASTLEVGAG- -GARPANESD L-NNY-NLRS
The_cacXP_007022870.1         TLTCQSSADG AGA-TRTLDL ASALEVGPG- -SNRLAGDGD P-SNY-NLRS
Ory_satHOX32                  --------DA PSA-TRTLDL ASTLEVGSG- -GTTRASSDT S-STC-NTRS
Pru_perppa001343mg            --------DG PTA-NRTLDL ASTLEVGPS- -GSRPVNEAD G-NSY-NLRS
Bra_disHOX32-like             --------DA PSA-TRTLDL ASTLEVGSG- -GTRAASDA- --PSTSNTRS
Pop_triPHABULOSA              --------DA PAT-TRTLDL ASTLEVGPG- -GTRPASEAD T-NSY-NLRS
Pop_triPHABULOSAb             --------DA PAA-TRTLDL ASTLEVGPG- -GARPASEAD T-NSY-NLRS
Fra_vesHOX32-like             --------DG PAT-NRTLDL ASTLEVGAS- -SARRVNEGD A-NSY-NLRS
Gly_maxATHB-14-like           --------DG PAP-TRTLDL ASTMEVGSG- -NARPAGEAD L-NGY-NLRS
Gly_maxREVOLUTA-like          ----GDKKDA VAT-NRTLDL TSGFEVGPA- --TTAGADA- --SSSQNTRS
Gly_maxREVOLUTA-likeb         ----GDKKEV ATN--RTLDL TSGFEVGPA- --TTAGTDA- --SSSQNTRS
Pru_perREV_IFL1               ----SDSKDT LAT-HRTLDL TSSLEVGST- -TNNAAGEL- --SSFHNTRS
Ory_satHOX33                  --------DG PSA-TRTLDL ASALEVGPG- -GASRASVEA S-GTC--NRS
Sol_tubATHB-14-like           --------DV PAA-TRTLDL ASTLEAGTGG SGTRPAGEIE A-GNY-NHRS
The_cacXP_007022872.1         TLTCQSSADG AGA-TRTLDL ASALEVGPG- -SNRLAGDGD P-SNY-NLRS
Ory_satHOX33-like             --------DV PSA-TRTLDL ASALEVGPG- -GASRASTDA S-GVC--NRS
Sol_lycREVOLUTA-like          ----GDAQDT LNA-HRTLDL ASSLEVGPA- -TNSTTGDA- --ASCYSARS
Sol_lycHOX32-like             --------DV PAA-TRTLDL ASTLEAGTGG SGTRPAGEIE A-GNY-NHRS
Gly_maxATHB-14-likeb          --------DG PAS-TRTLDL ASTLETGSG- -NARSAGESD L-NNY-NLRS
Pop_triXP_006378973.1         ----KDAQEA LTT-NRTLDL TSSLEVGPV- -TNHASVDG- --SSC-HLRS
Ory_satACA64837.1             --------DG PSA-TRTLDL ASALEVGPG- -GASRASVEA S-GTC--NRS
Vit_vin013992                 ----GDTQET LTT-HRTLDL TSSLEVGPA- -TNQAAGDS- --SSCYNTRS
Ory_satOsJ_11861              --------DA PSA-TRTLDL ASTLEVGSG- -GTTRASSDT S-STC-NTRS
Sol_tubREVOLUTA-like          ----GDAQDT LNA-HRTLDL ASSLEVGPA- -RNSATGDM- --ASCYSARS
Gly_maxATHB-14-likec          --------DG LAS-TRTLDL ASTLETGSG- -NARSAGESD S-NNY-NLRS
The_cacXP_007024277.1         --------EA SSP-NRTLDL ASALEIGPT- -GNKASNDYS GNSGC--MRS
Vit_vinATHB-15-like           --------EA SSP-NRTLDL ASALEIGPA- -GNRSSNDYS VNGGN--TRS
Ric_comXP_002515977.1         --------EA SSP-NRTLDL ASALEIGPA- -GNKSSTDYS SNSGC--MRS
The_cacXP_007016751.1         ----SDTQDS LTT-NRTLDL TSSLEVGTA- -TNHAAGDA- --PSCQNSRS
Sol_tubHOX32-like             --------DV PAA-TRTLDL ASTLEAGTGG SGTRPAGEIE A-GNY-NHRS
Sol_lycATHB-14-like           --------DS PSA-ARTLDL ASTLEARTG- -GTRPAGEVE G-SSY-NHRS
Sol_tubATHB-14-like_X1        --------DS PSA-ARTLDL ASTLEARTG- -GTRLAGEVE G-RSY-NHRS
Med_truATHB-14                --------DG PTT-SRTLDL LE----GSR- -NARPSGEGA GSNGY-NLRS
Zea_mayNP_001142394.1         --------DV PTATTRTLDL ASALEVGSG- -GSGMRASCD GSGTC-ATRS
Vit_vinREVOLUTA               ----GDTQET LTT-HRTLDL TSSLEVGPA- -TNQAAGDS- --SSCYNTRS
Ory_braHOX32-like             --------DA PSA-TRTLDL ASTLEVGSG- -GTTRASSDT S-GTC-NTRS
Ric_comXP_002529946.1         ----KDTQDA LTT-SRTLDL TSSLEVGPA- -ANNTAGDA- --SSSQSTRS
Tha_cacXP_007016749.1         ----SDTQDS LTT-NRTLDL TSSLEVGTA- -TNHAAGDA- --PSCQNSRS
Fra_vesREVOLUTA-like          ----SDSKDA LAT-HRTLDL TSSLEVGST- -TNHNAGDL- --NSFHNTRS
Zea_mayNP_001146215.1         --------DV PSA-TRTLDL ASALEVGSG- -GGLCALSDS G-SGTRSTRS
The_cacXP_007012152.1         --------DA SSP-NRTLDL ASTLEVGAA- -GNRATGDHS GRCGS--TKS
Pru_perppa001386mg            --------DA PSP-NRTLDL ASALEVGPA- -GSRASGDNA GHSGN--TKS
Pop_triAAX19057.1             --------DA SSP-NRTLDL ASALEVGPA- -GNRASGDLS GRSGC--TKS
Pop_triXP_002309538.2         --------DA SSP-NRTLDL ASALEVGPA- -GNRASGDLS GRSGC--TKS
Fra_vesATHB-8-like            --------DA PSP-NRTLDL ASALEVGPT- -GNRSSGDIA GHCGS--TKS
Pop_triXP_002324794.1         --------DA SSP-NRTLDL ASALEVGPA- -GNRASGDLS GRSGC--TKS
Vit_vin009744                 --------DG SSP-NRTLDL ASSLEVGPA- -GNKASSDNS GHTGS--AKS
Gly_maxATHB-8-like            --------DA ASP-NRTLDL ASALEVGTT- -ANKAAGDNS GHSGS--TKS
Sol_lycATHB-15-like           --------DA SSP-NRTLDL ASTLEVGPA- -GSRPTGDHS KNSGS--AKS
Gly_maxATHB-8-likeb           --------DA ASP-NRTLDL ASALEVGTT- -ANKAASDNS AHSGS--TKS
Med_truXP_003603630.1         --------DA ASP-NRTLDL ASALEVGTT- -GNKAGGDNS GHSGI--TKS
```

	751					
AmTr_v1.0_scaffold00148.15	VLTIAFQFTF	EPH---MRDN	VVAMARQYVR	GVVGSVQRVA	MAISPSRLGA	
AmTr_v1.0_scaffold00056.60	VLTIAFQFSY	ENNHEQIRDN	VATMARQYVR	SVISSVQRVA	MALTPSRLNP	
AmTr_v1.0_scaffold00155.45	VLTIAFQFTY	ETH---LRDN	VVAMARQYVR	SVMASVQRVA	MAISPSRMGP	
Ara_thaPHABULOSA	VLTIAFQFTF	DNH---SRDS	VASMARQYVR	SIVGSIQRVA	LAIAP-RPGS	
Ara_thaATHB15	VMTIAFEFGI	ESH---MQEH	VASMARQYVR	GIISSVQRVA	LALSPSHISS	
Ara-thaPHAVOLUTA	VLTIAFQFTF	DNH---SRDN	VATMARQYVR	NVVGSIQRVA	LAITPRP---	
Ara_thaATHB8	VMTIAFQLAF	EMH---MQEN	VASMARQYVR	SVIASVQRVA	LALSPSSH-Q	
Ara_thaREVOLUTA	ILTIAFQFPF	ENN---LQEN	VAGMACQYVR	SVISSVQRVA	MAISPSGISP	
Vit_vinHOX32-like	VLTIAFQFTF	ENH---VRDN	VAAMARQYVR	SVMASVQRVA	MAIAPSRLSS	
The_cacXP_007022870.1	VLTIAFQFTF	ENH---LRDN	VAAMARQYVR	SVVGSVQRVA	MAIAPSRLSS	
Ory_satHOX32	VLTIAFQFSY	ENH---LRES	VAAMARQYVR	TVVASVQRVA	MAIAPSRLGG	
Pru_perppa001343mg	VLTIAFQFTF	ENH---LRDN	VAAMARQYVR	SVVSSVQRVA	MAIAPSRLSS	
Bra_disHOX32-like	VLTIAFQFSY	ENH---LRES	VASMARQYVR	TVVASVQRVA	MAIAPSRLGG	
Pop_triPHABULOSA	VLTIAFQFSF	ENH---MRDN	VAAMARQYVR	GVVGSVQRVA	MAIAPSRLSS	
Pop_triPHABULOSAb	VLTIAFQFTF	ENH---FRDN	VAAMARQYVR	GVVASVQRVA	MAISPSRLSS	
Fra_vesHOX32-like	VLTIAFQFTF	ENH---MRDS	VAAMARQYVR	GVVSSVQRVA	MAIAPSRLSS	
Gly_maxATHB-14-like	VLTIAFQFTF	ENH---TRDN	VAAMARQYVR	SVVGSVQRVA	MAIAPSRLNT	
Gly_maxREVOLUTA-like	VLTIAFQFPF	DSS---LQDN	VAVMARQYVR	SVISSVQRVA	MAISPSGINP	
Gly_maxREVOLUTA-likeb	VLTIAFQFPF	DSS---LQDN	VAVMARQYVR	SVISSVQRVA	MAISPSGISP	
Pru_perREV_IFL1	VLTIAFQFPF	ENS---LQEN	VATMARQYVR	SVISSVQRVA	MAISPSGLSP	
Ory_satHOX33	VLTIAFQFSY	ENH---LRES	VAAMARSYVR	AVMASVQRVA	VAIAPSRLGP	
Sol_tubATHB-14-like	VLTIAFQFTF	ESH---YQDN	VAAMARQYVR	SIVGSVQRVA	MAIAPSRLSS	
The_cacXP_007022872.1	VLTIAFQFTF	ENH---LRDN	VAAMARQYVR	SVVGSVQRVA	MAIAPSRLSS	
Ory_satHOX33-like	VLTIAFQFSY	ENH---LRDS	VAAMARNYVR	AVMASVQRVA	VAIAPSRLGP	
Sol_lycREVOLUTA-like	VLTIAFQFPF	EDN---LQDN	VATMARQYVR	SVVSSVQRVA	MAISPTGMNP	
Sol_lycHOX32-like	VLTISFQFTF	ESH---YQDN	VAAMARQYVR	SIVGSVQRVA	MAIAPSRLSS	
Gly_maxATHB-14-likeb	VLTIAFQFTF	ENH---LRDN	VAVMARQYVR	NVVRSVQRVA	MAIAPSRIST	
Pop_triXP_006378973.1	VLTIAFQFPF	ESN---LQDN	VATMARQYVR	SVISSVQRVA	TAISPSGLNP	
Ory_satACA64837.1	VLTIAFQFSY	ENH---LRES	VAAMARSYVR	AVMASVQRVA	VAIAPSRLGP	
Vit_vin013992	VLTIAFQFPF	ESN---LQDN	VATMARQYVR	SVISSVQRVA	MAISPSGLGP	
Ory_satOsJ_11861	VLTIAFQFSY	ENH---LRES	VAAMARQYVR	TVVASVQRVA	MAIAPSRLGG	
Sol_tubREVOLUTA-like	VLTIAFQFPF	EDN---LQDN	VATMARQYVR	SVVSSVQRVA	MAISPTGMNP	
Gly_maxATHB-14-likec	VLTIAFQFTF	ENH---LRDN	VAVMARQYVR	NVVRSVQRVA	MAIAPSRLST	
The_cacXP_007024277.1	VMTIAFEFAF	ESH---MQEH	VASMARQYVR	SIISSVQRVA	LALSPSHLSS	
Vit_vinATHB-15-like	VMTIAFEFAF	ESH---LQEN	VASMARQYVR	SIISSVQRVA	LALSPSHLSS	
Ric_comXP_002515977.1	VMTIAFEFAF	ESH---MQEH	VASMARQYVR	SIISSVQRVA	LALSPSHSGS	
The_cacXP_007016751.1	VLTIALQFPF	DSN---LQDN	VAAMARQYVR	SVIASVQRVA	MAISPSGLSP	
Sol_tubHOX32-like	VLTISFQFTF	ESH---YQDN	VAAMARQYVR	SIVGSVQRVA	MAIAPSRLSS	
Sol_lycATHB-14-like	VLTIAFQFAF	ENH---YREN	VAAMARQYVR	SIVGSVQRVA	MAIAPSRLCS	
Sol_tubATHB-14-like_X1	VLTIAFQFAF	ENH---YREN	VAAMARQYVR	SIVGSVQRVA	MAIAPSRLCS	
Med_truATHB-14	VLTIAFQFTF	ENH---LRDN	VASMARQYVR	SVVASVQRVA	MAIAPSRPGT	
Zea_mayNP_001142394.1	VLTIAFQFSF	ENH---LRES	VAAMAKQYVR	GVMASVQRVA	MAIAPSRLGS	
Vit_vinREVOLUTA	VLTIAFQFPF	ESN---LQDN	VATMARQYVR	SVISSVQRVA	MAISPSGLGP	
Ory_braHOX32-like	VLTIAFQFSY	ENH---LRES	VAAMARQYVR	TVVASVQRVA	MAIAPSRLGG	
Ric_comXP_002529946.1	VLTIAFQFPF	ESN---LQEN	VATMARQYVR	SVISSVQRVA	MAISPSGLGP	
Tha_cacXP_007016749.1	VLTIALQFPF	DSN---LQDN	VAAMARQYVR	SVIASVQRVA	MAISPSGLSP	
Fra_vesREVOLUTA-like	VLTIAFQFPF	ESS---LQDN	VASMARQYVR	NVISSVQRVA	MAISPSGLSP	
Zea_mayNP_001146215.1	VLTIAFQFSF	ENH---LRES	VAAMARQYVR	AVMAIVQRVA	MAISPSRLGP	
The_cacXP_007012152.1	VMTIAFQFVY	EIH---LQEN	VATMARQYVR	SIIASVQRVA	LALSPSRFGS	
Pru_perppa001386mg	VMTIAFQFAF	EIH---LQDN	VASMARQYVR	SIIASVQRVA	LALSPSRFGS	
Pop_triAAX19057.1	VMTIAFQFAF	EMH---LQEN	VASMARQYVR	SIIASVQRVA	LALSPSHFGS	
Pop_triXP_002309538.2	VMTIAFQFAF	EMH---LQEN	VTSMARQYVR	SIIASVQRVA	LALSPSHFGS	
Fra_vesATHB-8-like	VMTIAFQFAF	EIH---LQDN	VASMARQYIR	SIIASVQRVA	LALSPSRFGS	
Pop_triXP_002324794.1	VMTIAFQFAF	EMH---LQEN	VASMARQYVR	SIIASVQRVA	LALSPSHFGS	
Vit_vin009744	VMTISFQFAF	EMH---LQEN	VASMARQYVR	SIISSVQRVA	LALSPSRFGP	
Gly_maxATHB-8-like	VMTIAFQFAF	EVH---LQEN	IATMARQYVR	SIIASVQRVS	LALSPSRFGS	
Sol_lycATHB-15-like	VMTIAFQFAF	EIH---LQES	IAAMARQYVR	SIISSVQRVA	LALSPSRIGS	
Gly_maxATHB-8-likeb	VMTIAFQFAF	EVH---LQEN	IATMARQYVR	SIIASVQRVS	LALSPSRFGS	
Med_truXP_003603630.1	VMTIAFQFAF	EAH---LQDN	IAAMARQYVR	SIVASVQRVS	LALSPSRFGS	

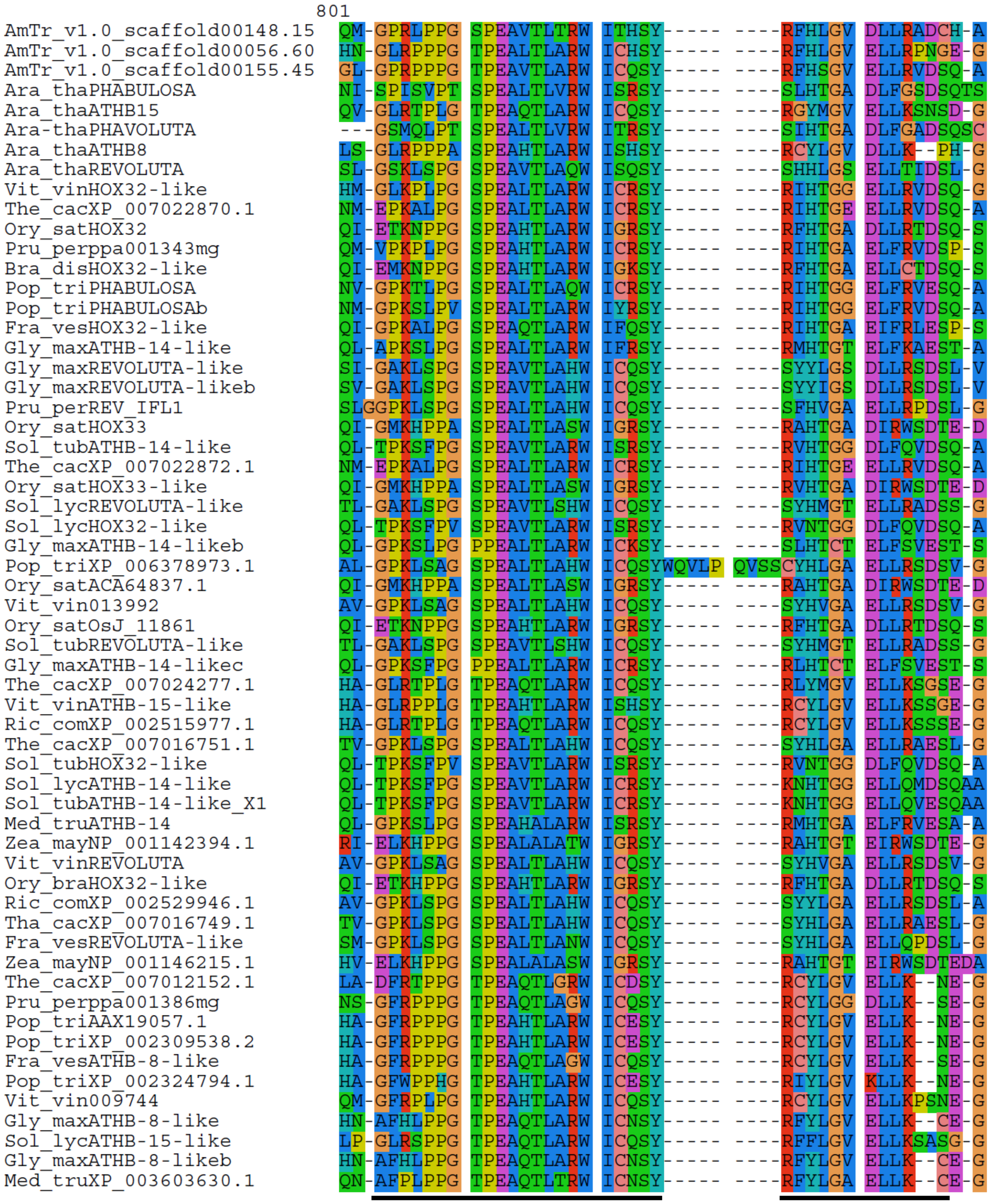
801
AmTr_v1.0_scaffold00148.15 QM-GPRLPPG SPEAVTLTRW ITHSY----- ----RFHLGV DLLRADCH-A
AmTr_v1.0_scaffold00056.60 HN-GLRPPPG TPEALTLARW ICHSY----- ----RFHLGV ELLRPNGE-G
AmTr_v1.0_scaffold00155.45 GL-GPRPPPG TPEAVTLARW ICQSY----- ----RFHSGV ELLRVDSQ-A
Ara_thaPHABULOSA NI-SPISVPT SPEALTLVRW ISRSY----- ----SLHTGA DLFGSDSQTS
Ara_thaATHB15 QV-GLRTPLG TPEAQTLARW ICQSY----- ----RGYMGV ELLKSNSD-G
Ara-thaPHAVOLUTA ---GSMQLPT SPEALTLVRW ITRSY----- ----SIHTGA DLFGADSQSC
Ara_thaATHB8 LS-GLRPPPA SPEAHTLARW ISHSY----- ----RCYLGV DLLK--PH-G
Ara_thaREVOLUTA SL-GSKLSPG SPEAVTLAQW ISQSY----- ----SHHLGS ELLTIDSL-G
Vit_vinHOX32-like HM-GLKPLPG SPEALTLARW ICRSY----- ----RIHTGG ELLRVDSQ-G
The_cacXP_007022870.1 NM-EPKALPG SPEALTLARW ICRSY----- ----RIHTGE ELLRVDSQ-A
Ory_satHOX32 QI-ETKNPPG SPEAHTLARW IGRSY----- ----RFHTGA DLLRTDSQ-S
Pru_perppa001343mg QM-VPKPLPG SPEAHTLARW ICRSY----- ----RIHTGA ELFRVDSP-S
Bra_disHOX32-like QI-EMKNPPG SPEAHTLARW IGKSY----- ----RFHTGA ELLCTDSQ-S
Pop_triPHABULOSA NV-GPKTLPG SPEALTLAQW ICRSY----- ----RIHTGG ELFRVESQ-A
Pop_triPHABULOSAb NM-GPKSLPV SPEALTLARW IYRSY----- ----RIHTGG ELFRVDSQ-A
Fra_vesHOX32-like QI-GPKALPG SPEAQTLARW IFQSY----- ----RIHTGA EIFRLESP-S
Gly_maxATHB-14-like QL-APKSLPG SPEALTLARW IFRSY----- ----RMHTGT ELFKAEST-A
Gly_maxREVOLUTA-like SI-GAKLSPG SPEAVTLAHW ICQSY----- ----SYYLGS DLLRSDSL-V
Gly_maxREVOLUTA-likeb SV-GAKLSPG SPEAVTLAHW ICQSY----- ----SYYIGS DLLRSDSL-V
Pru_perREV_IFL1 SLGGPKLSPG SPEALTLAHW ICQSY----- ----SFHVGA ELLRPDSL-G
Ory_satHOX33 QI-GMKHPPA SPEALTLASW IGRSY----- ----RAHTGA DIRWSDTE-D
Sol_tubATHB-14-like QL-TPKSFPG SPEAVTLARW ISRSY----- ----RVHTGG DLFQVDSQ-A
The_cacXP_007022872.1 NM-EPKALPG SPEALTLARW ICRSY----- ----RIHTGE ELLRVDSQ-A
Ory_satHOX33-like QI-GMKHPPA SPEALTLASW IGRSY----- ----RVHTGA DIRWSDTE-D
Sol_lycREVOLUTA-like TL-GAKLSPG SPEAVTLSHW ICQSY----- ----SYHMGT ELLRADSS-G
Sol_lycHOX32-like QL-TPKSFPV SPEAVTLARW ISRSY----- ----RVNTGG DLFQVDSQ-A
Gly_maxATHB-14-likeb QL-GPKSLPG PPEALTLARW ICKSY----- ----SLHTCT ELFSVEST-S
Pop_triXP_006378973.1 AL-GPKLSAG SPEALTLAHW ICQSYWQVLP QVSSCYHLGA ELLRSDSV-G
Ory_satACA64837.1 QI-GMKHPPA SPEALTLASW IGRSY----- ----RAHTGA DIRWSDTE-D
Vit_vin013992 AV-GPKLSAG SPEALTLAHW ICQSY----- ----SYHVGA ELLRSDSV-G
Ory_satOsJ_11861 QI-ETKNPPG SPEAHTLARW IGRSY----- ----RFHTGA DLLRTDSQ-S
Sol_tubREVOLUTA-like TL-GAKLSPG SPEAVTLSHW ICQSY----- ----SYHMGT ELLRADSS-G
Gly_maxATHB-14-likec QL-GPKSFPG PPEALTLARW ICRSY----- ----RLHTCT ELFSVEST-S
The_cacXP_007024277.1 HA-GLRTPLG TPEAQTLARW ICQSY----- ----RLYMGV ELLKSGSE-G
Vit_vinATHB-15-like HA-GLRPPLG TPEAHTLARW ISHSY----- ----RCYLGV ELLKSSGE-G
Ric_comXP_002515977.1 HA-GLRTPLG TPEAQTLARW ICQSY----- ----RCYLGV ELLKSSSE-G
The_cacXP_007016751.1 TV-GPKLSPG SPEALTLAHW ICQSY----- ----SYHLGA ELLRAESL-G
Sol_tubHOX32-like QL-TPKSFPV SPEAVTLARW ISRSY----- ----RVNTGG DLFQVDSQ-A
Sol_lycATHB-14-like QL-TPKSFPG SPEAVTLARW ICRSY----- ----KNHTGG ELLQMDSQAA
Sol_tubATHB-14-like_X1 QL-TPKSFPG SPEAVTLARW ICRSY----- ----KNHTGG ELLQVESQAA
Med_truATHB-14 QL-GPKSLPG SPEAHALARW ISRSY----- ----RMHTGA ELFRVESA-A
Zea_mayNP_001142394.1 RI-ELKHPPG SPEALALATW IGRSY----- ----RAHTGT EIRWSDTE-G
Vit_vinREVOLUTA AV-GPKLSAG SPEALTLAHW ICQSY----- ----SYHVGA ELLRSDSV-G
Ory_braHOX32-like QI-ETKHPPG SPEAHTLARW IGRSY----- ----RFHTGA DLLRTDSQ-S
Ric_comXP_002529946.1 AV-GPKLSPG SPEALTLAHW ICQSY----- ----SYYLGA ELLRSDSL-A
Tha_cacXP_007016749.1 TV-GPKLSPG SPEALTLAHW ICQSY----- ----SYHLGA ELLRAESL-G
Fra_vesREVOLUTA-like SM-GPKLSPG SPEALTLANW ICQSY----- ----SYHLGA ELLQPDSL-G
Zea_mayNP_001146215.1 HV-ELKHPPG SPEALALASW IGRSY----- ----RAHTGT EIRWSDTEDA
The_cacXP_007012152.1 LA-DFRTPPG TPEAQTLGRW ICDSY----- ----RCYLGV ELLK--NE-G
Pru_perppa001386mg NS-GFRPPPG TPEAQTLAGW ICQSY----- ----RCYLGG DLLK--SE-G
Pop_triAAX19057.1 HA-GFRPPPG TPEAHTLARW ICESY----- ----RCYLGV ELLK--NE-G
Pop_triXP_002309538.2 HA-GFRPPPG TPEAHTLARW ICESY----- ----RCYLGV ELLK--NE-G
Fra_vesATHB-8-like HA-GFRPPPG TPEAQTLAGW ICQSY----- ----RCYLGV ELLK--SE-G
Pop_triXP_002324794.1 HA-GFWPPHG TPEAHTLARW ICESY----- ----RIYLGV KLLK--NE-G
Vit_vin009744 QM-GFRPLPG TPEAHTLARW ICQSY----- ----RCYLGV ELLKPSNE-G
Gly_maxATHB-8-like HN-AFHLPPG TPEAQTLARW ICNSY----- ----RFYLGV ELLK--CE-G
Sol_lycATHB-15-like LP-GLRSPPG TPEAQTLARW ICQSY----- ----RFFLGV ELLKSASG-G
Gly_maxATHB-8-likeb HN-AFHLPPG TPEAQTLARW ICNSY----- ----RFYLGV ELLK--CE-G
Med_truXP_003603630.1 QN-AFPLPPG TPEAQTLTRW ICNSY----- ----RFYLGA ELLK--CE-G

```
                               851
AmTr_v1.0_scaffold00148.15     -GDSVMKLLW HYSDAIMCCS LK----AL-P VFT--FANQA GLDMLETTLV
AmTr_v1.0_scaffold00056.60     -GESLLKMLW HHADAIMCCS LK----AL-P VFT--FANQA GLDMLETTLV
AmTr_v1.0_scaffold00155.45     -GDSLLKLLW HHSDAIMCCS LK----AS-P IFS--FSNQA GLDMLETTLV
Ara_thaPHABULOSA               -GDTLLHQLW NHSDAILCCS LKTN--AS-P VFT--FANQT GLDMLETTLV
Ara_thaATHB15                  -NESILKNLW HHTDAIICCS MK----AL-P VFT--FANQA GLDMLETTLV
Ara-thaPHAVOLUTA               GGDTLLKQLW DHSDAILCCS LKTN--AS-P VFT--FANQA GLDMLETTLV
Ara_thaATHB8                   -TD-LLKSLW HHPDAVMCCS LK----ALSP VFT--FANQA GLDMLETTLV
Ara_thaREVOLUTA                SDDSVLKLLW DHQDAILCCS LK----PQ-P VFM--FANQA GLDMLETTLV
Vit_vinHOX32-like              -GDAVLKLLW NHSDAIMCCS LKTN--AS-P VFT--FANQA GLDMLETTLV
The_cacXP_007022870.1          -GDALLKQLW HHSDAIMCCS LKTN--AS-P VFT--FANQA GLDMLETTLV
Ory_satHOX32                   -TDSSLKAMW QHSDSIMCCS LK----AA-P VFT--FANQA GLDMLETTLI
Pru_perppa001343mg             -GDAVLKQLW HHSDAIMCCS VKTN--AS-P VFT--FANQA GLDMLETTLV
Bra_disHOX32-like              -ADASLKALW QHSDSIMCCS LK----AA-P VFT--FANQA GLDMLETTLI
Pop_triPHABULOSA               -GDALLKQLW HHSDAIMCCS LKTN--AS-P VFT--FANQA GLDMLETTLV
Pop_triPHABULOSAb              -GDALLKRLW HHSDAIMCCS LKTN--AS-P VFT--FANQA GLDMLETTLV
Fra_vesHOX32-like              -GDAVLKQLW NHPDAIMCCS VKTN--AS-P VFT--FANQA GLDMLETTLV
Gly_maxATHB-14-like            -GDAILKQLW HHPDAIMCCS VKTN--AS-P VFT--FANQA GLDMLETTLV
Gly_maxREVOLUTA-like           -GDMMLKQLW HHQDAILCCS LK----SL-P VFI--FANQA GLDMLETTLV
Gly_maxREVOLUTA-likeb          -GDMMLKQLW HHQDAILCCS LK----PL-P VFI--FANQA GLDMLETTLV
Pru_perREV_IFL1                -GDSMLKHLW HHQDAILCCS LK----SL-P VFI--FANQA GLDMLETTLV
Ory_satHOX33                   -ADSPLALLW KHSDAILCCS LK----PA-P MFT--FANNA GLDILETTLV
Sol_tubATHB-14-like            -GDAVLKQLW HHSDAIMCCS VKMN--AS-A VFT--FANQA GLDMLETTLL
The_cacXP_007022872.1          -GDALLKQLW HHSDAIMCCS LKTN--AS-P VFT--FANQA GLDMLETTLV
Ory_satHOX33-like              -ADSPLALLW KHNDAILCCS LK----PA-P MFT--FANNA GLDILETTLV
Sol_lycREVOLUTA-like           -DESVLKNLW QHQDAILCCS LK----SL-P VFI--FANKA GLDMLETTLV
Sol_lycHOX32-like              -GDAVLKQLW HHSDAIMCCS VKMNVSAS-A VFT--FANQA GLDMLETTLL
Gly_maxATHB-14-likeb           -GDAILKQLW HHPDAILCCS VKTN--AS-P VFT--FANQA GLDMLETTLV
Pop_triXP_006378973.1          -GDSVLKHLW HHPDAILCCS LE----AL-P VFI--FANQA GLDMLETTLV
Ory_satACA64837.1              -ADSPLALLW KHSDAILCCS LK----PA-P MFT--FANNA GLDILETTLV
Vit_vin013992                  -GDSVLKNLW HHQDAILCCS LK----SL-P VLI--FANQA GLDMLETTLV
Ory_satOsJ_11861               -TDSSLKAMW QHSDSIMCCS LK----AA-P VFT--FANQA GLDMLETTLI
Sol_tubREVOLUTA-like           -DESVLKNLW QHQDAILCCS LK----SL-P VFI--FANKA GLDMLETTLV
Gly_maxATHB-14-likec           -GDAILKQLW HHPDAILCCS VKTD--AS-P VFT--FANQA GLDMLETTLV
The_cacXP_007024277.1          -SETILKTLW HHSDAIMCCS LK----AL-P VFT--FANQA GLDMLETTLV
Vit_vinATHB-15-like            -SETILKTLW HLSDAIMCCS LK----AL-P VFT--FANQA GLDMLETTLV
Ric_comXP_002515977.1          -GESILKTLW HHSDAIMCCS LK----AL-P VFT--FANQA GLDMLETTLV
The_cacXP_007016751.1          -GDAVLKNLW QHQDAILCCS LK----SL-P VFI--FANQA GLDMLETTLV
Sol_tubHOX32-like              -GDAVLKQLW HHSDAIMCCS VKMNVSAS-A VFT--FANQA GLDMLETTLL
Sol_lycATHB-14-like            -GDAVLKQLW HHTDSIMCCS VKMN--DS-A FFS--FANQA GLDMLETTLI
Sol_tubATHB-14-like_X1         -GDAVLKQLW HHTDSIMCCS VKMN--DS-A VFS--FSNQA GLDMLETTLI
Med_truATHB-14                 -SDAILKQLW HHSDAIMCCS VKTN--AS-P IFT--FSNQA GLDMLETTLV
Zea_mayNP_001142394.1          -ADSPLMLFW KHSDAILCCS LK----P--P AFTLKFANSA GFDILETTMV
Vit_vinREVOLUTA                -GDSVLKNLW HHQDAILCCS LK----SL-P VLI--FANQA GLDMLETTLV
Ory_braHOX32-like              -TDSSLKAMW QHSDSIMCCS LK----AA-P VFT--FANQA GLDMLETTLI
Ric_comXP_002529946.1          -GDSVLKQLW HHQDAILCCS LK----SL-P VFI--FANQA GLDMLETTLV
Tha_cacXP_007016749.1          -GDAVLKNLW QHQDAILCCS LK----SL-P VFI--FANQA GLDMLETTLV
Fra_vesREVOLUTA-like           -GDSMLKHLW HHQDAILCCS LK----SV-P VFI--FANQA GLDMLETTLV
Zea_mayNP_001146215.1          -AGSPLTLFW KHSDAIICCS LK-------P AFTLKFANSA GFDILETTVA
The_cacXP_007012152.1          -SESILKMLW HHTDAVLCCS LK----AL-P VFT--FANQA GLDMLETTLV
Pru_perppa001386mg             -SESILKSLW HHSDAILCCS LK----AL-P VFT--FANQA GLDMLETTLV
Pop_triAAX19057.1              -SESILKTLW HHSDALMCCS LK----TL-P VFT--FANQA GLDMLETTLV
Pop_triXP_002309538.2          -SESILKTLW HHSDALMCCS LK----TL-P VFT--FANQA GLDMLETTLV
Fra_vesATHB-8-like             -SESILKSLW HHSDAILCCS LK----AL-P VFT--FANQA GLDMLETTLV
Pop_triXP_002324794.1          -SDSILKTLW HHSDALVCCS LK----TL-P VFT--FSNQA GLDMLETTLV
Vit_vin009744                  -NESILKTLW HHSDAVMCCS LK----AL-P VFT--FANQA GLDMLETTLV
Gly_maxATHB-8-like             -SESILKSLW HHSDAVLCCS LK----AL-P VFT--FANQA GLDMLETTLV
Sol_lycATHB-15-like            -SESILKEIW DHSDALMCCS MK----AL-P VFT--FANEA GLDMLETTLV
Gly_maxATHB-8-likeb            -SESILKSLW HHSDAVLCCS LK----AL-P VFT--FANQA GLDMLETTLV
Med_truXP_003603630.1          -SESILKSLW HHSDAVLCCS LK----AL-P VFT--FANQA GLDMLETTLV
```

```
                              901
AmTr_v1.0_scaffold00148.15    ALQDISLDKI LD-EGGRKSL CSDFTKIMQQ ---GYTYLPA GICVSSMGRP
AmTr_v1.0_scaffold00056.60    ALQDITLEKI FD-ENGRKTL CADFAQIMQQ ---GFAYLQG GLCVSSMGRP
AmTr_v1.0_scaffold00155.45    ALQDITLDKI LD-ENGRKVL YSEFPKIMQQ ---GFAYLPA GICVSSMGRP
Ara_thaPHABULOSA              ALQDIMLDKT LD-EPGRKAL CSEFPKIMQQ ---GYAHLPA GVCASSMGRM
Ara_thaATHB15                 ALQDISLEKI FD-DNGRKTL CSEFPQIMQQ ---GFACLQG GICLSSMGRP
Ara-thaPHAVOLUTA              ALQDIMLDKT LD-DSGRRAL CSEFAKIMQQ ---GYANLPA GICVSSMGRP
Ara_thaATHB8                  ALQDITLDKI FDNNNGKKTL SSEFPQIMQQ ---GFMCMDG GICMSSMGRA
Ara_thaREVOLUTA               ALQDITLEKI FD-ESGRKAI CSDFAKLMQQ ---GFACLPS GICVSTMGRH
Vit_vinHOX32-like             ALQDIMLDKI LD-EAGRKIL CSEFSKIMQQ ---GFAYLPA GICTSSMGRP
The_cacXP_007022870.1         ALQDIMLDKI LD-EAGRKIL CSEFSKIMQQ ---GFAYLPA GICVSSMGRP
Ory_satHOX32                  ALQDISLEKI LD-DDGRKAL CTEFPKIMQQ ---GFAYLPG GVCVSSMGRP
Pru_perppa001343mg            ALQDIMLDKI LD-EAGRKIL CSEFSKIMQQ ---GFAYLPA GLCASSMGRP
Bra_disHOX32-like             ALQDISLEKI LD-DDGRKAL CSEFPKIMQQ ---GFAYLPG GVCVSSMGRP
Pop_triPHABULOSA              ALQDIMLDKI LD-EAGRKIL CSEFSKITQQ ---GFAYLPA GICVSSMGRP
Pop_triPHABULOSAb             ALQDIMLDKI LD-EAGRKIL CSEFSKIMQQ ---GYAYLPA GICVSSMGRP
Fra_vesHOX32-like             ALQDIMLDKI LD-EAGRKVL CSEFSKIMLQ ---GFAFLPA GMCASSMGRP
Gly_maxATHB-14-like           ALQDIMLDKV LD-ESSRKIL CCEFSKIMQQ ---GFAYLPA GICASSMNRP
Gly_maxREVOLUTA-like          ALQDITLDKI FD-EAGRKAL CTDFAKLMEQ ---GFAYLPA GICMSTMGRH
Gly_maxREVOLUTA-likeb         ALQDITLDKI FD-EAGRKAL CTDFAKLMEQ ---GFAYLPA GICMSTMGRH
Pru_perREV_IFL1               ALQDISLDKI FD-ECGRKTL CADFAKLMNQ ---GFAHLPA GICMSTMGRH
Ory_satHOX33                  NLQDISLEMI LD-DEGRKAL CSEFPKIMQQ ---GFTYLPG GVCKSSMGRQ
Sol_tubATHB-14-like           ALQDIMLDKI LD-EAGRKVL LSEFSKIMQQ ---GFAYLPA GICVSSMGRP
The_cacXP_007022872.1         ALQDIMLDKI LD-EAGRKIL CSEFSKIMQQ ----VKFSVS GLCSSD----
Ory_satHOX33-like             NLQDISLEMI LD-DEGRKAL CSEFPKIMQQ ---GFTYLPG GVCKSSMGRQ
Sol_lycREVOLUTA-like          ALQDISLDKI FD-ESGRKVL LSEFAKIMEQ ---GFACLPG GICMSTMGRH
Sol_lycHOX32-like             ALQDIMLDKI LD-EAGRKVL LSEFSKIMQQ ---GFAYLPA GICVSSMGRP
Gly_maxATHB-14-likeb          ALQDIMLDKV LD-EAGRKFL CIEFSKIMQQ ---GFAYLPA GICVSSMNRP
Pop_triXP_006378973.1         ALQDITLDKI FD-ESGRKAL FTDFAKLMQQ ANNGFACLPA GICMSTMGRN
Ory_satACA64837.1             NLQDISLEMI LD-DEGRKAL CSEFPKIMQQ ---GFTYLPG GVCKSSMGRQ
Vit_vin013992                 ALQDISLDKI FD-ESGRKGL CADFAKIMQQ ---GFAYLPA GICMSTMGRH
Ory_satOsJ_11861              ALQDISLEKI LD-DDGRKAL CTEFPKIMQQ ---GFAYLPG GVCVSSMGRP
Sol_tubREVOLUTA-like          ALQDISLDKI FD-EPGREVL LSEFAKIMEQ ---GFACLPG GICMSTMGRH
Gly_maxATHB-14-likec          ALQDIMLDKV LD-EAGRKVL CIEFSKIMQQ ---GFAYLPA GICVSSMNRP
The_cacXP_007024277.1         ALQDITLEKI FD-DHGRKTL CTEFPQIMQQ ---GFACLQG GICLSSMGRP
Vit_vinATHB-15-like           ALQDITLEKI FD-DHGRKTL CSEFPQIMQQ ---GFACLQG GICLSSMGRP
Ric_comXP_002515977.1         ALQDITLEKI FD-DHGRKTL CSEFPQIMQQ ---GFACLQG GICLSSMGRP
The_cacXP_007016751.1         ALQDITLDKI FD-ESGRKAL CSDFAKLMQQ ---------- ----------
Sol_tubHOX32-like             ALQDIMLDKI LD-EAGRKVL LSEFSKIMQQ ---GFAYLPA GICVSSMGRP
Sol_lycATHB-14-like           ALQDIMLDKI LD-EAGRKVL LSEFPKIMQQ ---GFAYLPA GLCVSSMGRP
Sol_tubATHB-14-like_X1        ALQDIMLDKI LD-EAGRKVL LSEFPKIMQQ ---GFAYLPA GLCVSSMGRP
Med_truATHB-14                ALQDIMLDKV LD-EAGRKIL CSEFSKIMQQ ---GYASLPG GICVSSMNRP
Zea_mayNP_001142394.1         NIQDLPLEAV LD-EEGQKAL SAQLPAIMQQ ---GLAYLPG GVCRSSMGRQ
Vit_vinREVOLUTA               ALQDISLDKI FD-ESGRKGL CADFAKIMQQ ---GFAYLPA GICMSTMGRH
Ory_braHOX32-like             ALQDISLEKI LD-DDGRKAL CSEFPKIMQQ ---GLAYLPG GVCVSSMGRP
Ric_comXP_002529946.1         ALQDITLDKI FD-ESGRKAL CADFAKLMQQ ---GFAGLPG GICMSTMGRH
Tha_cacXP_007016749.1         ALQDITLDKI FD-ESGRKAL CSDFAKLMQQ ---GFAYLPA GICMSTMGRN
Fra_vesREVOLUTA-like          ALQDISLDKI FD-ESGRKTL CTDFAKLMQQ ---GFAYLPA GICMSTMGRH
Zea_mayNP_001146215.1         NVQDLQLEAV LD-DGGQKAL VAQLPKIMLQ ---GLAYLPG GVCRSSMGRQ
The_cacXP_007012152.1         ALQDISLEKI FD-ENGRKAL FAEFPQVMQQ ---GFMCLQG GICLSSMGRP
Pru_perppa001386mg            ALQDITLEKI FD-DNGRKTL FSEFPQIMQQ ---GFMCLQG GICMSSMGRP
Pop_triAAX19057.1             ALQDITLEKI FD-DNGRKTL YSEFPQIMQQ ---GFMCLQG GICLSSMGRP
Pop_triXP_002309538.2         ALQDITLEKI FD-DNGRKTL YSEFPQIMQQ ---GFMCLQG GICLSSMGRP
Fra_vesATHB-8-like            ALQDITLEKI FD-DNGRKIL FTEFPQIMQQ ---GFMCLQG GICMSSMGRP
Pop_triXP_002324794.1         ALQDITLEKI FD-DNGRKTL CSEFPQIMQQ ---GFMCLPG GICLSSMGRP
Vit_vin009744                 ALQDITLEKT FD-DNGRKTL CSEFPQIMQQ ---GFVCLQG GVCLSSMGRP
Gly_maxATHB-8-like            ALQDITLEKI FD-DNGKKTL CTEFPQIMQQ ---GFMCIQG GICLSSMGRP
Sol_lycATHB-15-like           ALQDISLEKI FD-DNGRKAL YSELPQIMQQ ---GFACLQG GICLSSMGRP
Gly_maxATHB-8-likeb           ALQDITLEKI FD-DNGKKTL CTEFPQIMQQ ---GFMCIQG GICLSSMGRP
Med_truXP_003603630.1         ALQDITLEKI FD-DNGKKTL CSEFPQIMQQ ---GFMCIQG GICLSSMGRP
```

```
                              951
AmTr_v1.0_scaffold00148.15    VSYDQAIAWK VL--TEEEAT --HCLAFMFI NWSFV
AmTr_v1.0_scaffold00056.60    VSYERAVAWK VL--NEEEST --HCICFMFM NWSFV
AmTr_v1.0_scaffold00155.45    VSYEQAVAWK VL--NEDDCT --HCLAFMFL NWSFV
Ara_thaPHABULOSA              VSYEQATVWK VL--EDDESN --HCLAFMFV NWSFV
Ara_thaATHB15                 VSYERAVAWK VL--NEEENA --HCICFVFI NWSFV
Ara-thaPHAVOLUTA              VSYEQATVWK VV--DDNESN --HCLAFTLV SWSFV
Ara_thaATHB8                  VTYEKAVGWK VL--NDDEDP --HCICFMFL NWSFI
Ara_thaREVOLUTA               VSYEQAVAWK VFAASEENNN NLHCLAFSFV NWSFV
Vit_vinHOX32-like             VSYEQAIAWK VL--NDEDSN --HCLAFMFI NWSFV
The_cacXP_007022870.1         VSYEQAIAWK VL--NDNDAN --HCLTFMFV NWSFV
Ory_satHOX32                  VSYEQAVAWK VL--SDDDTP --HCLAFMFV NWSFV
Pru_perppa001343mg            VSYDQAVAWK VV--NDDDSN --HCLAFMFM SWSFV
Bra_disHOX32-like             VSYEQAVAWK VL--SEDDTP --HCLAFMFV NWSFV
Pop_triPHABULOSA              VSYEQAVAWK VL--NDDDSN --HCLAFMFM NWSFV
Pop_triPHABULOSAb             VSYEQAIAWK VL--NDDNSN --HCLAFMFI NWSFV
Fra_vesHOX32-like             VSYEQAVAWK VV--NDEDSN --HCLALMFT NWSFV
Gly_maxATHB-14-like           VSYEQAVAWK VL--NDDDSN --HCLAFMFM NWSFV
Gly_maxREVOLUTA-like          VSYDQAIAWK VLT-GEDNTV --HCLAFSFI NWSFV
Gly_maxREVOLUTA-likeb         VSYDQAIAWK VLT-GEDNTV --HCLAFSFI NWSFV
Pru_perREV_IFL1               VSYEQAVAWK VLA-AEENSV --HCLAFSFM NWSFV
Ory_satHOX33                  ASYEQAVAWK VL--SDDDAP --HCLAFMLV NWTFM
Sol_tubATHB-14-like           ISYEQAIAWK VL--NDDDSN --HCLAFMFI NWSFV
The_cacXP_007022872.1         ---------- ---------- ----IEFFPS N----
Ory_satHOX33-like             ASYEQAVAWK VL--SDDDAP --HCLAFMLV NWTFM
Sol_lycREVOLUTA-like          ISYEQAIAWK VFASSEENAV --HCLAFSFI NWSFV
Sol_lycHOX32-like             ISYEQAIAWK VL--NDDDSN --HCLAFMFI NWSFV
Gly_maxATHB-14-likeb          VSYEQAIAWK VL--DDDDSN --HCLAFVFM NWSFV
Pop_triXP_006378973.1         VSYEQAVSWK VLA-AEENTV --HCIAFSFV NWSFL
Ory_satACA64837.1             ASYEQAVAWK VL--SDDDAP --HCLAFMLV NWTFM
Vit_vin013992                 VSYEQAIAWK VLA-AEENTV --HCLAFSFI NWSFV
Ory_satOsJ_11861              VSYEQAVAWK VL--SDDDTP --HCLAFMFV NWSFV
Sol_tubREVOLUTA-like          ISYEQAIAWK VFASSEENAV --HCLAFSFI NWSFV
Gly_maxATHB-14-likec          VSYEQAIAWK VL--DDDDSN --HCLAFVFM NWSFV
The_cacXP_007024277.1         VSYERAVAWK VL--NEEENA --HCICFMFI NWSFV
Vit_vinATHB-15-like           VSYERAVAWK VL--NEEENA --HCVCFMFM NWSFV
Ric_comXP_002515977.1         VSYERAVAWK VL--NEEENA --HCICFMFI NWSFV
The_cacXP_007016751.1         ---------- -------VSP --N------- -----
Sol_tubHOX32-like             ISYEQAIAWK VL--NDDDSN --HCLAFMFI NWSFV
Sol_lycATHB-14-like           VSYEQAVAWK VL--NDDNSN --HCLAFMFT NWSFI
Sol_tubATHB-14-like_X1        VSYEQAVAWK VL--NDDNSS --HCLAFMFT NWSFI
Med_truATHB-14                VSYEQAIAWK VL--NDDDAN --HCLAFMFI NWSFV
Zea_mayNP_001142394.1         ASYEQAVAWK VV--GDDGAP --QCLALMLA NWTFI
Vit_vinREVOLUTA               VSYEQAIAWK VLA-AEENTV --HCLAFSFI NWSFV
Ory_braHOX32-like             VSYEQAVAWK VL--SDDDTP --HCLAFMFV NWSFV
Ric_comXP_002529946.1         VSYEQAVAWK VLA-ADESTV --HCLAFSFV NWSFV
Tha_cacXP_007016749.1         VSYEQAVAWK VLA-ADESTV --HCLAFSFV NWSFV
Fra_vesREVOLUTA-like          VSYEQAIGWK VLS-GEDNSV --HCLAFSFV NWSFV
Zea_mayNP_001146215.1         ASYEQAVAWK VV--GDDGAP --QCLALMFV NWTFI
The_cacXP_007012152.1         VSYERAVAWK VV--NDEENA --HCICFMFI NWSFV
Pru_perppa001386mg            ISYERAVAWK VL--NEEETA --HCICFMFI NWSFV
Pop_triAAX19057.1             VSYERAVAWK VL--NEEESA --HCICFMFI NWSFV
Pop_triXP_002309538.2         VSYERAVAWK VL--NEEESA --HCICFMFI NWSFV
Fra_vesATHB-8-like            ISYERAVAWK VL--NEEETA --HCICFMFI NWSFV
Pop_triXP_002324794.1         VSYERAVAWK VL--NEEESA --HCICFMFM NWSFV
Vit_vin009744                 VSYERAVAWK VL--TEEDNA --HCICFMFI NWSFV
Gly_maxATHB-8-like            VSYERAVAWK VL--NEEESA --HCICFMFI NWSFV
Sol_lycATHB-15-like           ISYERAVAWK VL--NEEEDA --HCICFMFI NWSFV
Gly_maxATHB-8-likeb           VSYERAVAWK VL--NEEESA --HCICFMFI NWSFV
Med_truXP_003603630.1         ITYERAVAWK VL--NEEESA --HCICFMFI NWSFV
```
