# Supplementary material for: Evidence for the Extensive Conservation of Mechanisms of Ovule Integument Development Since the Most Recent Common Ancestor of Living Angiosperms
Source: Front Plant Sci. 2018 Sep 19;9:1352. doi: 10.3389/fpls.2018.01352 (PMC6156155; doi:10.3389/fpls.2018.01352)
Supplement: TABLE S1 — Oligonucleotide primers used to amplify Amborella integument regulators. [file Table_1.pdf]

Supplementary Table 1. Oligonucleotide primers used to amplify *Amborella* integument regulators

AtrINO/F	5’-ATGTCTTCATGTGAGCAC
AtrINO/R-T7	5’-tgtaatacgactcactatagggcTCACTCATTGTTTTCCATG
AtrATS/F	5’-ATGGACATGAGGGCTGGGCA
AtrATS/R-T7	5’-tgtaatacgactcactatagggcTTAGCATTTAAGAAGTGCGA
AtrPHB-PHV/F	5’-ATGGCGCTTGTCTTGCAAAA
AtrPHB-PHV/R-T7	5’-tgtaatacgactcactatagggcCTAAACAAAAGACCAGTTGA
AtrREV/F	5’-ATGGCAGTAGCTGTACATAA
AtrREV/R-T7	5’-tgtaatacgactcactatagggcTCAAACGAAGGACCAGTTGA
AtrARF4/F	5’-ATGGAAATTGATCTCAAC
AtrARF4/R-T7	5’-tgtaatacgactcactatagggcCTAACGATGAGCATACTTTG
AtrETT/F	5’-ATGGGCATTGATCTGAACCG
AtrETT/R-T7	5’-tgtaatacgactcactatagggcTTACACAGCTCTAGCAAGGC

Sequences shown in lower case on reverse PCR primers correspond to the T7 RNA Polymerase promoter, used subsequently for riboprobe synthesis as described in Vialette-Guiraud et al. (2011).
